# Reactivity-dependent profiling of RNA 5-methylcytidine dioxygenases

**DOI:** 10.1038/s41467-022-31876-2

**Published:** 2022-07-19

**Authors:** A. Emilia Arguello, Ang Li, Xuemeng Sun, Tanner W. Eggert, Elisabeth Mairhofer, Ralph E. Kleiner

**Affiliations:** Department of Chemistry, Princeton University, Princeton, NJ 08544 USA

**Keywords:** RNA, Transcriptomics

## Abstract

Epitranscriptomic RNA modifications can regulate fundamental biological processes, but we lack approaches to map modification sites and probe writer enzymes. Here we present a chemoproteomic strategy to characterize RNA 5-methylcytidine (m^5^C) dioxygenase enzymes in their native context based upon metabolic labeling and activity-based crosslinking with 5-ethynylcytidine (5-EC). We profile m^5^C dioxygenases in human cells including ALKBH1 and TET2 and show that ALKBH1 is the major hm^5^C- and f^5^C-forming enzyme in RNA. Further, we map ALKBH1 modification sites transcriptome-wide using 5-EC-iCLIP and ARP-based sequencing to identify ALKBH1-dependent m^5^C oxidation in a variety of tRNAs and mRNAs and analyze ALKBH1 substrate specificity in vitro. We also apply targeted pyridine borane-mediated sequencing to measure f^5^C sites on select tRNA. Finally, we show that f^5^C at the wobble position of tRNA-Leu-CAA plays a role in decoding Leu codons under stress. Our work provides powerful chemical approaches for studying RNA m^5^C dioxygenases and mapping oxidative m^5^C modifications and reveals the existence of novel epitranscriptomic pathways for regulating RNA function.

## Abstract (web summary)

Kleiner and co-workers profile RNA 5-methylcytidine (m^5^C) dioxygenase enzymes using an activity-based metabolic probing strategy. They reveal ALKBH1 as the major 5-formylcytidine (f^5^C) writer and characterize modification sites across mRNA and tRNA.

## Introduction

Chemical modifications on central biomolecules play critical roles in biology. RNA is extensively modified by a diverse collection of post-transcriptional modifications (known as the “epitranscriptome”) that can affect RNA structure, base pairing, and protein-RNA interactions. In turn, these biochemical perturbations have been shown to control RNA metabolism, splicing, protein translation, and RNA localization, thereby affecting gene expression and higher-order physiological processes^1–3^. Over 150 chemical marks have been identified on various types of RNA spanning all kingdoms of life^4^, but we still lack insight into the function and distribution of many modifications. A number of RNA modifications have also been implicated in human disease, underscoring the importance of investigating epitranscriptomic processes in biology^5,6^.

Studies of epitranscriptomic modifications in their native context typically require transcriptome-wide mapping together with characterization of writer, eraser, and reader proteins. These data can then motivate the generation and interrogation of functional hypotheses regarding the biological role of individual modifications. Modification mapping is a major challenge since many RNA modifications are “silent” in standard Next-Generation Sequencing (NGS) analysis and present at low stoichiometry^7^. Therefore, specialized workflows relying upon modification-specific antibodies or selective chemoenzymatic transformations need to be developed for each modification, and as a consequence reliable mapping data is only available for a few RNA modifications^7^. Further, identifying relevant modification-associated enzymes and profiling their substrates is an additional obstacle and these enzymes are typically deduced based upon screening or homology to known enzymes.

One of the more abundant modifications found in cellular RNA is 5-methylcytidine (m^5^C). In eukaryotes, this modification is installed by NSUN/DNMT proteins on diverse RNA species^8–10^, and it can affect a broad range of cellular processes such as translation, stress response, mRNA nuclear-cytoplasmic trafficking, RNA stability, splicing, and DNA damage repair^11^. In addition, oxidation of m^5^C can generate hm^5^C and f^5^C in RNA^12–15^. Recent work has used antibody-based sequencing to map hm^5^C in mRNA and tRNA^16–18^, but the identity of hm^5^C writer enzymes has not been fully elucidated and we lack nucleotide-resolution sequencing for this modification. For f^5^C, transcriptome-wide sequencing in mammalian systems has not been reported, but mass spectrometry has revealed sites at the wobble position of cytosolic tRNA-Leu-CAA and mitochondrial (mt)-tRNA-Met generated by the Fe(II)/α-KG-dependent dioxygenase ALKBH1^19,20^. Further investigation into these oxidized derivatives has been hampered by the lack of tools to confidently sequence these marks and to systematically profile the writers involved in their formation.

To address this gap, our group recently developed RNA-mediated activity-based protein profiling (RNABBP)^21^, a reactivity-based proteomic strategy to profile RNA modifying enzymes in living cells. Here, we present an RNABBP approach to survey the human proteome for m^5^C oxidizing enzymes (i.e., writers of hm^5^C and f^5^C). Our strategy relies upon metabolic labeling with the m^5^C-mimicking warhead 5-ethynylcytidine (5-EC)^22^, oligo(dT)-based RNA-protein enrichment, and quantitative mass spectrometry. Our results show that ALKBH1 is the major m^5^C-oxidizing enzyme in HEK293T cells. Further, we use nucleoside mass spectrometry to characterize the prevalence of hm^5^C and f^5^C on human RNA and show that human mRNA contains f^5^C modification. Finally, we develop 5-EC-aided individual-nucleotide CLIP sequencing (iCLIP) and pyridine-borane chemical sequencing of f^5^C to characterize hm^5^C/f^5^C sites generated by ALKBH1 on tRNA. We validate some of these sites orthogonally with Aldehyde Reactive Probe (ARP)-based sequencing and further propose a potential role of f^5^C in tRNA-Leu-CAA during translation. Taken together, our work expands the generality of RNABPP and deepens our understanding of oxidative m^5^C modifications in the human epitranscriptome.

## Results

### Metabolic labeling and protein crosslinking with 5-ethynylcytidine (5-EC)

**Fig. 1:**
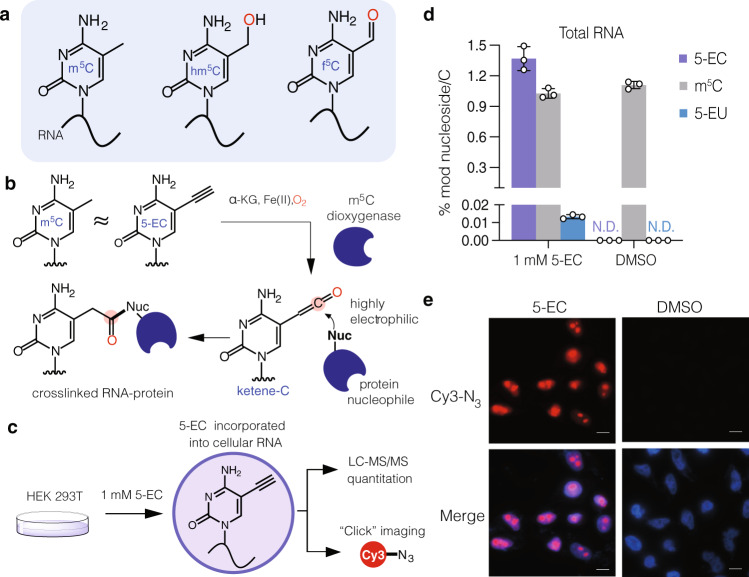
Probing m^5^C oxidation in RNA with 5-ethynylcytidine (5-EC). **a** Structure of reported m^5^C oxidation products in RNA. **b** Proposed capture of m^5^C dioxygenases by metabolic labeling with 5-EC. 5-EC mimics m^5^C in RNA and upon enzymatic oxidation generates an electrophilic ketene that can covalently trap nearby nucleophiles. Source data are provided as a Source Data file. **c** Strategy for metabolic labeling with 5-EC. The nucleoside is fed to mammalian cells and incorporated into cellular RNA. Incorporation can be measured by quantitative mass spectrometry or click chemistry imaging. **d** LC-MS/MS analysis of 5-EC, m^5^C, and 5-EU in total RNA of HEK293T cells after treatment with 1 mM 5-EC or DMSO for 16 h. Three independent biological replicates were analyzed. Data represent mean values ± s.d. **e** Fluorescence microscopy analysis of 5-EC cellular incorporation. HEK293T were treated with 1 mM 5-EC or DMSO for 16 h, fixed and labeled with Cy3-N_3_ before imaging by fluorescence microscopy. The experiments were repeated three times independently with similar results. Scale bar = 10 µM.

To profile m^5^C RNA dioxygenases using RNABPP (Fig. 1a, b), we needed a reactive probe that could be efficiently incorporated into cellular RNA and act as an m^5^C mimic. We chose 5-ethynylcytidine (5-EC) based on the ability of its deoxynucleoside analog (5-EdC) to covalently label TET enzymes in an activity-based manner on DNA^23^. Further, 5-EC has been used for RNA metabolic labeling experiments to monitor RNA transcription^22^, similar to the more widely used 5-ethynyluridine (5-EU) nucleoside^24^. Therefore, we hypothesized that metabolically incorporated 5-EC would be able to react in an activity-dependent manner with RNA m^5^C dioxygenases (Fig. 1b).

To confirm efficient metabolic labeling with 5-EC, we synthesized 5-EC (Supplementary Information), treated HEK293T cells, and measured incorporation by nucleoside LC-MS/MS and click chemistry imaging (Fig. 1c, Supplementary Figs. 1–2, Supplementary Tables 1–2). We observed levels of 1.37 ± 0.096% 5-EC/C with 1 mM 5-EC overnight treatment (Fig. 1d), a concentration that balances labeling efficiency with cytotoxicity (Supplementary Fig. 3). We also detected low levels of 5-EU (0.013 ± 0.003% of C) in total RNA from 5-EC-fed cells, likely due to deamination in cells. Levels of endogenous m^5^C or its oxidative derivative 5-formylcytidine (f^5^C) were largely unperturbed by 5-EC feeding (Fig. 1c, Supplementary Fig. 4, Supplementary Tables 2, 3). RNA labeling was also confirmed by fluorescence microscopy after CuAAC reaction with Cy3-azide, with predominant 5-EC signal in nucleoli, the site of rRNA synthesis (Fig. 1e).

Next, we evaluated the crosslinking abilities of 5-EC with the catalytic domain of TET1 (TET1-CD). TET proteins are primarily DNA 5-methylcytosine oxidative demethylases, but they can also react with RNA^25,26^. For example, the single TET ortholog in *Drosophila* (dTet) is responsible for hm^5^C deposition in polyadenylated RNAs^16^. Moreover, TET1-CD can convert m^5^C to hm^5^C in RNA in vitro, and the catalytic domains of all three TET proteins, as well as full-length TET3, can induce hm^5^C formation in human RNA^15^. Gratifyingly, in cells treated with 5-EC and expressing TET1-CD, we observed the presence of a slower migrating band consistent with RNA-protein crosslinking (Supplementary Fig. 5a). This band was absent in untreated cells expressing TET1-CD and in 5-EC-treated samples expressing a catalytically inactive mutant of TET1-CD^27^, consistent with mechanism-dependent crosslinking. In addition, we recovered TET1-CD after 5-EC feeding and oligo(dT)-pulldown, but not the mutant version, or non-covalently bound TET1-CD in untreated cells (Supplementary Fig. 5b). Taken together, our results show that 5-EC is efficiently incorporated into RNA and can react with Fe(II)/α-KG-dependent dioxygenases in a mechanism-based manner in cells, thereby making it suitable for RNABPP^21^ analysis of m^5^C-modifying enzymes

### Proteomic profiling of 5-EC-reactive RNA modifying enzymes

**Fig. 2:**
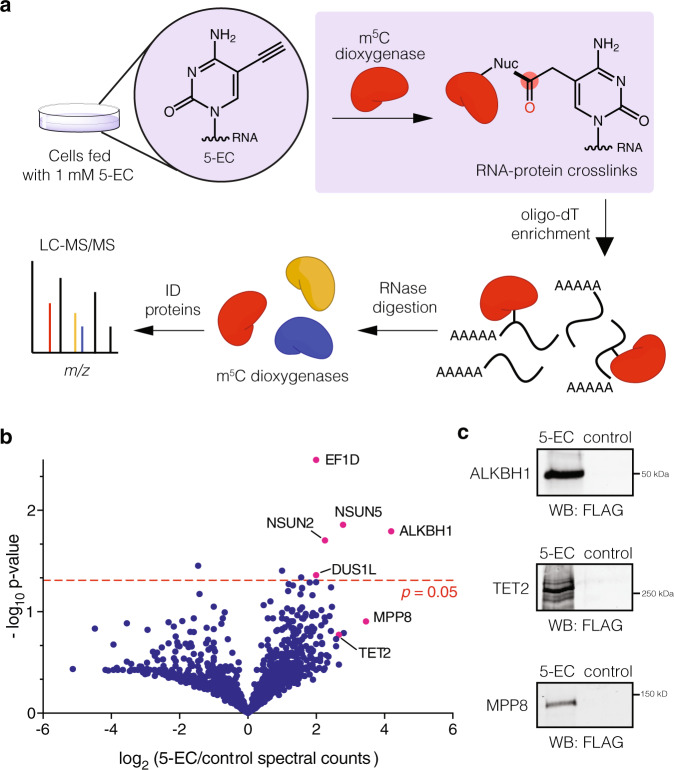
Proteomic analysis of m^5^C dioxygenases using 5-EC RNABPP. **a** 5-EC RNABPP workflow. Cells are metabolically labeled with 5-EC and crosslinked RNA-protein complexes are isolated by oligo(dT)-based enrichment. Enriched RNA-protein complexes are digested with RNase and then analyzed by mass spectrometry-based proteomics. **b** Volcano plot showing the enrichment of 5-EC reactive proteins. Experiment was performed independently in triplicate and protein abundance was quantified by spectral counting. Multiple student’s unpaired two-sided t-tests were used to assess statistical significance. **c** Western blot validation of ALKBH1, TET2, and MMP8. 293 Flp-In cells overexpressing the respective FLAG-tagged protein were treated with 1 mM 5-EC or DMSO and subjected to the RNABPP workflow before western blot analysis. Experiments were performed in triplicate with similar results. Source data are provided as a Source Data file.

After validation of 5-EC-mediated crosslinking with TET1-CD, we proceeded to investigate 5-EC-reactive proteins using our RNABPP workflow combining protein-RNA enrichment and quantitative mass spectrometry-based proteomics (Fig. 2a). We used comparative, label-free analysis to quantify protein enrichment in 5-EC treated cells versus untreated samples. After analysis of data from three independent biological replicates, we identified five proteins enriched by 5-EC treatment with statistically significant *P* values (<0.05). We were pleased to find that four are known or putative RNA pyrimidine modifying enzymes (Fig. 2b, Supplementary Data 1). Further, among the four pyrimidine modifying enzyme hits, the most highly enriched was ALKBH1, an Fe(II)/ α-KG-dependent dioxygenase responsible for oxidation of m^5^C to 5-formylcytosine (f^5^C) at the wobble position of mt-tRNA-Met^20^, and also reported to oxidize m^5^C at the wobble base of tRNA-Leu-CAA^19^. The activity of ALKBH1 on m^5^C sites in other RNA substrates is so far unknown. While ALKBH1 was not previously described to react with 5-EC, its identification using our method is consistent with its proposed catalytic mechanism^28^. We also identified m^5^C methyltransferases NSUN2 and NSUN5, and DUS1L, the mammalian homolog of yeast tRNA-dihydrouridine synthase (DUS) DUS1. NSUN-family m^5^C methyltransferases utilize a thymidylate synthase (TS)-like mechanism involving nucleophilic attack of a catalytic Cys residue on the C6 position of C to form an enzyme-bound intermediate, and a second Cys residue that mediates β-elimination after C5 methylation with SAM cofactor^29^. Interestingly, TS has been proposed to convert 5-ethynyl-2’-deoxyuridine into a thiol-reactive allene intermediate^30^. An analogous mechanism for NSUN proteins could lead to irreversible alkylation of the second Cys residue by 5-EC after rearrangement to its allene derivative (Supplementary Fig. 6). Reaction of 5-EU (generated by deamination of 5-EC in cells) with DUS1L may proceed similarly since C5-C6 reduction during dihydrouridine catalysis could also generate the proposed allene intermediate, and DUS enzymes contain a conserved catalytic Cys residue for irreversible alkylation^31^. Additionally, we observed enrichment, albeit below the *P* value threshold of 0.05, of TET2, which has been demonstrated to oxidize m^5^C to hm^5^C in RNA in vitro and in cells^12–16,32^, as well as MPP8, a chromatin-associated epigenetic reader protein that can interact with RNA^33^.

To validate the enriched proteins, we generated Flp-In 293 cell lines containing ALKBH1, TET2 or MPP8 and assessed their interaction with 5-EC labeled RNA using western blot and poly(A) pulldown. We initially focused on ALKBH1 and TET2 since they have been shown to oxidize m^5^C, and MPP8 since its biochemical activity and interaction with RNA has been poorly characterized. Gratifyingly, we observed recovery of all three proteins only in the presence of 5-EC using the RNABPP workflow (Fig. 2c, Supplementary Figs. 7, 8). For ALKBH1, we observed a slower migrating band upon 5-EC treatment, which we assign as the putative crosslinked RNA-protein species (Supplementary Fig. 7). We could not observe a similar band for TET2, likely due to its large size (224 kDa), which makes it challenging to resolve slower migrating crosslinked bands, or due to lower overall crosslinking efficiency. Similarly, we did not observe a crosslinked species for MPP8 (Supplementary Fig. 8), although RNABPP enrichment was specific for 5-EC over feeding with the related 5-methylcytidine nucleoside. Since this protein has no reported enzymatic activity or putative enzymatic domains, we did not pursue it further.

To understand the specificity of the ALKBH1 crosslinking, we compared the recovery of ALKBH1 against that of ALKBH5, a related Fe(II)/α-KG-dependent dioxygenase that can oxidatively demethylate N^6^-methyladenosine (m^6^A) in RNA^34^ but with no reported activity on m^5^C. We observed substantially greater recovery of ALKBH1 over ALKBH5 with 5-EC (Supplementary Fig. 9), indicating that efficient crosslinking to 5-EC is not a general property of Fe(II)/α-KG-dependent dioxygenase enzymes, and likely requires specific recognition of the C5 position on cytosine. Additionally, to assess if crosslinking is mechanism-dependent, we performed in vitro crosslinking assays with purified recombinant ALKBH1 and a chemically synthesized, site-specifically 5-EC-modified oligonucleotide substrate (oligo **1**) derived from the anticodon stem loop (ASL) of Mt-tRNA-Met (Supplementary Information, Supplementary Table 4). Consistent with our in-cell crosslinking results, we observed substantial amounts of a higher-running band when ALKBH1 was incubated with 5-EC oligonucleotide in the presence of required cofactors (i.e. α-KG, Fe(II), O_2_). (Supplementary Fig. 10). In contrast, we observed the absence of crosslinked species when the same ASL-derived oligonucleotide containing m^5^C (oligo **2**) instead of 5-EC was substituted in the reaction. Further, crosslinking was greatly reduced in reactions with 5-EC oligo **1** when α-KG, Fe(II), or RNA were omitted, as well as in reactions containing EDTA (iron chelator) or N-oxalyl glycine (NOG, an inhibitor of α-KG-dependent enzymes). Taken together, our data establish that 5-EC is an efficient mechanism-based probe for ALKBH1 that can be applied to crosslink RNA substrates in cells through metabolic labeling or for in vitro substrate analysis using chemically synthesized oligonucleotide probes.

### Contributions of ALKBH1 and TET2 to m^5^C oxidation

**Fig. 3:**
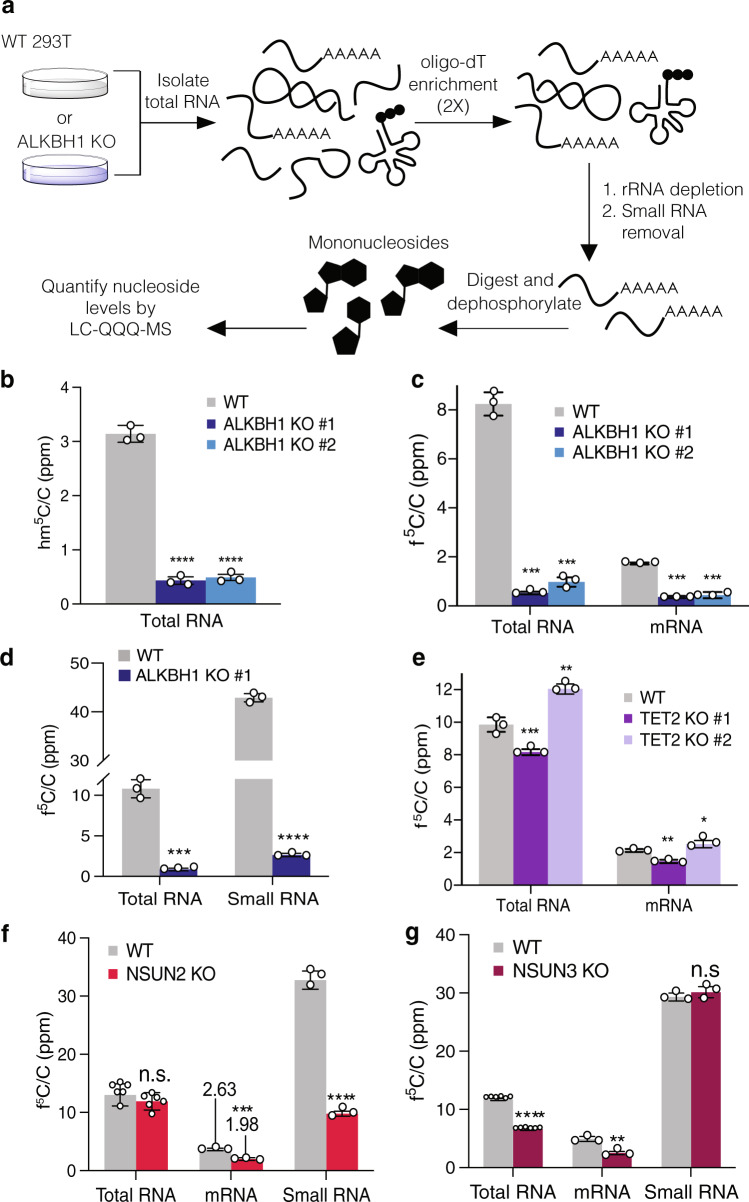
ALKBH1 is the major hm^5^C- and f^5^C-forming enzyme in mRNA and small RNA. **a** Strategy for mRNA isolation and LC-QQQ-MS quantification of modified nucleosides in HEK 293T wild-type (WT) and KO cells. Total RNA is isolated and subjected to two rounds of poly(A) enrichment, followed by rRNA and small RNA depletion. The mRNA is then digested to nucleosides and analyzed by LC-QQQ-MS. **b** Quantification of hm^5^C in total RNA from WT and ALKBH KO cell lines. p-values: WT vs. KO #1, *p* = 0.0000106; WT vs. KO #2, *p* = 0.0000103. **c** Quantification of f^5^C in total and mRNA from WT and ALKBH1 KO cell lines. *p*-values: WT vs. KO #1 in total RNA, 0.000004; in small RNA, 0.000001; WT vs. KO #2 in total RNA, 0.000007; in small RNA, 0.000036. **d** Quantification of f^5^C in small RNA from WT and ALKBH1 KO cells. *p*-values: WT vs KO in total RNA, *p* = 0.000109; in small RNA, *p* = 0.00000013. **e** Quantification of f^5^C in total and mRNA from WT and TET2 KO cell lines. *p*-values: WT vs. KO #1 in total RNA, 0.003594; in small RNA, 0.001040; WT vs. KO #2 in total RNA, 0.002192; in small RNA, 0.049386. **f** Quantification of f^5^C in total, mRNA, and small RNA from WT and NSUN2 KO cells. *p*-values: WT vs KO in total RNA, 0.285820; in mRNA, 0.00088; in small RNA; 0.000017. **g** Quantification of f^5^C in total, mRNA, and small RNA from WT and NSUN3 KO cells. p-values: WT vs KO in total RNA, 0.000007; in mRNA, 0.001198; in small RNA; 0.291189. Three independent biological replicates were analyzed, except in **f** and **g**, where *n* = 6 in total RNA measurements. Data represent mean values ± s.d. An unpaired *t* test (two-tailed) was used to measure the statistical significance *p  <  0.05, **p < 0.01, ***p < 0.001, ****p < 0.0001. Source data are provided as a Source Data file.

After validating interactions between ALKBH1 and TET2 with 5-EC containing RNA, we next studied the contributions of these proteins to m^5^C oxidation in cells. We prepared KO cells for each protein using CRISPR/Cas9 to target the corresponding gene in 293T cells (Supplementary Figs. 11–13, Supplementary Table 5). We then measured levels of m^5^C and reported oxidized products, hm^5^C, f^5^C, and 5-carboxycytidine (ca^5^C) in RNA isolated from both wild-type (WT) and KO cells by quantitative nucleoside LC-QQQ-MS (Supplementary Fig. 14, Supplementary Tables 6–10). We fractionated total RNA into small RNA and poly(A)-enriched RNA as described previously^21^ (Fig. 3a, Supplementary Figs. 15–18). Rigorous depletion of small RNA (which harbors high levels of many modified nucleotides) was confirmed by monitoring levels of N^6^-isopentenyladenosine levels (i^6^A), which is common to tRNA (Supplementary Fig. 19)^4^. We also ensured that contaminating nucleosides present in enzymatic reagents used for RNA digestion and dephosphorylation were at negligible levels (Supplementary Fig. 20).

We were able to detect low levels (ppm range) of both hm^5^C and f^5^C in WT total RNA (Fig. 3b–g, Supplementary Fig. 21, Supplementary Tables 6–9). We could not reliably detect ca^5^C in total or small RNA samples (Supplementary Fig. 22, Supplementary Table 10), therefore we did not focus on this modification for further investigation. Upon ALKHB1 depletion in two independent KO cell lines, hm^5^C levels dropped by 84–86% in total RNA (Fig. 3b) and 87–90% in small RNA (Supplementary Fig. 21). We were not able to reliably detect hm^5^C above background in mRNA. Consistent with previous reports^12,14^, we measured f^5^C levels of ~10 ppm f^5^C/C in total RNA from WT HEK293T cells (Fig. 3c–g). In the mRNA fraction, we measured f^5^C/C levels of 1.7 ± 0.03 ppm, ~5-fold less than in total RNA (Fig. 3c). In contrast, f^5^C/C levels in small RNA (primarily composed of tRNA) were increased ~4-fold relative to total RNA to 42.9 ± 0.9 ppm (Fig. 3d). Next, we measured f^5^C levels in the two independent ALKBH1 KOs. In all cases, we found a major reduction of f^5^C generation upon knockout of ALKBH1: 88–94% reduction in total RNA, 94% decrease in small RNA, and 74–79% reduction in mRNA (Fig. 3c, d). Taken together, our analysis of oxidative m^5^C modification levels in bulk RNA fractions shows that ALKBH1 is the major f^5^C- and hm^5^C-forming enzyme in HEK293T cells. Further, our data suggest that ALKBH1 can install f^5^C on polyadenylated RNA. Notably, while bulk poly(A)RNA fractions can be contaminated with modified nucleosides from more abundant RNA species^35^, given the low levels of f^5^C in total RNA and the purity of our poly(A) fraction (Supplementary Fig. 19), we believe that the observed modification levels are unlikely to have originated from non-poly(A)RNAs. Interestingly, we find that relative levels of f^5^C are highest in small RNA. This is consistent with reported ALKBH1-dependent f^5^C sites in mt-tRNA-Met and tRNA-Leu-CAA^19,20^, but could also indicate the existence of other ALKBH1-dependent f^5^C sites on tRNA.

Subsequently, we measured the contributions of TET2 to f^5^C and hm^5^C formation. We found no statistically significant TET2-dependence of f^5^C levels (Fig. 3e). TET2 has been shown to oxidize m^5^C to hm^5^C in cellular RNA^14–17,32,36^, and indeed we observed a 33–36% decrease in hm^5^C levels in small RNA and a 43–74% decrease in total RNA upon TET2 KO; hm^5^C levels in total RNA exhibited larger variability across multiple experiments, in part due to low abundance and challenges with chromatography (Supplementary Fig. 21, Supplementary Tables 8, 9). Together, our data indicate that TET2 and ALKBH1 generate hm^5^C in human cells, whereas only ALKBH1 is responsible for f^5^C formation.

### NSUN2- and NSUN3-dependent hm^5^C and f^5^C formation

The biosynthesis of hm^5^C and f^5^C on tRNA has been shown to involve the activity of at least two distinct enzymatic reactions—methylation of C to m^5^C, followed by oxidation of m^5^C^20^. Therefore, in order to investigate hm^5^C/f^5^C sites further, we analyzed their levels in HEK293T cells depleted of the RNA m^5^C methyltransferase NSUN2^21^ or NSUN3 (Supplementary Tables 11–14). NSUN2 is the major m^5^C methyltransferase in human cells and is responsible for installing m^5^C in both mRNA^37^ and tRNA^38,39^, including methylation of C34 on tRNA-Leu-CAA, which is further oxidized by ALKBH1^19^. NSUN3 is responsible for methylation of C34 on mt-tRNA-Met, which is converted to f^5^C by ALKBH1;^20,40^ no additional NSUN3-dependent m^5^C sites have been reported. Consistent with these reports^9,21,38,39^, we observed an 80–90% drop in m^5^C levels across total RNA, small RNA, and mRNA fractions from NSUN2 KO cells (Supplementary Fig. 23 Supplementary Tables 11, 12). We did not see any statistically significant decrease in m^5^C levels in NSUN3 KO cells suggesting a small number of substrate sites (Supplementary Fig. 23, Supplementary Tables 13, 14). In the NSUN2 KO, we observed a small but insignificant drop in f^5^C levels in total RNA, but a 45% decrease in f^5^C on poly(A) RNA and a 70% reduction in small RNA (Fig. 3f, Supplementary Tables 11, 12), indicating that most f^5^C sites on these substrates require NSUN2 methylation. For hm^5^C levels, we observed a 49–56% decrease in total RNA and 83–85% decrease on small RNA in the NSUN2 KO cells (Supplementary Fig. 21), demonstrating that the majority of hm^5^C oxidation occurs on NSUN2-installed m^5^C sites. We observed a different trend on NSUN3-dependent m^5^C sites: 46% decrease of f^5^C in total RNA and 50% decrease in mRNA upon NSUN3 KO (Fig. 3g, Supplementary Tables 13, 14), and only modest decreases in hm^5^C levels (Supplementary Fig. 21). Surprisingly, we detected no significant depletion in f^5^C levels upon NSUN3 KO in the small RNA fraction (Fig. 3g). While NSUN3 is required for f^5^C formation on mt-tRNA-Met^20,40^, our results indicate that this f^5^C site does not constitute a major fraction of f^5^C on small RNA, which is consistent with the relatively low abundance of mitochondrial tRNA species^41^, but also suggests the existence of additional NSUN3-independent f^5^C sites on small RNA. Taken together, our findings show that the majority of f^5^C sites on large RNA (>200 nt) are generated by ALKBH1-oxidation of NSUN3-dependent m^5^C residues, while the majority of f^5^C sites on small RNA originate from ALKBH1-oxidation of NSUN2-dependent m^5^C residues. Additionally, hm^5^C sites are generated primarily by ALKBH1-oxidation of NSUN2-dependent m^5^C residues. Further, we identify ALKBH1-dependent f^5^C sites on poly(A)RNA, broadening our understanding of the substrates of this enzyme and the distribution of f^5^C.

### ALKBH1 regulates m^1^A levels on small RNA

Liu et al.^42^ previously reported that ALKBH1 can remove m^1^A on tRNA through oxidative demethylation and regulate translation. Therefore, we also investigated the role of ALKBH1 in cellular m^1^A levels by nucleoside LC-MS. While we observed no significant change in total m^1^A levels upon ALKBH1 knockout, m^1^A levels in small RNA increased by 68 ± 0.09% (Supplementary Fig. 24A, Supplementary Table 15), consistent with ALKBH1-mediated demethylation on a variety of tRNA substrates^42^. We also interrogated whether ALKBH1 could affect m^1^A levels in response to stress, as reported by a recent study^43^. Again, we observed that total m^1^A levels are not dynamic and are insensitive to a variety of stresses including oxidative stress, heat shock, and glucose deprivation (Supplementary Fig. 24b, Supplementary Table 15). However, m^1^A levels on small RNA decreased by 37–53% in WT cells under all 3 stress conditions, but were unaffected in ALKBH1 KO cells, suggesting that stress activates ALKBH1 demethylation activity towards m^1^A on tRNA, in line with the findings of Niizuma and co-workers^43^. Interestingly, we did not observe changes in overall tRNA levels upon ALKBH1 KO or stress as measured by northern blot, indicating that m^1^A levels are not correlated with tRNA stability (Supplementary Fig. 24c–f). Finally, bulk f^5^C levels on total RNA or small RNA fractions did not change upon stress induction (Supplementary Fig. 25, Supplementary Table 15), suggesting that stress-induced ALKBH1 activity is specific to certain substrates.

### Mapping ALKBH1 substrates—5-EC-iCLIP

**Fig. 4:**
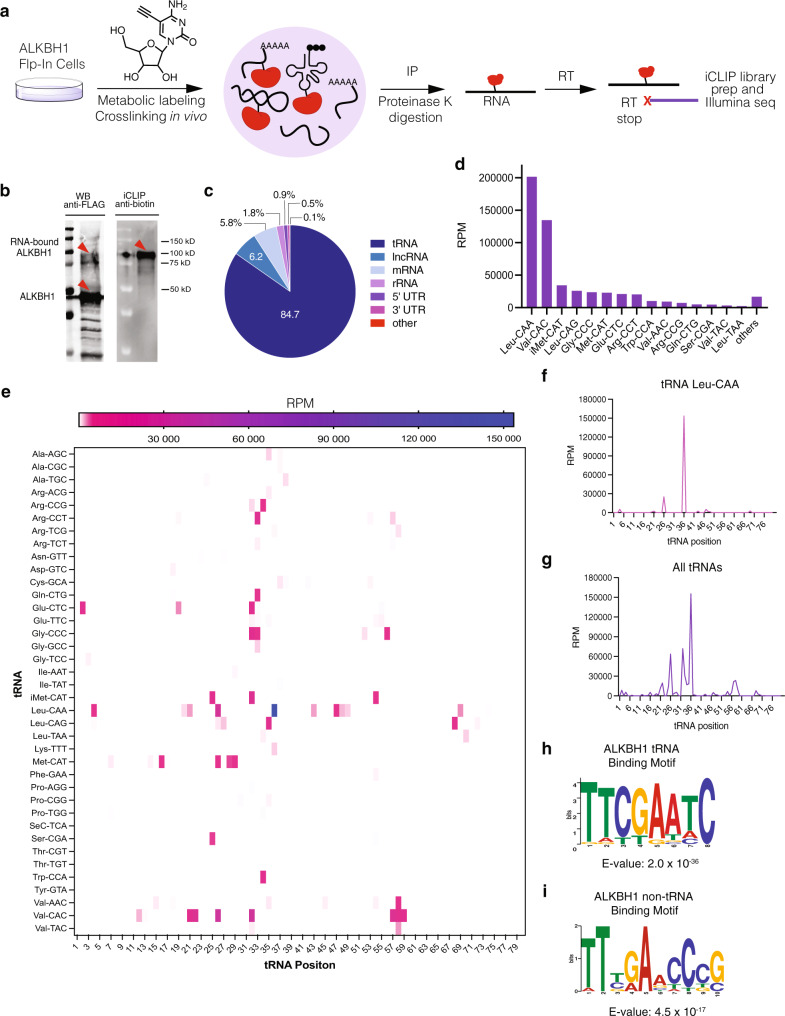
5-EC-iCLIP sequencing of ALKBH1 m^5^C RNA substrates. **a** Strategy for 5-EC-iCLIP workflow. Cells overexpressing ALKBH1 are metabolically labeled with 5-EC and the RNA-crosslinked protein is immunoprecipitated (IP) and digested. RNA is reverse transcribed (RT) and prepared for Illumina sequencing. **b** Analysis of RNA-ALKBH1 crosslinking in immunoprecipitated 5-EC-iCLIP samples after biotin labeling of RNA. Experiment was repeated two times with similar results. **c** Distribution of uniquely mapped reads identified by 5-EC-iCLIP; lncRNA, long non-coding RNA. **d** Abundance of tRNA species enriched by 5-EC-iCLIP. cDNA scores from reads were normalized to reads per million (RPM). **e** Heatmap showing enrichment (in RPM) of all crosslink peaks in tRNA targets according to their relative position in the mature tRNAs. Peaks below 5000 RPM are not visible on color scale. **f** Distribution of all peaks in tRNA Leu-CAA according to their relative position in the mature tRNA. **g** Distribution of crosslink peaks in all tRNA targets identified by 5-EC-iCLIP according to their relative position in the mature tRNAs. **h** tRNA consensus motif identified by MEME from all tRNA peaks identified by 5-EC-iCLIP. **i** non-tRNA consensus motif identified by MEME from iCLIP non-tRNA peaks.

Next, we investigated RNA substrates of ALKBH1 by adapting the iCLIP sequencing method (Fig. 4a)^44^. We metabolically labeled RNA in cells overexpressing FLAG-tagged ALKBH1 with 5-EC and immunoprecipitated crosslinked ALKBH1-RNA complexes. After IP, anti-FLAG and streptavidin Western blot (following enzymatic biotinylation of associated RNA) indicated robust protein-RNA crosslinking (Fig. 4b, Supplementary Fig. 26). Crosslinked RNA was then fragmented, reverse transcribed, and prepared for Illumina sequencing following literature precedent (Supplementary Fig. 27)^21,39,45,46^. After sequencing, we performed bioinformatic analysis using the iMaps platform. Reads were de-duplicated and aligned to the genomic reference, and sites of crosslinking were identified based on reverse transcription (RT) stop signatures (Fig. 4a). Crosslinking sites were aggregated into peaks via Paraclu^46^, and only peaks with all of the following criteria were considered for further analysis: (a) present in two independent biological replicates, (b) displaying a cDNA score >10 and (c) absent from the no 5-EC control OR present in the non-5EC control but with a 5-EC/control score ratio >1.5 (Supplementary Data 2).

After removing intergenic reads, we observed the largest fraction of unique reads (84.7%) corresponding to cytosolic tRNAs, indicating that tRNA is the major substrate of ALKBH1 (Fig. 4c). In contrast, the read distribution in the control (no 5-EC treatment) resembles the normal RNA distribution in cells, with rRNA constituting 46.7% of the reads, followed by tRNA (15.3%) (Supplementary Fig. 28). He and co-workers reached a similar conclusion using UV-based CLIP analysis^42^. Among the 38 tRNA isodecoder families identified in our 5-EC-iCLIP experiment, cytosolic tRNA-Leu-CAA, which is known to contain ALKBH1-dependent f^5^C/f^5^Cm and hm^5^C/hm^5^Cm modifications at the wobble position, was the most abundant, encompassing 37% of the tRNA reads (Fig. 4d, e), and we observed the most abundant crosslinking peak in our iCLIP data at position 36 of tRNA-Leu-CAA (Fig. 4e, f, Supplementary Data 2). While the wobble base in this tRNA is located at residue 35, crosslinking peaks can be offset by one or two nucleotides depending on where RT stop occurs^47^. The presence of a crosslinking peak in our iCLIP experiment corresponding to a known ALKBH1-dependent m^5^C oxidation site validates our method and establishes it as an effective tool to map substrates of ALKBH1-mediated m^5^C oxidation.

In addition to a peak at the wobble position of tRNA-Leu-CAA, we identified 165 peaks in cytosolic tRNA (Supplementary Data 2). To visualize tRNA crosslinking, we aligned all peaks according to their relative position on the mature transcript and observed the largest accumulation of crosslinking at positions 32–36 (54.0% of all peaks) (Fig. 4e, g). 25 out of the 38 tRNA isodecoders identified displayed peaks between positions 32–36. Depending on tRNA length, positions 32–36 align with the anticodon, suggesting ALKBH1 can oxidize m^5^C in the anticodon or anticodon stem loop (ASL) of a number of cytosolic tRNAs. Consistent with crosslinking requiring 5-EC labeling, 21 of the identified tRNAs with crosslinks in this region have a C residue in the anticodon loop. We also observed 8 tRNAs with crosslinking peaks at positions 56–59 (11.1% of all peaks), which is likely due to the presence of a highly conserved m^1^A residue in the TψC -loop^48,49^. Rather than crosslinking to ALKBH1, this modification can induce an RT stop on its own^50^, thus generating an apparent crosslinking peak at that position. Another possibility is that the incorporation of 5-EC near this m^1^A residue could mediate crosslinking, particularly given the conservation of C at position 56 in many tRNAs. There was also an additional cluster of 10 tRNAs that displayed peaks at positions 25–28 (15.6% of all peaks). In addition to 5-EC-mediated crosslinking in this region, RT stops could be induced by the presence of a conserved N^2^,N^2^-dimethylguanosine (m^2^_2_G) modification in this region^4,51^. Sequence motif analysis using multiple expectation maximizations for motif elicitation (MEME)^52^ revealed a tRNA consensus motif that matches the TψC-loop of various isoacceptors, likely due to peaks near m^1^A58 (Fig. 4h). A consensus motif matching the anticodon loop was not obtained, as these sequences diverge among tRNAs.

Since we initially identified ALKBH1 as an m^5^C dioxygenase using oligo(dT)-based enrichment and were able to quantify ALKBH1-dependent f^5^C in mRNA, we also analyzed CLIP peaks mapping to non-tRNA regions. We found a total of 83 peaks, with 70.0% mapping to introns of protein-coding genes (Supplementary Data 2). The remaining reads mapped to non-coding RNAs (17.1%), 5’ UTR (11.5%), 3’ UTR (0.82%), and coding regions (0.55%) (Supplementary Fig. 29). On average, peaks observed for non-tRNA were ~40-fold lower in abundance than tRNA peaks. We also performed MEME analysis on the non-tRNA reads and identified the sequence binding logo TTBRADCCCG (Fig. 4i), which shares similarities to the tRNA-binding consensus sequence (Fig. 4h).

### Mapping ALKBH1 substrates—ARP sequencing

**Fig. 5:**
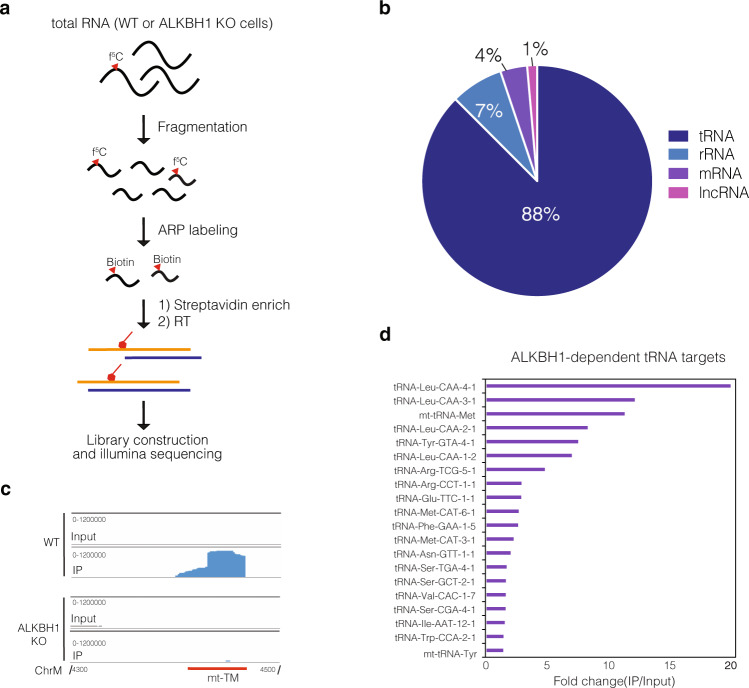
Transcriptome-wide profiling of RNA f^5^C using Aldehyde-reactive probe (ARP)-sequencing. **a** Workflow for ARP sequencing. Fragmented total RNA extracted from WT and ALKBH1 KO cells is incubated with ARP, and labeled RNA is enriched through streptavidin pulldown. Libraries are generated through RT, cDNA circulation, and PCR amplification. **b** Distribution of enriched ALKBH1-dependent peaks (cDNA value > 10) identified by ARP sequencing. **c** IGV tra**c**ks showing the reads mapped to mt-tRNA-Met in input and IP samples of WT and ALKBH1 KO cells. **d** Top ALKBH1-dependent f^5^C-containing tRNA substrates enriched by ARP pulldown. Only peaks with cDNA score >100 and present in the WT sample and absent from the ALKBH1 KO control were considered.

Identification of f^5^C in DNA has been previously achieved by pulldown with aldehyde-reactive probe (ARP; O-(biotinylcarbazoylmethyl) hydroxylamine)^53^. Inspired by this method, we reasoned that we could also map f^5^C sites within RNA by ARP-based pulldown followed by NGS (Fig. 5a). Fragmented total RNA extracted from WT or ALKBH1 KO cells was first incubated with ARP and then enriched by streptavidin pulldown followed by on-bead RT and Illumina library construction. A total RNA input library was also prepared and was used as a background control for the identification of read-enriched regions (i.e. peaks) in the pulldowns. Pulldown efficiency was ensured by adding an f^5^C-containing spike-in oligo prior to the IP step, which was enriched 20–40-fold in both WT and KO samples across two biological replicates (Supplementary Fig. 30a), suggesting the workflow can efficiently enrich f^5^C-containing RNA.

To define ALKBH-dependent f^5^C targets, we analyzed the transcriptome-wide ARP data considering only peaks with cDNA score >10 present in WT pulldown samples, and absent from the input and ALKBH1 KO controls (Supplementary Data 3). In line with the 5EC-iCLIP data, we found a majority of unique reads (88%) mapped to tRNA (Fig. 5b), with known f^5^C-containing substrates mt-tRNA-Met and cytosolic tRNA-Leu-CAA constituting the majority of the tRNA reads (Fig. 5c, Supplementary Data 3). Aside from these 2 targets, we also identified 32 peaks in other cytosolic tRNAs with fold-change >1.5, including 20 tRNAs with cDNA value >100 (Fig. 5d) which we considered as high-confidence f^5^C-containing targets. Some of these, namely tRNAs Arg-TTC, Met-CAT, Val-CAC, Ser-CGA, and Trp-CCA were also present as top enriched hits in our 5-EC-iCLIP experiment (Fig. 4d, Supplementary Fig. 30b), validating our iCLIP findings and strengthening the notion that isoacceptors other than mt-tRNA-Met and tRNA-Leu-CAA also contain f^5^C. We also found that 4% of total enriched peaks mapped to mRNA, suggesting again the presence of ALKBH1-dependent f^5^C on mRNA (Supplementary Data 3).

### Measuring hm^5^C/f^5^C content in native tRNAs

**Fig. 6:**
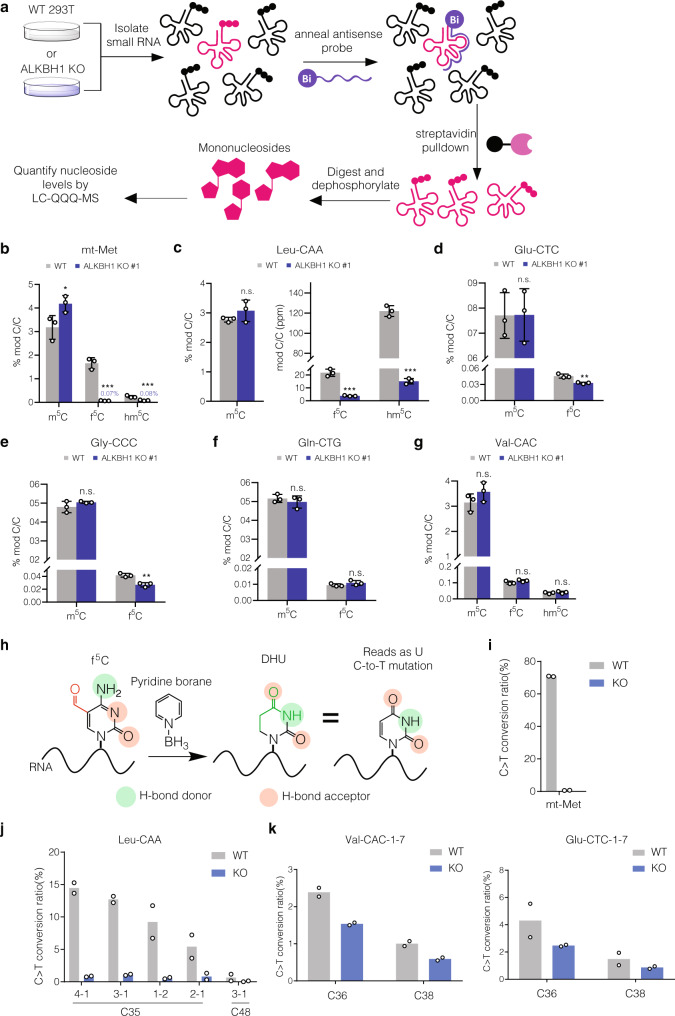
Validation of 5-EC-iCLIP tRNA hits. **a** Strategy for LC-QQQ-MS quantification of modified nucleosides in individual tRNAs isolated from HEK 293T wild-type (WT) and ALKBH1 KO cells. Individual tRNAs are enriched by antisense pulldown, digested to mononucleosides and analyzed by LC-QQQ-MS. **b**–**g** Quantification of oxidized m^5^C products in **b** mt-tRNA-Met, p-values: WT vs KO for m^5^C, 0.026079; for f^5^C, 0.000132; for hm^5^C, 0.000244, **c** tRNA-Leu-CAA, *p*-values: WT vs KO for m^5^C, 0.156295; for f^5^C, 0.000170; for hm^5^C, 0.000002, **d** tRNA-Glu-CTC, *p*-values: WT vs KO for m^5^C, 0.975934; for f^5^C, 0.002584, **e** tRNA-Gly-CCC, *p*-values: WT vs KO for m^5^C, 0.161660; for f^5^C, 0.001039, **f** tRNA-Gln-CTG, p-values: WT vs KO for m^5^C, 0.365015; for f^5^C, 0.171454, and **g** tRNA-Val-CAC, p-values: WT vs KO for m^5^C, 0.152377; for f^5^C, 0.124395; for hm^5^C, 0.366551. **h** Schematic workflow for pyridine borane f^5^C sequencing. Pyridine borane converts f^5^C residues to dihydrouridine, which is read as uridine, thus generating a C-to-T signature that can be identified in sequencing (**i**) Validation of presence and ALKBH1-dependence of f^5^C at the wobble base of mt-tRNA-Met by pyridine borane sequencing. **j** Validation of presence and ALKBH1-dependence of f^5^C at the wobble base of tRNA-Leu-CAA. **k** Presence and ALKBH1-dependence of f^5^C at various positions of tRNA-Val-CAC and tRNA Glu-CTC. For (**b**)–(**g**), three independent biological replicates were analyzed. Data represent mean values ± s.d. An unpaired t-test (two-tailed) was used to measure the statistical significance *p  <  0.05, **p < 0.01, ***p < 0.001, ****p < 0.0001. For (**h**)–(**j**), two independent biological replicates were performed and analyzed. Source data are provided as a Source Data file.

To interrogate potential ALKBH1-dependent f^5^C sites in tRNAs we identified through our sequencing studies, we performed antisense pulldowns^42^ on RNA isolated from WT or ALKBH1 KO cells followed by quantitative LC-QQQ-MS to measure m^5^C and its oxidized derivatives (Fig. 6a, Supplementary Figs. 31–33, Supplementary Tables 16, 17). We first analyzed mt-tRNA-Met and tRNA-Leu-CAA. We were pleased to measure hm^5^C and f^5^C levels in both tRNAs that decreased significantly in ALKBH1 KO cells (Fig. 6b, c). For mt-tRNA-Met, we observed a 96% decrease in f^5^C, from 1.6% to 0.07%. We also measured hm^5^C in this tRNA, which was reduced 65% upon ALKBH1 depletion (albeit present at much lower abundance than f^5^C). In contrast, tRNA-Leu-CAA showed higher hm^5^C levels compared to f^5^C, consistent with previously reported oligonucleotide mass spectrometry analysis^19^. We further applied this approach to four other tRNAs from our 5-EC-iCLIP data – tRNA-Glu-CTC, tRNA-Gly-CCC, tRNA-Gln-CTG, and tRNA-Val-CAC. We were able to detect f^5^C but not hm^5^C in all substrates. For both tRNA-Glu-CTC (Fig. 6d) and tRNA-Gly-CCC (Fig. 6e) we measured modest but statistically significant decreases in f^5^C upon ALKBH1 KO (38% reduction for tRNA-Glu-CTC and 33% reduction for tRNA-Gly-CCC). Interestingly, f^5^C levels in tRNA-Val-CAC and tRNA-Gln-CTG were unchanged upon ALKBH1 knockout (Fig. 6f, g).

Since LC-MS analysis of bulk RNA digests does not provide information on the modification site and antisense pulldowns could still contain trace amounts of non-targeted tRNA species, we decided to pursue a complementary sequencing approach to more rigorously characterize tRNA f^5^C sites. In particular, Song and co-workers demonstrated that 5fC in DNA can be converted to dihydrouracil by pyridine borane treatment, resulting in C-to-T mutations upon PCR and sequencing^54^, and while our manuscript was in this review, this method was adapted to identify RNA f^5^C sites in yeast^55^. Similarly, we previously reported Mal-Seq^56^, which uses malononitrile to detect f^5^C in RNA through C-to-T mutations, although with lower sensitivity than the pyridine borane procedure. Therefore, we employed pyridine borane sequencing together with targeted RT-PCR analysis to study f^5^C sites on tRNAs of interest (Fig. 6h). We designed primers to sequence bases 19 to 50, which includes the anticodon stem loop (ASL), since our CLIP data indicated that 5-EC-induced crosslinking peaks frequently occurred in this region First, we investigated whether pyridine borane would effectively generate C-to-T mutations at f^5^C34 in mt-tRNA-Met, which is reported to exist at near 100% stoichiometry^40^. While f^5^C34 is sequenced almost exclusively as “C” from untreated RNA, RT-PCR on pyridine borane-treated RNA showed 71% C-to-T mutation (Fig. 6i). Further, we detected no mutations at C34 in mt-tRNA-Met in pyridine borane-treated RNA isolated from ALKBH1 KO cells, indicating that pyridine borane sequencing can specifically detect f^5^C in RNA (Supplementary Data 4).

Next, we chose 7 additional tRNA isoacceptors from our 5-EC-iCLIP data to analyze by pyridine-borane RT-PCR (Supplementary Data 4). In order to identify C-to-T conversion events likely to originate from f^5^C modification, we compared pyridine borane-induced mutations in WT and ALKBH1 KO RNA. In total, among the 8 tRNAs investigated, we identified 15 sites showing pyridine borane-induced mutations in WT RNA and lacking mutations in ALKBH1 KO RNA (Supplementary Data 4). Consistent with our CLIP data and ARP sequencing data, 4 of these sites mapped to the wobble base (C35) in tRNA-Leu-CAA isodecoders and showed mutation rates of 6–15% that were greatly reduced in the ALKBH1 KO (Fig. 6j). We should note that tRNA-Leu-CAA has been reported to contain both f^5^C and its 2’-O-methyl derivative f^5^Cm^19^, and we predict that pyridine borane-induced C-to-T mutations should result from both modifications. Further, since we detected only 71% C-to-T mutation at f^5^C34 in mt-tRNA-Met (which is reportedly modified at near 100% stoichiometry)^40^, the pyridine borane-induced mutation rate is likely to be an underestimate of actual modification stoichiometry. In addition, we detected ALKBH1-dependent mutations in the anticodon of tRNA-Gly-CCC, tRNA-Val-CAC and Glu-CTC at a frequency of 0.95–4.3% (Fig. 6k, Supplementary Data 4). Mutations surrounding the anticodon in the anticodon stem loop were also found in tRNA-Val-CAC, tRNA-Met-CAT, tRNA-Glu-CTC, and tRNA-Gly-CCC (Fig. 6j, k, Supplementary Data 4). Finally, we detected a small number of ALKBH1-independent f^5^C sites (i.e. with comparable rate of C-to-T mutation in both WT and ALKBH1 KO samples but sensitive to pyridine borane treatment) at position 25 of tRNA-Val-CAC and position 39 of tRNA-Gly-CCC, which require further investigation (Supplementary Fig. 34, Supplementary Data 4). Taken together, our data indicate the existence of f^5^C sites occurring in several cytosolic tRNAs.

### Assessment of in vitro ALKBH1 substrate specificity

**Fig. 7:**
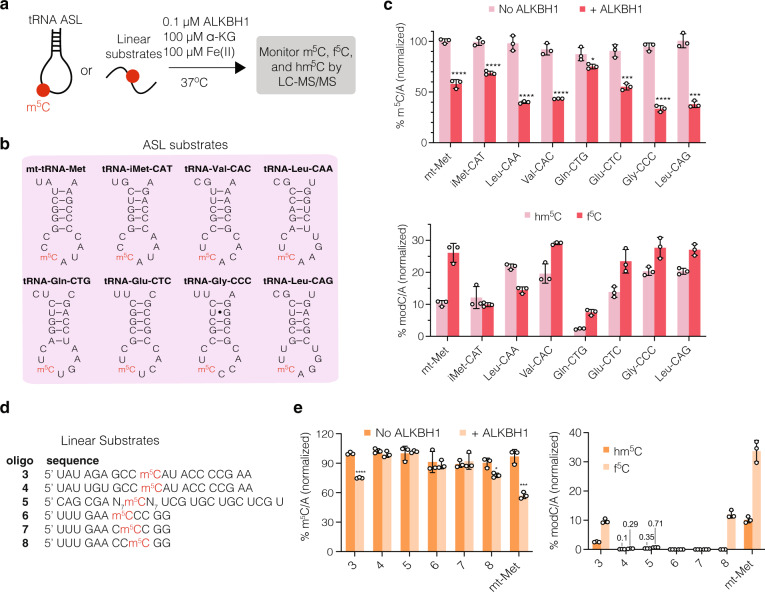
In vitro activity of ALKBH1 on structured and linear substrates. **a** Workflow to assess activity of ALKBH1 in vitro. Purified ALKBH1 is incubated with RNA substrate and cofactors at 37 °C for 30 min. The reaction is quenched, and RNA is purified, digested, and analyzed by LC-QQQ-MS. Activity was measured by quantifying the decrease of m^5^C and the generation of oxidized products hm^5^C and f^5^C. **b** Sequences of anticodon stem loops (ASLs) tested in the assay. All substrates have an m^5^C residue at the wobble base. **c** Conversion of m^5^C and generation of hm^5^C and f^5^C in various ASL substrates. **d** Sequences of linear substrates tested in the assay. **e** Conversion of m^5^C and generation of hm^5^C and f^5^C in linear substrates. For (**c**) and **e**, to account for loading differences, values were first quantified as ratios over A and then normalized to the maximum possible ratio for that substrate. Three independent replicates were performed, and data represent mean values ± s.d. An unpaired t-test (two-tailed) was used to measure the statistical significance *p  <  0.05, **p < 0.01, ***p < 0.001, ****p < 0.0001. For (**c**), *p*-values (+ALKBH1 versus No enzyme) for substrate 3, 0.000006; for substrate 8, 0.0141996; for mt-Met, 0.0009131. For (**e**), *P*-values (f^5^C versus hm^5^c) for substrate 3, 0.0000927; substrate 4, 0.009919; for substrate 5, 0.0000889; for substrate 8, 0.000914; for mt-Met, 0.0004327. Source data are provided as a Source Data file.

To better understand 5-EC-mediated crosslinking on diverse substrates as detected in our iCLIP experiment, we explored the in vitro activity of recombinant ALKBH1 on m^5^C-containing RNA oligonucleotides (Fig. 7a). We synthesized the ASLs from tRNAs Leu-CAA, iMet-CAT, Val-CAC, Gln-CTG, Glu-CTC, Gly-CCC, and Leu-CAG with m^5^C at the wobble position (Fig. 7b, Supplementary Table 4), given that those tRNAs showed both high levels of enrichment in the CLIP data and crosslinks near the wobble base. As a positive control, we prepared the m^5^C-modified ASL from mt-tRNA-Met, which has been reported to serve as an in vitro substrate for ALKBH1 oxidation^20^. We then setup in vitro enzyme reactions containing purified ALKBH1 with necessary cofactors and RNA substrate, and analyzed the reaction products by LC-QQQ-MS (Fig. 7c, Supplementary Fig. 35, Supplementary Tables 18–21). After incubation with ALKBH1, all synthetic m^5^C-containing ASL substrates showed statistically significant decreases in m^5^C/A levels, ranging from 12–60%, compared to the no-enzyme control. A concomitant increase in f^5^C/A and hm^5^C/A levels was also observed. Consistent with our 5-EC-iCLIP data, the most active ASLs substrates were Leu-CAA, Val-CAC, Gly-CCC, and Leu-CAG, which showed between 49–60% decrease in normalized m^5^C levels compared to 42% depletion for the mt-tRNA-Met ASL. Production of f^5^C in these tRNAs (27–29% normalized f^5^C) was also comparable to that in the mt-tRNA-Met control (26%), with the exception of tRNA-Leu-CAA, which showed f^5^C/A levels at 15%. However, tRNA-Leu-CAA displayed the highest hm^5^C content post-oxidation (21% normalized hm^5^C) among all the substates. These higher levels of hm^5^C compared to f^5^C observed in vitro are consistent with the levels of modified nucleosides measured from the native tRNA (Fig. 6c). Similar to our analysis of cellular RNA, 5-carboxycytidine (ca^5^C) was not reliably detected above background after ALKBH1-mediated oxidation in vitro (Supplementary Fig. 35, Supplementary Table 19), and we did not detect appreciable levels of contaminating nucleosides originating from recombinant ALKBH1 (Supplementary Fig. 20).

In addition, we measured the activity of ALKBH1 on linear substrates including mutated m^5^C-modified ASLs from mt-tRNA-Met (**3**–**4**), a randomized 15-mer RNA library containing a single m^5^C site in the center (**5**) (Supplementary Fig. 36, Supplementary Table 4), and m^5^C-modified oligonucleotides matching the non-tRNA consensus sequence detected from the 5-EC-iCLIP experiment (**6**–**8**) (Fig. 7d, Supplementary Tables 20–21). While none of these linear oligonucleotides showed the same degree of m^5^C oxidation as the ASL substrates, we were able to detect m^5^C depletion and hm^5^C/f^5^C formation on substrate **3**, one of the mutated mt-Met ASL substrates, as well as substrate **8**, corresponding to the non-tRNA iCLIP sequence logo (Fig. 7e). The m^5^C random library was also subject to minimal conversion (0.35% and 0.71% hm^5^C/C and f^5^C/C normalized formation, respectively), which suggests a slight preference of ALKBH1 for some linear substrates contained within. Taken together, our in vitro activity data shows that ALKBH1 can efficiently oxidize m^5^C-containing stem loops taken from diverse tRNAs. Further, in select sequence contexts, ALKBH1-mediated m^5^C oxidation can also occur on linear substrates.

### Investigating the potential function of f^5^C in tRNA Leu-CAA

Since our 5-EC-iCLIP data, ARP-sequencing analysis, LC-MS/MS analysis, and pyridine borane data all support the presence of f^5^C at the wobble position of tRNA Leu-CAA, we posited whether the modification could facilitate translation by decoding non-cognate TTA Leu codons, analogous to the proposed function of f^5^C on mt-tRNA-Met in decoding both AUA and AUG codons. We assessed the translation efficiency of TTG and TTA codons through an in-cell dual-luciferase assay in WT 293T or ALKBH1 KO cells. In this assay, cells are transfected with a construct containing Renilla luciferase (Rluc) linked to Firefly luciferase (Fluc). Efficient readthrough of the linker, which, in this case consists of a string of either TTG, TTA, or random codons can be quantified by an increase in Fluc signal as compared to the Rluc output. Under normal cell culture conditions, we observed no significant changes in fluorescence among different constructs or cell lines (Supplementary Fig. 37, Supplementary Table 22), indicating that translation of the dual-luciferase constructs was not affected by ALBKH1 KO. However, under glucose deprivation stress (1 mM glucose), the translation efficiency of the TTA codon linker decreased by 17.5% in ALKBH1 KO cells. In contrast, translation of the TTG linker or the random codon linker was unaffected in ALKBH1 KO cells compared to WT cells. Taken together, our data suggest that f^5^C at the wobble base of tRNA-Leu-CAA may aid in decoding Leu TTA codons under stress conditions.

## Discussion

In this study, we develop an activity-based platform to profile RNA m^5^C dioxygenases in human cells. Our approach relies upon 5-EC nucleoside, which is incorporated efficiently and broadly into the transcriptome through nucleotide metabolism, where it can form covalent crosslinks with enzymes that recognize and oxidize m^5^C in RNA. We find that ALKBH1 is the major m^5^C dioxygenase in human HEK293T cells, where it is responsible for the bulk of hm^5^C and f^5^C production. Further, we use quantitative nucleoside LC-MS, modification-specific sequencing approaches, and in vitro biochemical assays to study the substrate specificity of ALKBH1 and identify the presence of f^5^C sites across the human transcriptome. Taken together, our work expands our knowledge of oxidative m^5^C modifications on RNA and provides powerful strategies to further explore these modifications and associated writer enzymes in biology.

We chose 5-EC as a mechanism-based probe for RNA m^5^C dioxygenases based upon the ability of the analogous deoxynucleoside to crosslink TET in vitro and in lysate^23^. Precedent also exists for the covalent trapping of other oxidative enzymes with terminal alkyne moieties. Stubbe and coworkers showed that thymine hydroxylase is inactivated by 5-EU^57^, and aryl acetylene substrates have been used as activity-based probes for cytochrome P-450 enzymes^58,59^. While the mechanistic basis of crosslinking has not been fully elucidated for each of these systems, the formation of a covalent adduct is likely to occur through the production of a highly reactive ketene intermediate following oxidation and concomitant 1,2 migration of the acetylenic hydrogen^58^. The putative ketene can then react with nearby nucleophilic residues resulting in covalent trapping of the protein^60^. Our work shows that 5-EC is an effective surrogate for m^5^C and can be applied as a general metabolic probe to study RNA m^5^C dioxygenases in their native context. Interestingly, despite serving as a reactive m^5^C surrogate, we did not observe depressed levels of m^5^C (and its corresponding oxidized products) in 5-EC treated samples, which suggests that this probe does not significantly affect the cellular activities of m^5^C-forming or m^5^C-oxidizing enzymes and hints to its broad applicability in other settings. Analogous approaches with alkyne-containing metabolites should be readily applicable to study diverse oxidative enzymes acting upon biopolymer and small molecule substrates, as well as to study other proposed substrates of ALKBH1^59,61,62^.

Our work identifies new RNA m^5^C substrates for ALKBH1-mediated oxidation to f^5^C. Previous studies have reported two RNA m^5^C sites that are substrates for ALKBH1—m^5^C34 in mt-tRNA-Met (oxidized to f^5^C) and m^5^C(m)34 in cyt-tRNA-Leu-CAA (oxidized to hm^5^C/f^5^C and hm^5^Cm/f^5^Cm). Using nucleoside LC-MS analysis of fractionated RNA from WT, ALKBH1 KO, NSUN2 KO, and NSUN3 KO cells, we show that ALKBH1 installs f^5^C on the poly(A)RNA fraction through oxidation of NSUN2- and NSUN3-dependent m^5^C sites. The pathway for f^5^C formation on C34 of mt-tRNA-Met requires NSUN3^20,40^ and CLIP studies have proposed that this is the only substrate for the enzyme;^63^ however, we observe no NSUN3-dependent depletion in f^5^C levels on small RNA (which contains mt-tRNA-Met), and instead observe NSUN3-dependent depletion of f^5^C in total RNA and poly(A)RNA, further supporting the existence of f^5^C (and m^5^C) sites catalyzed by NSUN3 and ALKBH1 beyond the canonical mt-tRNA-Met substrate. One important consideration that must be accounted for in the interpretation of low abundance hm^5^C and f^5^C sites is the possibility that these are a result of catabolism of abundant hm^5^C/f^5^C-containing RNAs followed by non-specific incorporation during transcription. While an analogous chain of events has been proposed to explain the presence of 6-methyladenine on mammalian DNA^64^, we do not favor this scenario since hm^5^C and f^5^C are present at low overall abundance in the transcriptome (in contrast to m^6^A), and it is unknown whether they are efficiently processed by pyrimidine salvage pathways.

To map ALKBH1-dependent hm^5^C/f^5^C sites at nucleotide resolution, we developed and applied multiple complementary sequencing platforms. 5-EC-iCLIP analysis and ARP-sequencing profiled ALKBH1 m^5^C substrates transcriptome-wide and showed that its major substrate is tRNA, which is consistent with a previous UV-CLIP study for ALKBH1^42^, but we also found additional minor modification sites on intronic mRNA, UTR, lncRNA, and CDS regions. Indeed, the crosslinking peak at the wobble base of cyt-tRNA-Leu-CAA was the most abundant of all those identified and is likely the major ALKBH1 m^5^C substrate in addition to mt-tRNA-Met. The ability of ALKBH1 to act upon both nuclear/cytosolic and mitochondrial substrates is worthy of further exploration due to the primarily mitochondrial localization of the protein^65^. In addition, we find 5-EC-mediated crosslinking of ALKBH1 to 38 other tRNA isodecoder famlies, with the majority of peaks mapping to the ASL, and ARP-sequencing supports additional tRNA substrates beyond cyt-tRNA-Leu-CAA and mt-tRNA-Met. We interrogated the presence of f^5^C at these sites using targeted RT-PCR and pyridine borane-mediated f^5^C-to-D transformation (thereby producing characteristic C-to-U mutations upon RT)^54^. While not all 5-EC-iCLIP peaks in tRNA could be validated by pyridine borane sequencing, this may be due to the presence of hm^5^C (which does not react with pyridine borane), or due to crosslinking mediated by 5-EC incorporation at sites that lack m^5^C in the native system. In the latter case and supported by our comprehensive in vitro biochemical studies with recombinant ALKBH1, our analysis supports promiscuous activity of ALKBH1 on a variety of tRNA ASL sequences and even some linear m^5^C-containing sequences, suggesting that hm^5^C and f^5^C installation is likely to occur at these sites in systems where m^5^C deposition is upregulated.

We also identify TET2 as a 5-EC reactive protein, consistent with previous studies showing that TET2 converts RNA m^5^C in vivo and in vitro to hm^5^C^15,23^. Our data do not implicate TET2 in the formation of f^5^C in human total RNA, but do show that TET2 contributes to global hm^5^C formation (albeit to a lesser extent than ALKBH1), consistent with previous reports^17,18,66^. We propose that 5-EC metabolic labeling could be applied in diverse biological contexts to map the substrate of TET2 in an activity-dependent manner using 5-EC-iCLIP or analogous platforms.

Finally, our work motivates further investigation into the biological role of ALKBH1 and f^5^C. The presence of f^5^C at the wobble base of mt-tRNA-Met has been proposed to facilitate reading of unconventional mitochondrial Met codons^67^, and ALKBH1 KO leads to mitochondrial dysfunction^40,63^. The role of ALKBH1 in mediating cytoplasmic translation has been suggested to occur through demethylation of tRNA m^1^A58^42^; however, it is plausible that ALKBH1-dependent installation of f^5^C or related derivatives in the ASL of cytoplasmic tRNAs may also serve to tune decoding functions or to regulate tRNA structure and/or stability. Our initial studies of wobble f^5^C in tRNA Leu-CAA suggest a role for the modification in decoding non-cognate codons more efficiently under stress and could implicate f^5^C in the regulation of stress response, but more research is needed in this area. Additionally, f^5^C modifications in mRNA, if present at substantial stoichiometry, may modulate the expression of individual protein products. Continued work in this area will undoubtedly broaden our appreciation of epitranscriptomic processes and RNA biology.

## Methods

### Plasmid construction

Plasmids encoding cDNA were from the following sources: TET1 (Addgene #49792), TET1-CD H1762Y A1674A (Addgene #84479), TET2 (Addgene #41710), ALKBH1 (Genscript #OHu05179D), MPP8 (Dharmacon #MHS6278-202829247). For transfection and Flp-In cell line construction, the full sequences of TET2, ALKBH1, MPP8, TET1(CD) Mut, and the catalytic domain (CD) of TET1 (aa 1418-2136) were cloned into a modified pcDNA5/FRT/TO vector containing an N-terminal 3xFLAG tag. For protein expression, ALKBH1 was cloned into pGEX-6P-1. For the generation of KO cell lines, DNA oligos coding for guide RNA (gRNA) sequences were cloned into pX330-U6-Chimeric_BB-CBh-hSpCas9 (Addgene, #42230). The oligos were phosphorylated with T4 PNK (NEB, M0201) and ligated with T4 DNA ligase (NEB, M0202) into a pX330 backbone digested with BbsI-digested (NEB, #R3539). For the dual-luciferase assay, reporter plasmids were generated by overlap PCR from pcDNA3 RLUC POLIRES FLUC (Addgene #45642) and ligation into pCDNA3.1 (Invitrogen). TTA reporter plasmid, TTG reporter plasmid, and random reporter plasmid were obtained by subcloning TTATTATTATTATTA, TTGTTGTTGTTGTTG, or AGCCCCGGGGAGCTC linkers, respectively, between the Renilla luciferase (RLuc) and Firefly luciferase (FLuc) portions.

### General cell culture

Wild-type (WT) HEK 293T, and Flp-In TRex 293 cells were grown at 37 °C in a humidified atmosphere with 5% CO2 in DMEM (Life Technologies) supplemented with 10% fetal bovine serum (Atlanta), 1x penicillin-streptomycin (Gibco Life Technologies) and 2 mM l-glutamine (Life Technologies). For stress treatments when applicable, cells were heat-shocked at 42 °C for 1 h, treated for 1 h with 0.5 mM NaAsO_2_ (Sigma), treated for 4 h with 0.2 mM NaAsO_2_, or grown for 8 h in FBS- and glucose-free DMEM supplemented with 5 mM glucose (Gibco).

### 293 Flp-In cell line generation

To generate stable cell lines expressing 3xFLAG-tagged ALKBH1, TET2, and MPP8, Flp-In TRex 293 cells were seeded at 0.6 × 10^6^ cells per well in six-well plates, and co-transfected with the appropriate pCDNA5/FRT/TO-3xFLAG-tagged plasmid (0.2 µg) and pOG44 plasmid (2 µg, Thermo Fisher). After selection with 100 µg/mL hygromycin B and 15 µg/mL blasticidin, colonies were expanded. To confirm expression of the desired protein, cells were induced with tetracycline (0–1 µg/mL) for 24 h. Cells were harvested and lysed in NP-40 lysis buffer [50 mM Tris HCl pH 7.5, 150 mM NaCl, 5 mM MgCl_2_, 0.5% NP-40, 1 mM PMSF (supplemented freshly), protease inhibitor tablet (Roche, supplemented freshly)]. Protein concentration was normalized to 2 mg/mL, and proteins were separated by SDS-PAGE and analyzed by western blot with anti-FLAG M2 antibody (Sigma, #F1804, 1 µg/mL, 1:1000).

### Knockout (KO) cell line generation

HEK293T WT cells (0.8 × 10^6^) were seeded in a six-well dish. After 24 h, pX330 plasmid containing the gRNA for the target protein (2 µg) and pcDNA3-FKBP-EGFP-HOTag3 (Addgene, 200 ng) were co-transfected using Lipofectamine 2000 (Thermo Scientific). Cells were FACS-sorted 48 h after transfection. The top 95% of cells with green fluorescent protein (GFP) signals were sorted as single cells into 96-well plates. Surviving colonies were expanded to 6-well plates and then 10-cm plates. KO was confirmed by genomic PCR and western blot with mouse β-actin antibody (Cell Signaling, 1:1000, as a loading control) and either rabbit anti-ALKBH1 (Abcam #128895, 1:2000), anti-TET2 (Novus #NBP2-32104, 0.2 µg/mL) or anti-NSUN2 (Proteintech, #20853-1-AP 1:2000) antibodies.

### Cell viability assay after 5-EC treatment

HEK293T WT cells were seeded in 96-well culture plates (4000 cells in 200 µl of medium per well) on day 0. On day 1, cells were treated with 5-EC in DMSO at the final concentrations of 0, 10, 50, 100, 200, 500, 1000, 2000, and 5000 µM. Cell viability was measured using the MTS assay (CellTiter 96 Aqueous Non-Radioactive Cell Proliferation Assay; Promega, G5430) at 12 h, 24 h, 36 h, and 48 h post-treatment following the manufacturer’s instructions. The absorbance of each well was read at 490 nm on a Synergy H1 Microplate Reader (BioTek). All treated cells were normalized to the 0 µM sample and the curve was fitted based on a 4-parameter dose-response equation using GraphPad Prism:

1 $${{{{{\rm{Y}}}}}}={{{{{\rm{Bottom}}}}}}+({{{{{\rm{Top}}}}}}-{{{{{\rm{Bottom}}}}}})/(1+10\wedge ((\log {{{{{\rm{IC}}}}}}50-{{{{{\rm{X}}}}}})\times {{{{{\rm{HillSlope}}}}}}))$$

Three independent biological replicates and three technical replicates were analyzed.

### Analysis of 5-EC labeling using fluorescence microscopy

WT 293T cells were seeded on a round glass coverslip at 0.6 × 10^6^ cells per well in a 6-well plate. Cells were allowed to grow 18–24 h and were treated with 1 mM 5-EC or vehicle for 4 h. After labeling, media were removed, and the coverslips were washed with PBS three times. Cells were fixed for 20 min at room temperature in PBS containing 3% paraformaldehyde and 2% sucrose adjusted to pH 7.3 and washed with PBS 3 times for 5 min each time. Cells were permeabilized with PBST (PBS + 0.1% Triton X-100) for 20 min at room temperature and washed 3 times with PBS for 5 min each time. Cellular RNA was labeled by Cu-assisted azide-alkyne cycloaddition (CuAAC) by incubating each coverslip upside-down (cell-side down) onto droplets (100 µL) of “click” solution (5 µM Cy3-Azide, 1 mM CuSO_4_, 2 mM THPTA ligand, 10 mM Na ascorbate, 1X PBS) for 2 h at room temperature in the dark. Coverslips were washed briefly 3 times with PBST and then rewashed more thoroughly 5 times with PBST for 10 min each time to remove any remaining dye. Nuclei were stained with Hoechst 33342 (Thermo, 1 ug/mL) in PBS for 5 min, and coverslips were washed with PBS twice for 5 min. Coverslips were mounted on cell-face down in ProLong Gold AntiFade Reagent (Life Technologies) atop a microscope slide and fixed in place with nail polish. Fixed cells were imaged on a Nikon Eclipse Ti microscope equipped with a 100x objective, a CMOS camera, and the NIS Elements AR software. The TRITC channel was used to excite the Cy3 dye. Images used for direct comparison were acquired using standardized illumination and exposure parameters and presented with identical LUT settings.

### 5-EC-mediated in vivo crosslinking of TET1(CD)

WT 293T cells were seeded at 0.6 × 10^6^ cells/well in six-well plates and transfected with the pcDNA5-TET1(CD) plasmid (2 μg) and Lipofectamine 2000 (Invitrogen, 5 μL) following the manufacturer’s instructions. Twelve (12) h post-transfection, cells were treated with fresh media containing 5-EC or 5-EU (0.2–1 mM) for 16 h. Cells were harvested and lysed in NP-40 lysis buffer and incubated on ice for 30 min. The lysate was spun at 21,000 × *g* at 4 °C, normalized to 2 mg/mL protein, run on a 10% SDS-PAGE gel, and analyzed by western blot (anti-FLAG) as described above.

### Poly(A) pulldown of 5-EC-treated cells for proteomics (5-EC RNABPP) or western blot

Flp-In TRex 293 cells expressing the desired protein (for western blot validations) or WT HEK 293 T cells (for proteomics experiments) (5–10 × 10 cm plates) were grown to 50% confluency, induced with tetracycline (1 µg/mL), and cultured for 24 h to ~80% confluency before overnight treatment with 1 mM 5-EC (500 mM stock in DMSO) or vehicle (or also m^5^C in Supplementary Fig. 8B). Cells were harvested and poly(A) pulldown was performed following published protocols with minor modifications^68,69^. 1 mL of oligo(dT) lysis buffer [20 mM Tris HCl pH 7.5, 500 mM LiCl, 0.5% LDS, 1 mM EDTA, 5 mM DTT (supplemented freshly), and protease inhibitor tablet] and 75 µL of equilibrated oligo(dT) beads were used per plate. Lysis, enrichment, and washes were performed at ambient temperature, and washes were performed with lysis buffer, NP-40 washing buffer (50 mM Tris HCl pH 7.5, 150 mM LiCl, 0.5% NP-40, 1 mM EDTA, 0.5 mM DTT), and NP-40 free washing buffer (just for proteomics experiments). Bead-bound RNA was eluted with oligo(dT) elution buffer [(40 µL/plate), 20 mM Tris HCl pH 7.5, 1 mM EDTA] for 5 min at 80 °C with shaking. The incubation-wash-elution procedure was repeated 2 more times, and the elutions combined and concentrated to ~75–50 µL. RNA in the elutions was digested with 25 unit/mL RNase A and 2000 unit/mL RNase T1 in 40 mM NaCl and 2 mM MgCl_2_ at 37 °C for 1 h. The remaining protein and the input were run on a 10% SDS-PAGE gel analyzed by western blot (anti-FLAG). Experiments were performed in triplicate.

### Proteomics mass spectrometry

Sample volume was adjusted to 200 µL with 50 mM Ammonium Bicarbonate pH 8 and incubated with 5 mM TCEP at 60 °C for 10 min and then with 15 mM chloroacetamide in the dark at RT for 30 min. Proteins were digested with Trypsin Gold (Promega, 2 µg) and incubated at 37 °C for 16 h. Samples were acidified 0.2% TFA and desalted using SDB stage-tips^70^. Samples were dried processed for mass spectrometry on an Orbitrap Fusion Lumos (Thermo Scientific) as described previously^71^. Data were analyzed using Scaffold (version Scaffold 4.9, Proteome Software Inc.) to validate MS/MS-based peptide and protein identifications. Quantitation of protein abundance was done by spectral counting. Proteins were only selected for quantification if they were present in at least two replicates and not in the negative control.

### Ribonucleoside quantification from biological samples by LC-QQQ-MS

Total RNA was extracted using TRIzol reagent following the manufacturer’s indications. Small RNA was isolated from total RNA using the adjusted protocol from RNA Zymo Clean and Concentrator 5 (Zymo Research). mRNA was isolated by 2 rounds of poly(A) pulldown as described above and further subjected to ribo-depletion with custom synthesized probes complementary to human rRNA^72^ and small RNA depletion (with Zymo Clean and Concentrator 5. RNA samples (3 µg) were digested in 20 µL with 2 units of Nuclease P1 (Wako) and final concentrations of 7 mM NaOAc and 0.4 mM ZnCl_2_ at 37 °C for 2 h. The digested mixture was subjected to dephosphorylation in a total volume of 30 µL with 2 units of Antarctic Phosphatase (AnP, NEB) and a final concentration of 1X AnP buffer at 37 °C for 2 h. Quantification of ribonucleosides was done an Agilent 1260 LC Infinity II system coupled to an Agilent 6470 LC/TQ module as reported previously^21^ and with a separation gradient from literature precedence^73^. Analyte detection was done by dynamic multiple reaction monitoring (DMRM) with the parameters in Table S1. Abundant nucleosides in biological samples (ACGU) were quantified in 1 ng of sample, while less abundant ones (m^5^C, 5-EC, hm^5^C, f^6^C, i^6^A) were analyzed in 100–200 ng. Commercial nucleosides were used to generate standard curves. Relative levels of m^5^C, 5-EC 5-EU, hm^5^C, f^6^C, and i^6^A were calculated by normalizing the concentration of modified nucleoside to that of the respective canonical nucleoside.

Metabolic incorporation of 5-EC was performed by feeding WT 293T cells at ~80% confluency with 1 mM 5-EC (500 mM stock) or vehicle in fresh DMEM for 16 h. RNA was processed for QQQ LC-MS/MS quantification on Agilent MassHunter Workstation Data Acquisition version 10.0 as described above using relevant standards (Supplementary Figs. 1–2). The experiment was done in triplicate.

### 5-EC-iCLIP

Library preparation for iCLIP was adapted from the literature^45^. 30 × 10 cm dish Flp-In T-Rex 293 cells expressing 3xFlag-ALKBH1 were grown to 60% confluency and treated with 1 μg/mL tetracycline. After 12 h, medium was changed to fresh medium containing 1 μg/mL tetracycline and 1 mM 5-EC and cultured for another 12 h. Cells were washed twice with cold PBS and harvested with 500 µL lysis buffer (50 mM Tris-HCL pH 7.4, 100 mM NaCl, 1% NP-40, 0.1% SDS and 0.5% sodium deoxycholate). Lysates were treated with Turbo DNase (Thermo) and a low (1:200) or high (1:50) concentration of RNase I (AM2295) at 37 °C for 3 min with rotation, after which they were centrifuged at 20,000 × g for 15 min at 4 °C. The supernatant was incubated with anti-FLAG beads (200 μL Protein G beads, 15 µg anti-Flag M2 antibody) at 4 °C overnight, and the immunoprecipitated material was washed three times with high-salt buffer and then once with CutSmart buffer (NEB). The beads were treated with Quick-CIP (NEB) at 37 °C for 30 min with rotation, washed twice with high-salt buffer, and then incubated with pre-adenylated and biotin-labeled L3 linker by using T4 RNA ligase I (NEB) at 16 °C overnight. The beads were boiled and analyzed by Western blot for biotinylation with the chemiluminescent nucleic acid detection module (Thermo). The rest of the sample was gel purified based on the position of the biotin signal by using D-tube dialyzer midi (Merck-Millipore). Proteinase K-digested and purified RNA was used for reverse transcription with oligos containing randomized barcodes (UMIs) and two inversely oriented adaptor regions separated by a BamHI restriction site. cDNAs were size-purified (70–75 nt, 75–100 nt and 100–150 nt) on TBE-Urea gels and then circularized by CircLigase II (Epicentre). Circularized cDNAs were digested with BamHI and linearized cDNAs were amplified using Solexa primers and submitted for Illumina sequencing.

### Bioinformatic analysis

The iCLIP and ARP pulldown sequencing data was processed using iCount Primary Analysis (Consensus mapping) pipeline on iMAPs web server (https://imaps.genialis.com/iclip). Briefly, random unique molecular identifiers were used to distinguish and discard PCR duplicates, and adaptor/barcode sequences were removed. Trimmed reads were mapped to tRNA/rRNA with STAR (v.2.7.0f). The unmapped reads were further mapped to GRCh38 with STAR (v.2.7.0f) and only uniquely mapping reads were used for further analysis. For iCLIP: iCount generated raw crosslinking sites were used for peak calling analysis by Paraclu with following parameters: Minimal sum of scores inside a cluster:10; Maximal cluster size: 6; Minimal density increase:1. For ARP pull down: peaks were called using Paraclu with default parameters. Only peaks present in two replicates and unique to the 5-EC-treated (IP sample in ARP analysis) sample or showing a fold-change of greater than 1.5 (treated versus input) were kept after intersection. Sequences 5 bases upstream and downstream were extracted from the reference and used for motif analysis by MEME (V.5.3.3) using the default parameters.

### ALKBH1 expression and purification

The sequence-verified construct pGEX-6P-1-ALKBH1 was transformed into *E. coli* strain BL21 and expressed at 18 °C with 0.2 mM isopropyl-β-D-thiogalactopyranoside (IPTG) for 18 h. Lysis by sonication was performed in buffer containing 1X TBS, 150 mM NaCl, 5 mM EDTA, 1 mM DTT, 0.2 mg/mL lysozyme (Thermo), and 1% Triton X-100, supplemented with 1 mM PMSF, protease inhibitor tablet, and Benzonase (Sigma). The lysate was purified using Pierce glutathione agarose resin (ThermoFisher) following the manufacturer’s instructions. The GST tag from promising fractions was cleaved with PreScission protease (Sigma, 1:100 by mass of protease:sample) while simultaneously dialyzing in 1x TBS at 4 °C overnight. Cleavage was confirmed by SDS-PAGE, and the free GST tag was removed by running the sample over fresh or regenerated glutathione resin and collecting the flow-through. The protein was further fractionated on a MonoQ 5/50 GL anion exchange column (GE Healthcare) on a gradient of 50 mM to 1 M NaCl (in 50 mM Tris HCl pH 8) for 20 column volumes. The most concentrated fractions were combined dialyzed overnight at 4 °C in 50 mM Tris HCl pH 7.5 and concentrated to at least 1 mg/mL.

### Oligonucleotide synthesis

Oligonucleotide synthesis was done on an ABI 394 oligonucleotide synthesizer (Applied Biosystems) using standard coupling conditions and commercial oligosynthesis reagents and phosphoramidites (Glen Research) unless otherwise noted. For synthesis of the random m^5^C-containing RNA library (oligo **5**), a mix was created by combining each TBDMS-protected RNA phosphoramidite (i.e., A, G, C, U) in the ratios previously reported^74^. Oligos **3**–**4** and **6**–**8** were cleaved from the resin and 2’OH-deprotected and butanol-precipitated under standard conditions.

Oligos **1**–**2** were synthesized with 5-EC phosphoramidite (**11**, Supplementary Note) or m^5^C phosphoramidite (Glen Research), respectively, using ultra mild phosphoramidites (Glen Research). The final product was manually detritylated one last time with 3% dichloroacetic acid on the instrument until the orange coloration observe in the lines (from the trityl cation) disappeared. The oligos were cleaved from the resin in a solution of 3:1 NH_4_OH/EtOH at rt for 24 h with rotation, and the supernatant was dried on speedvac. 2’-OH deprotection was performed on the residue with 1 M TBAF on THF (1 mL) at rt for 18 h with rotation. The solution was diluted with THF (1 mL), desalted on NAP-10 columns (G&E healthcare, 2 columns per sample), eluted with 2.5 mL DEPC-H_2_O, and lyophilized. Incompletely lyophilized samples were precipitated with 1 mL EtOH + 100 μL 3 M NaOAc pH 5.2, dried via speedvac, resuspended in H_2_O, and re-lyophilized.

All oligos except for **5** were purified by reverse-phase HPLC on a Zorbax Eclipse XDB-C18 semiprep column (Agilent) using a gradient of 5 to 40% acetonitrile in 0.1 M triethylammonium acetate over 50 min or 0 to 20% acetonitrile over 30 min and characterized by ESI-TOF HRMS and MALDI-TOF MS (Supplementary Table 4). Oligo 3 was purified and characterized by denaturing urea-PAGE (Supplementary Fig. 36).

### ALKBH1 activity assay on m^5^C-containing substrates

ALKBH1 enzymatic assays were carried out as reported previously^19,20^. Briefly, RNA substrate (125 pmol) was reacted with 0.1 µM ALKBH1 in a 250 µL reaction containing 20 mM HEPES pH 6.8, 5 mM MgCl_2_, 50 mM KCl, 1 mM DTT, 4 mM ascorbic acid, 100 µM 2-oxoglutarate, and 80 µM ammonium iron (II) sulfate. The mixture was incubated at 37 °C for the time indicated in Fig. 4C and immediately quenched by performing RNA extraction with TRIzol LS (Thermo) following the manufacturer’s directions up to the layer separation step. The aqueous layer was then transferred to a Zymo RNA Clean and Concentrator-5 spin column, and the purification was resumed following the manufacturer’s indications. The entire RNA sample was digested with Nuclease P1 and dephosphorylated with AnP as described above. Decrease of m^5^C and generation of hm^5^C and f^5^C were measured in 6.25 pmol of the sample by LC-QQQ-MS using commercial standards. Experiments were performed in triplicate.

### In vitro ALKBH1 crosslinking assays with 5-EC oligo

Crosslinking reactions were prepared in 20 μL with the following concentrations of reagents: 50 mM tris pH 8, 50 mM KCl, 2 mM ascorbic acid, 1 mM MgCl_2_, 1 mM alpha-ketoglutarate, 100 μM FeCl_2_, 1 μM RNA oligo, and 0.2 μM recombinant ALKBH1. The reactions were incubated for 1 h at 37 °C and quenched with the addition of 1 μM 0.5 M EDTA or 10 μL 3x SDS sample buffer for Western blotting. Crosslinked protein was separated on 12% SDS-PAGE and Western-blotted for ALKBH1 as indicated above.

### Antisense pulldowns and northern blotting

Specific tRNAs were enriched by antisense pulldown with biotin-labeled antisense probes in Table S16 following a modified procedure from the literature^42^. Small RNA was extracted from WT or ALKBH1 KO 293 T cells using the small RNA enrichment protocol from Zymo RNA Clean and Concentrator-5 (Zymo). Depending on tRNA abundance and probe efficiency, the pulldown was done on 5–25 µg of bulk small RNA. Streptavidin M-280 magnetic beads (Invitrogen) (10 µL bead per 5 µg RNA input) were used. RNA and probe-conjugated beads were incubated at 42 °C for 3 h. Elution was performed twice at 75 °C for 5 min in 10 mM Tris pH 7.5.

40–100 ng of small RNA input or pulldown elution were resolved in a 12% polyacrylamide-urea gel and analyzed by Near-IR northern blot following literature precedence with minor adjustments^75^. RNA was transferred overnight to a Biodyne B Nylon membrane (Thermofisher) and immobilized under a 254 nm UV lamp for 10 min. Blocking was done in pre-hybridization buffer (200 mM Na_2_HPO_4_, 7% SDS,) supplemented with 50 µg/mL of salmon sperm DNA for 2 h at 42 °C. Annealing was done in pre-hybridization buffer for 4 h at 42 °C with 50 nM of the corresponding biotinylated probe (Table S16). Co-hybridization with a FAM-labeled probe (50 nM) complementary to 5S RNA (Table S16) was performed in cases where a loading control was necessary. Secondary staining was performed with IR-800 Streptavidin dye (1:10 000, LICOR) in pre-hybridization buffer for 30 min at 30 °C.

For LC-QQQ-MS measurements, the tRNA elutions were dephosphorylated and analyzed as indicated above. Triplicates were gathered for each experiment.

### Pyridine borane treatment

1 ug total RNA was incubated with 600 mM sodium acetate (pH 5.2) and 1 M pyridine borane for 16 h at 37 °C in water. The product was purified on Zymo-Spin columns. 100 ng reacted RNA was incubated with 10 μM RT primer and 1 μL 10 mM dNTP mix at 65 °C for 5 min. The reaction was reverse transcribed with Superscript II following the manufacturer’s instructions. cDNA was then PCR amplified by AccuPrime™ Pfx DNA Polymerase (Thermo). The PCR products were gel purified and submitted for high-throughput amplicon sequencing (Genewiz).

### Mutation detection by amplicon sequencing

The reads were first mapped to tRNA reference sequences by STAR (Galaxy Version 2.7.8a). Only uniquely mapped reads were kept for further analysis. Pileup files were generated using Samtools mpileup (Galaxy Version 2.1.4) with default parameters. The variants were called using VarScan mpileup (Galaxy Version 2.4.3.1) with the default parameters.Mutations that were only present in WT samples but not ALKBH1 KO samples and control samples were identified as ALKBH1-dependent f^5^C sites.

### Aldehyde reactive probe (ARP) sequencing

Total RNA extracted from WT or ALKBH1 KO cells was first treated with 2 μl DNase I (NEB) to remove DNA contamination. The total RNA was then fragmented into 150 nt fragments using RNA Fragmentation Reagents (Thermo). The incubation of fragmented RNA samples with Aldehyde Reactive Probe (ARP) was carried out with some modification from a previous report^53^. Briefly, 100 ng f^5^C containing oligo was first added into 100 μg fragmented RNA, the mix was then incubated with 2 mM ARP in 40 mM aqueous NH_4_OAc buffer pH 5.0 supplemented with 100 mM anisidine for 24 h at 25 °C. RNA was then purified using phenol/chloroform extraction. 200 ng RNA was taken out as input, and the rest of the samples were incubated with 50 μl pre-washed streptavidin beads in 400 μl IP buffer (200 mM NaCl, 0.4 mM EDTA, 50 mM Tris-HCl pH 7.4, and 0.1% NP-40) for 2 h at 4 °C. The beads were then washed twice with IP wash buffer (500 mM NaCl, 0.4 mM EDTA, 50 mM Tris-HCl pH 7.4, and 0.1% NP-40). The beads and input samples were treated with Quick-CIP (NEB) at 37 °C for 30 min with rotation, input samples were purified using RNA Zymo Clean kit (Zymo Research), and beads were washed twice with wash buffer. Input RNA and bead were then incubated with pre-adenylated L3 linker (rAppAGATCGGAAGAGCGGTTCAG/ddC/) by using T4 RNA ligase I (NEB) at 16 °C overnight. On-bead reverse transcription was performed using Superscript III with iCLIP RT primers (25 °C for 5 min, 42 °C for 20 min, and 50 °C for 40 min). cDNA was eluted using RNaseH and then size-purified (60–100 nt and 100–200 nt) on TBE-Urea gels. Extracted cDNA was circularized by CircLigase II (Epicentre). Circularized cDNAs were digested with BamHI and linearized cDNAs were amplified using Solexa primers and submitted for Illumina sequencing.

### Luciferase translation efficiency assay

For the translation assay, 500 ng of each reporter plasmid were transfected with lipofectamine 2000 into WT HEK293T or ALKBH1 KO cells in a 24-well plate. After 4 h, cells were washed with PBS and changed to glucose deprivation medium (glucose-depleted DMEM supplemented with 10% Pen/Strep, 1 mM or 5 mM glucose) or normal medium (glucose-depleted DMEM supplemented with 10% Pen/Strep, 25 mM glucose) and grown overnight. Cells were harvested in passive lysis buffer (Promega) following the manufacturer’s protocol. The assay was performed using Dual-luciferase Reporter Assay (Promega) following instructions from the manufacturer. Luciferase signal was measured in a Synergy H1 Microplate Reader (BioTek). Fluc signal was normalized to luciferase Rluc signal to evaluate the translation efficiency of each reporter. Three independent biological replicates were analyzed and a student T-test was applied with *p < 0.05 and **p < 0.01.

### Reporting summary

Further information on research design is available in the Nature Research Reporting Summary linked to this article.

## Supplementary information

Supplementary Information

Description of Additional Supplementary Files

Supplementary Data 1

Supplementary Data 2

Supplementary Data 3

Supplementary Data 4

Reporting Summary

## Data Availability

The data that support this study are available from the corresponding author upon reasonable request. The sequencing data generated in this study have been deposited in the GEO database under accession code GSE202815. The proteomics data used in this study are available in the ProteomeXchange database under accession code PXD029955. Source data are provided with this paper.

## Source data

Source Data
