# Supplementary material for: Reactivity-dependent profiling of RNA 5-methylcytidine dioxygenases
Source: Nat Commun. 2022 Jul 19;13:4176. doi: 10.1038/s41467-022-31876-2 (PMC9296451; doi:10.1038/s41467-022-31876-2)
Supplement: Supplementary file 1 — Supplementary Information [file 41467_2022_31876_MOESM1_ESM.pdf]

SUPPLEMENTARY INFORMATION

Reactivity-dependent profiling of RNA 5-methylcytidine dioxygenases

A. Emilia Arguello[1], Ang Li[1], Xuemeng Sun, Tanner W. Eggert, Elisabeth Mairhofer, and Ralph E. Kleiner*

Department of Chemistry, Princeton University, Princeton, NJ 08544, USA

[1]These authors contributed equally.

*rkleiner@princeton.edu

Table of Contents

Supplementary Figures 1-37	S2
Supplementary Tables 1-22	S40
Supplementary Note: Synthetic Protocols and Compound Characterization	S66
References	S86

Supplementary Figures

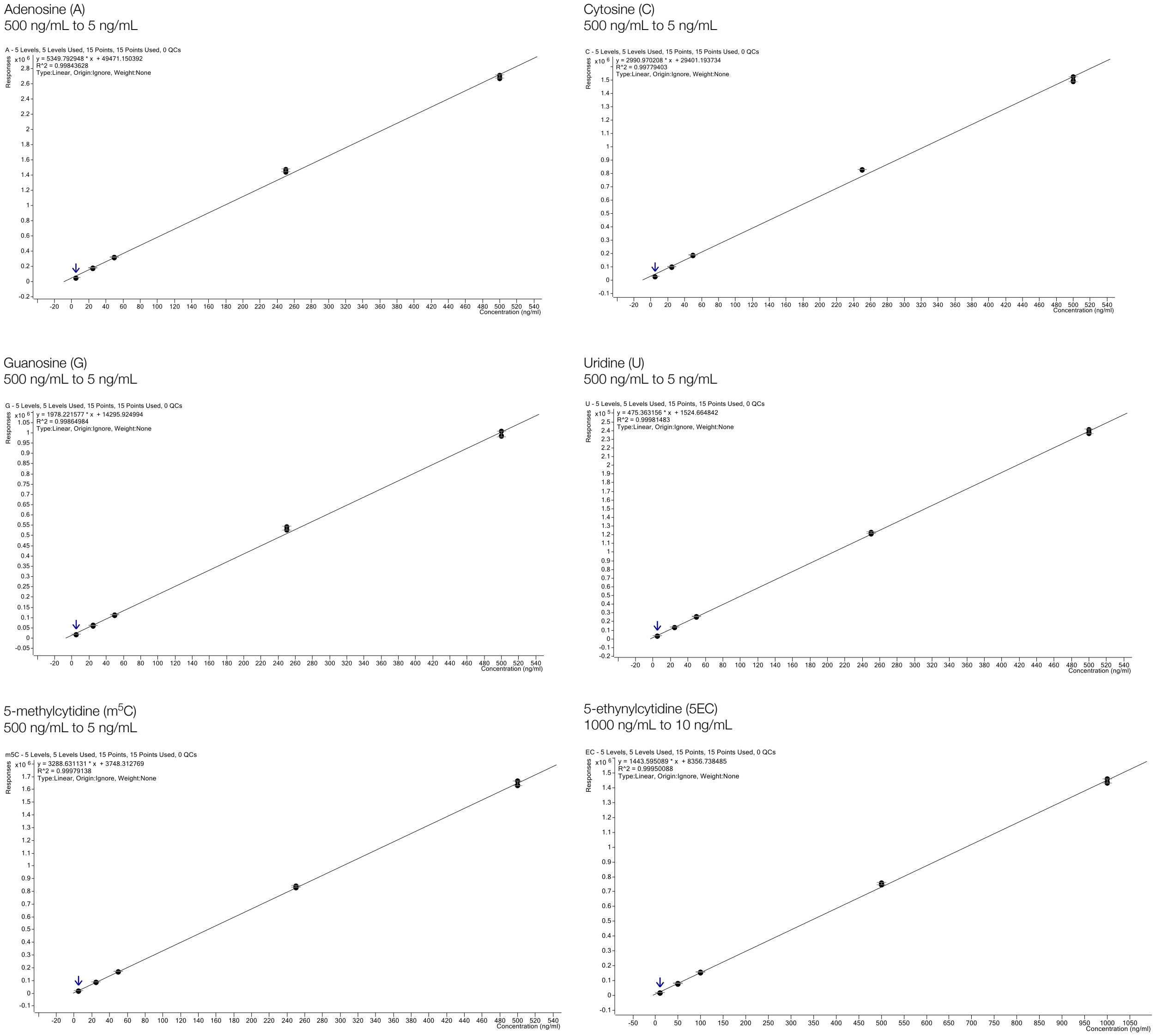

Supplementary Figure 1. Standard curves used for the LC-QQQ-MS quantification of 5-EC incorporation in WT total RNA. Two technical replicates for each standard were used to generate standard curves.

A

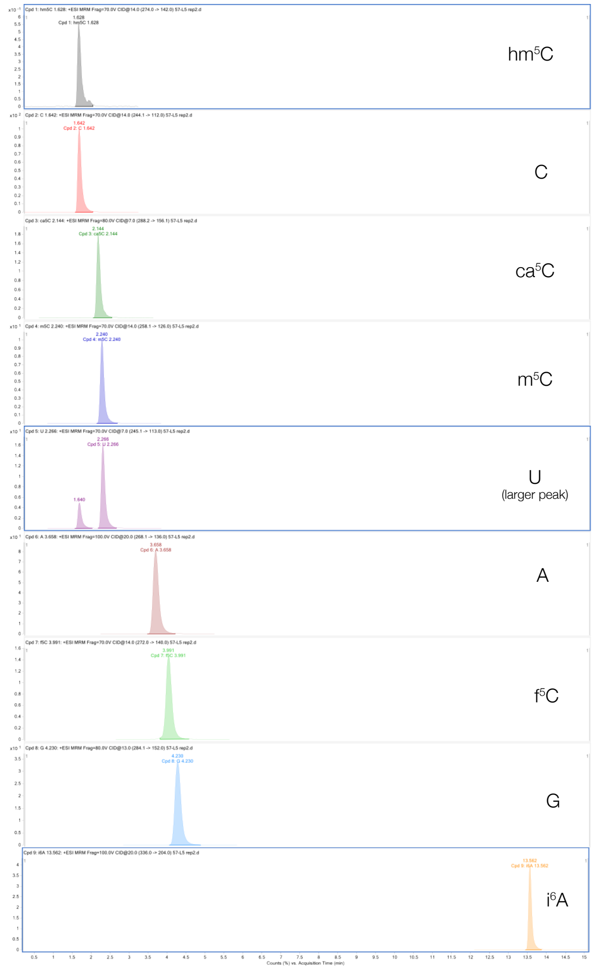

B

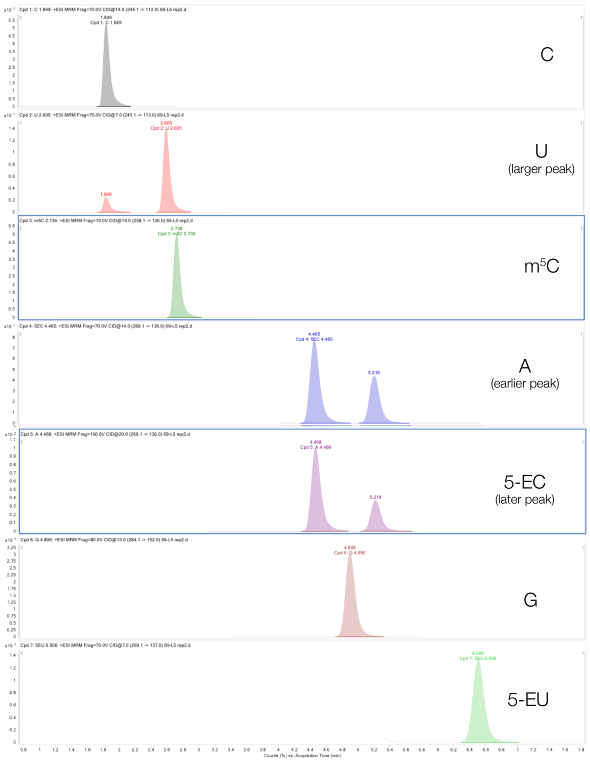

Supplementary Figure 2. Representative extracted ion chromatograms (EICs) from the nucleoside LC-MS/MS measurements. **(A)** EICs of standard mix in the measurement of m^{5}C, hm^{5}C, f^{5}C, ca^{5}C, and i^{6}A in HEK293T WT and ALKBH1 KO total and small RNA. **(B)** EICs of standard mix in the measurement of 5-EC and 5-EU in total RNA of HEK293T WT after metabolic labeling. 5-EC and A have the same parent mass and MRM transition, but different elution times.

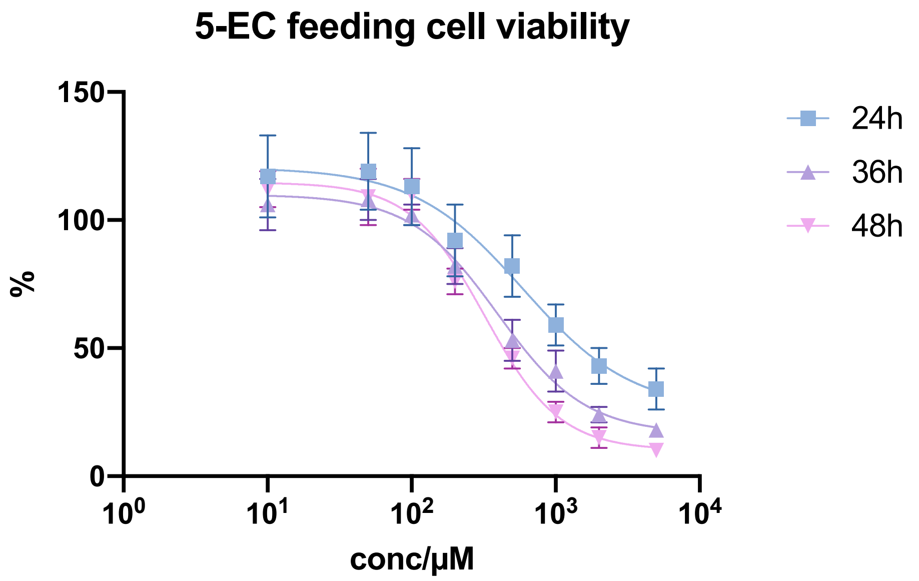

Supplementary Figure 3. Cell viability curves for 5-EC treatment of WT 293T cells. Three independent biological replicates were analyzed. Curves were fitted based on a 4-parameter dose-response equation using GraphPad Prism: IC_{50} values for 24-hr treatment, 592.9 μM; for 36-hr treatment, 396.8 μM; for 48-hr treatment, 323 μM. Data represent mean ± SEM. Source data are provided as a Source Data file.

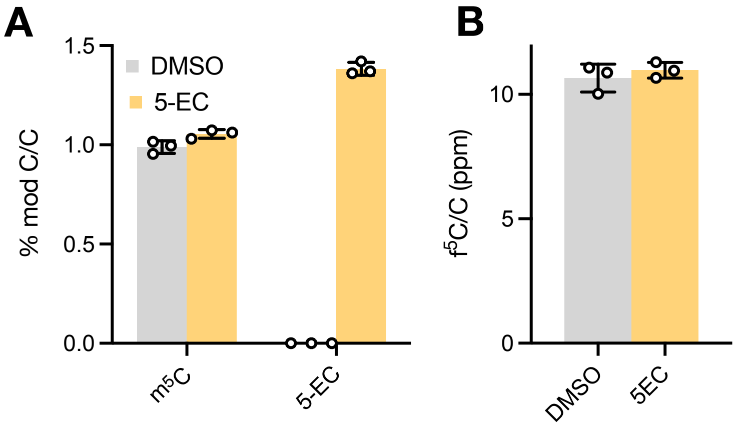

Supplementary Figure 4. 5-EC metabolic labeling does not affect endogenous levels of m^5C or f^5C. (A) Endogenous levels of m^5C and levels of 5-EC incorporation in total RNA of WT 293T cells after overnight treatment with 1mM 5-EC or vehicle as measured by LC-MS/MS. (B) Endogenous levels of f^5C in total RNA of WT 293T cells after overnight treatment with 1mM 5-EC or vehicle as measured by LC-MS/MS. For (A) and (B), three independent biological replicates were analyzed. Data represent mean values ± s.d. Source data are provided as a Source Data file.

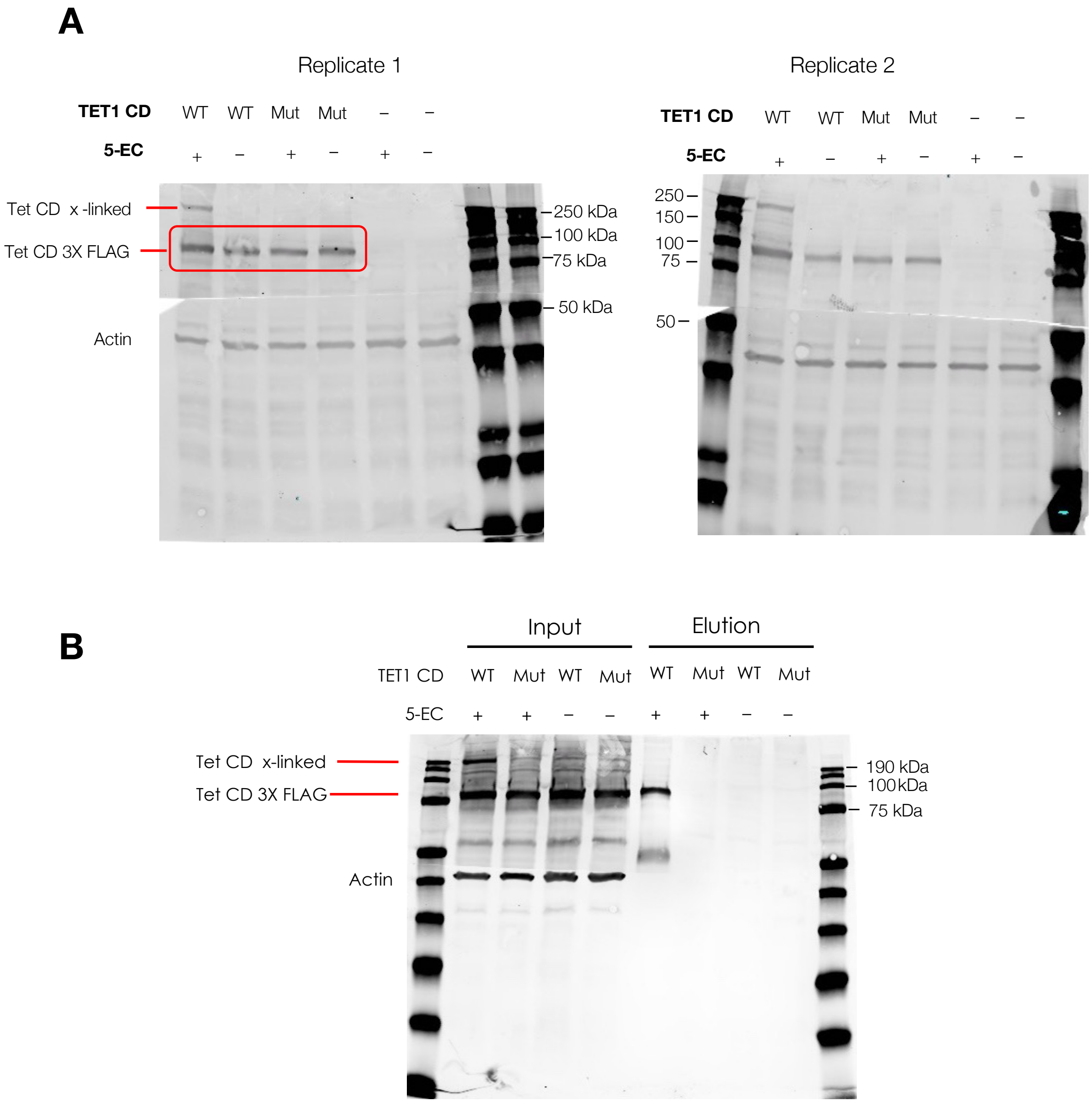

Supplementary Figure 5. 5-EC RNA crosslinks exclusively with catalytically active TET1(CD). **(A)** Wild-type (WT) or an inactive mutant (Mut) pcDNA5-TET1(CD) was transfected into WT 293T, followed by treatment with 5-EC or vehicle for 16 hrs. Cells were lysed, and the lysate was separated by SDS-PAGE. Expression of the construct and the presence of a higher-running RNA-cross-linked band were detected by anti-FLAG western blot. Actin blotting was used as a loading control. **(B)** 5-EC mediated cross-linking and recovery of TET1(CD) WT. 3X-FLAG-TET1(CD) WT or 3X-FLAG TET1(CD) Mut were overexpressed in WT 293T by transient transfection followed by treatment with 1 mM 5-EC for 16 hr. Cells were lysed, and poly-A RNA was isolated via oligo-dT pulldown. RNA was digested and protein was detected by SDS-PAGE and anti-FLAG western blot. For (A) and (B), experiments were repeated three times independently with similar results.

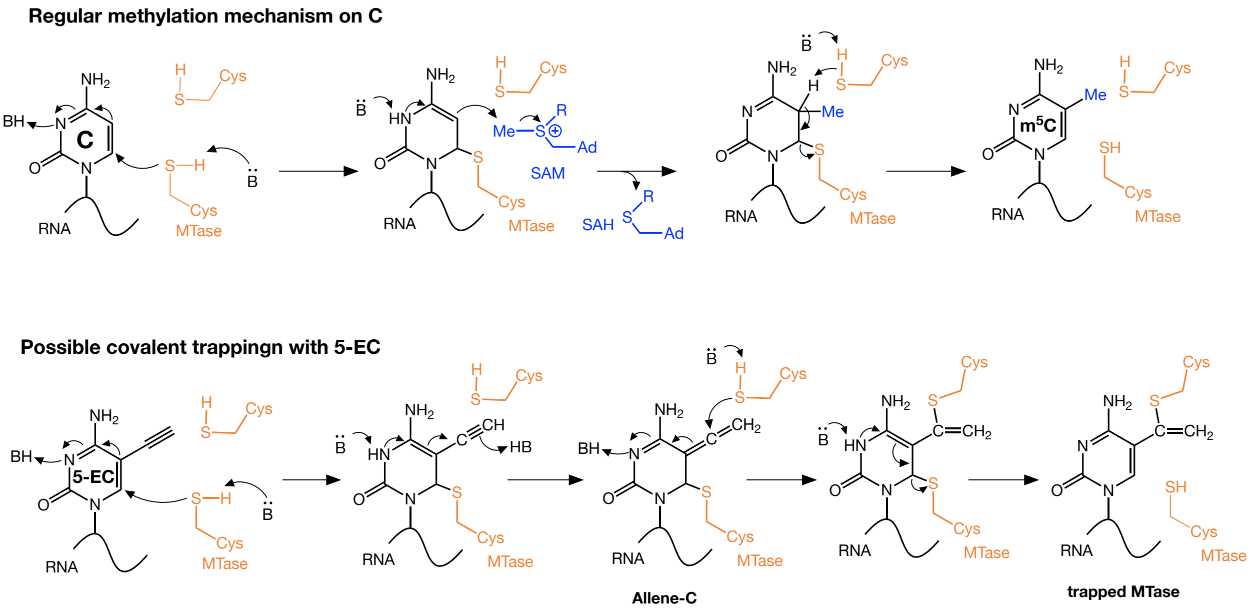

Supplementary Figure 6. Mechanism of methylation at C5 position of cytidine (C) and possible covalent capture with a 5-alkynyl C analog. The proposed mechanism involves the methyltransferase's (orange) two cysteine residues to mediate the transfer of a methyl group from SAM (blue). First, nucleophilic attack of a catalytic Cys residue on the C6 position of C forms an enzyme-bound intermediate, and a second Cys residue mediates beta-elimination after C5 methylation with SAM. With 5-ethynyl cytidine, the first nucleophilic attack allows for a rearrangement of the alkyne to a thiol-reactive allene, which then covalently traps the second cysteine residue.

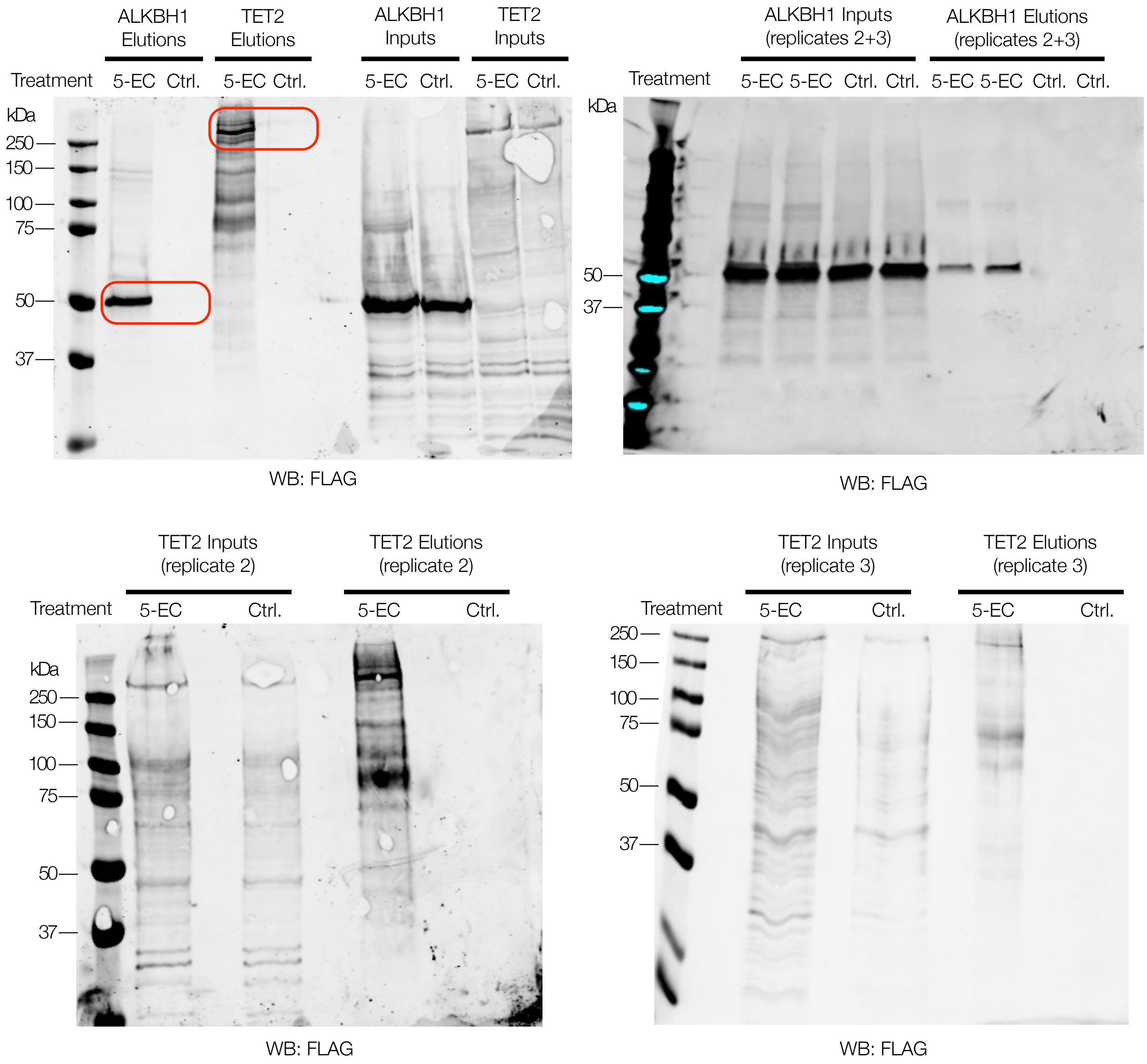

Supplementary Figure 7. 5-EC mediated crosslinking and recovery of ALKBH1 and TET2. Full western blot data for Fig 3C in main text. PolyA RNA isolation was performed as in Supplementary Fig. 5 with the corresponding Flp-In cell line. Highlighted regions are presented in the main text. The experiments were repeated three times independently with similar results.

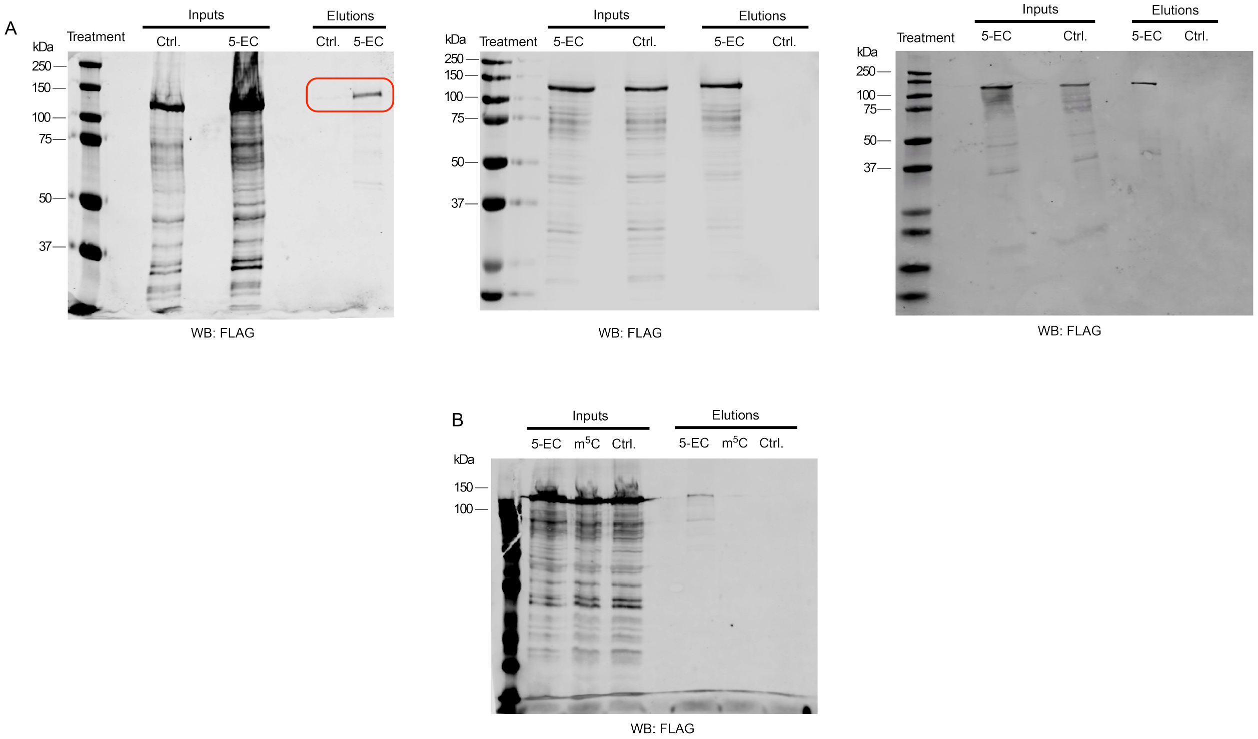

Supplementary Figure 8. (**A**) 5-EC mediated crosslinking and recovery of MPP8. Full western blot data for Fig 3C in main text. PolyA RNA isolation was performed as in Supplementary Fig. 5 with the corresponding Flp-In cell line. Highlighted regions are presented in the main text. (**B**) 5-EC, but not 5-methylcytidine (m^5C), mediates crosslinking of MPP8 to mRNA. The experiment was performed as in Supplementary Fig. 5 with the corresponding Flp-In cell line fed with 1 mM m^5C. For (A) and (B), the experiments were repeated three times independently with similar results.

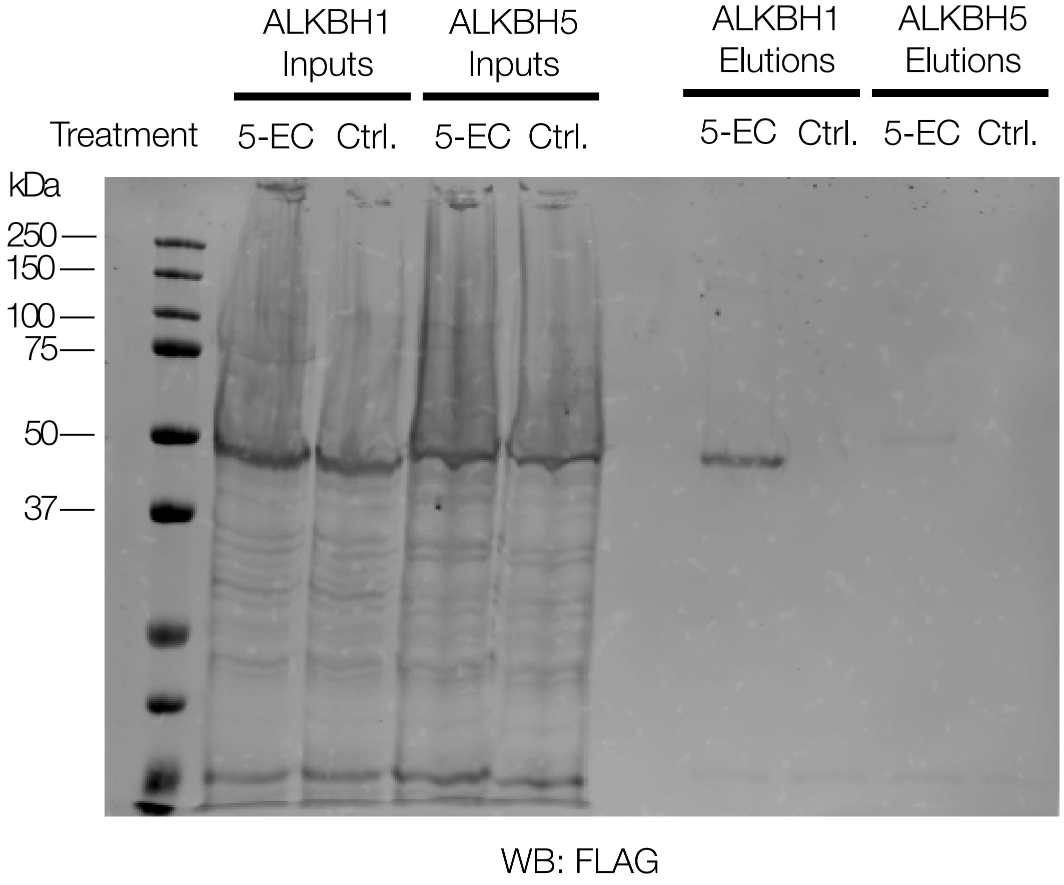

Supplementary Figure 9. 5-EC-containing RNA crosslinks and enriches ALKBH1 over ALKBH5. The experiment was performed as in Supplementary Fig. 5 with the corresponding Flp-In cell lines. The experiments were repeated three times independently with similar results.

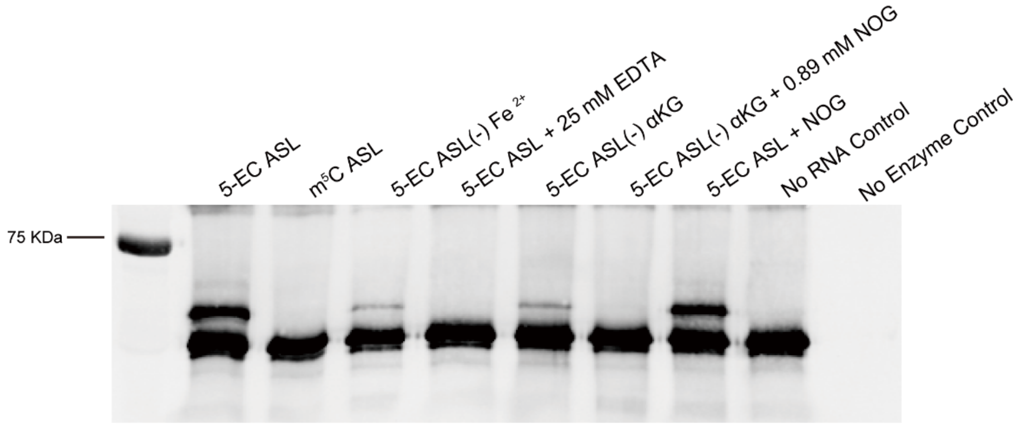

Supplementary Figure 10. *In-vitro* crosslinking of recombinant ALKBH1 and 5-EC-containing $tRNA^{iMet}$ ASL (oligo **1**) or m^5C-containing $tRNA^{iMet}$ ASL (oligo **2**). A higher-running band suggests crosslinked protein-RNA adduct. RNA, enzyme, and cofactors were incubated as described in Methods. The required cofactors Fe^{2+} and alpha-ketoglutarate (α-KG) are included in all reactions unless otherwise indicated. NOG = N-oxalyl glycine, an inhibitor of α-KG-dependent enzymes. The experiments were repeated three times independently with similar results. Source data are provided as a Source Data file.

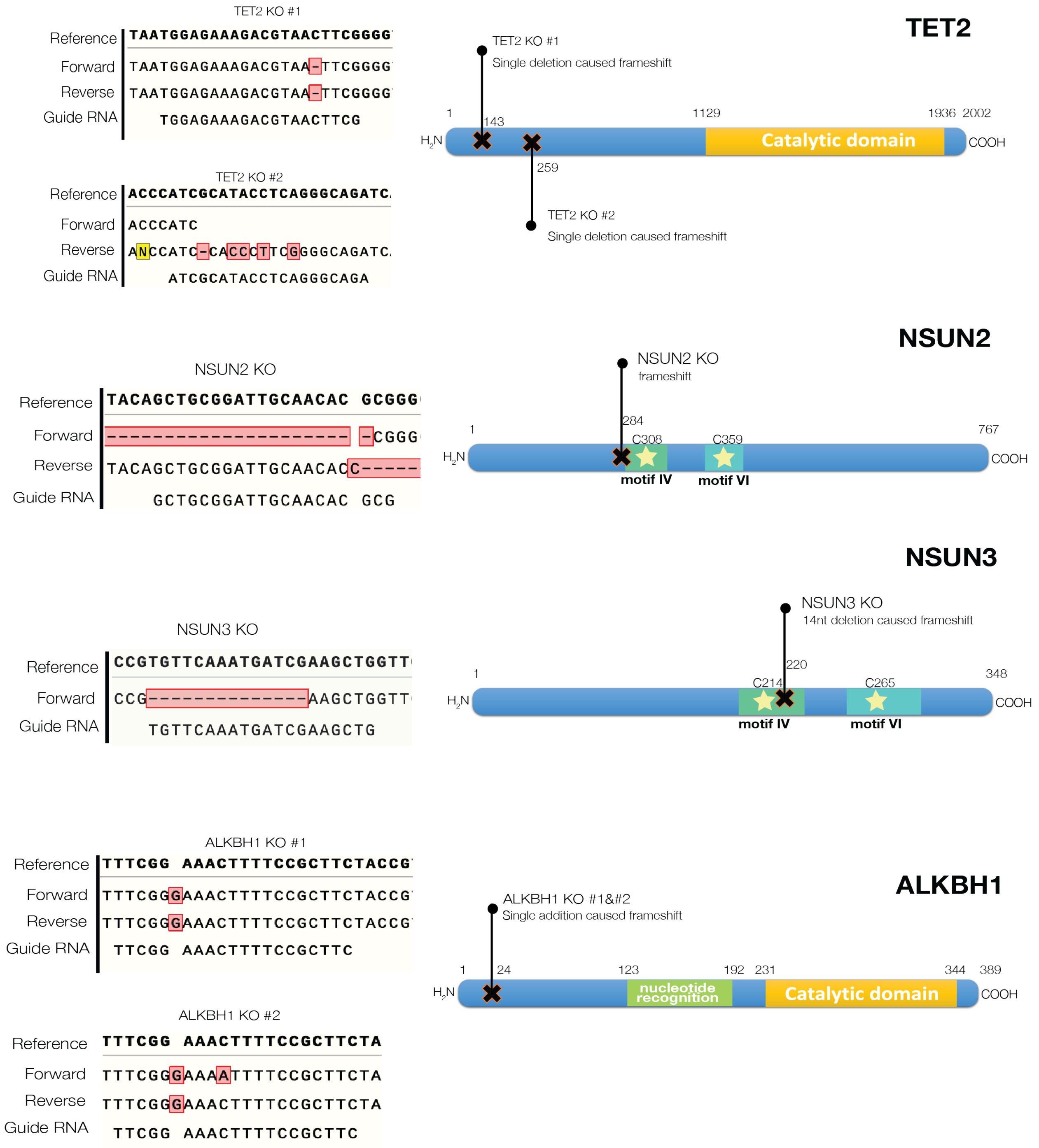

Supplementary Figure 11. Sanger sequencing characterizations of ALKBH1, TET2, NSUN2, and NSUN3 CRISPR knockouts. Cells were harvested, and crude genomic DNA was extracted for genomic PCR and analyzed by Sanger sequencing. Alignment was performed in Snapgene. KOs were chosen if insertions or deletions occurred at or near the editing site (i.e., near the landing site for the guide RNA). Guide RNA for ALKBH1: bases 68-87 of cDNA. Guide RNAs for TET2: bases 414-435 and 774-793 of cDNA.

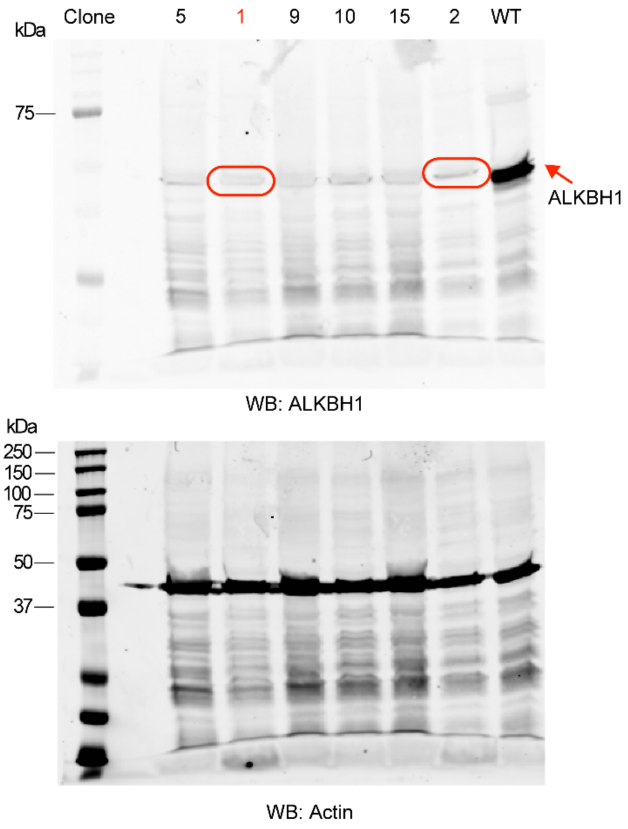

Supplementary Figure 12. Western blot validation of ALKBH1 KO cells. Circled in red are the clones used in LC-MS/MS quantification experiments. The experiments were repeated three times independently with similar results.

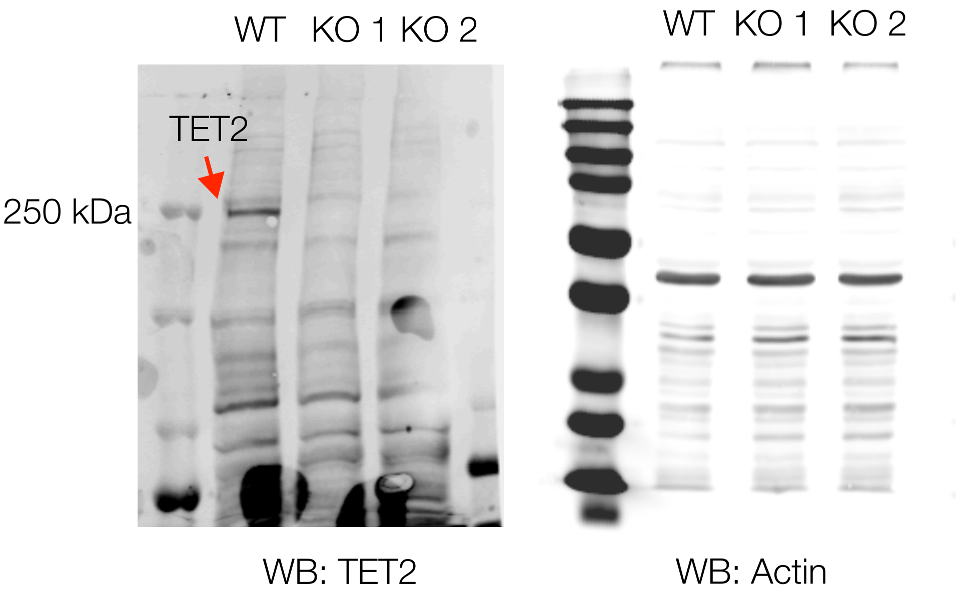

Supplementary Figure 13. Western blot validation of TET2 KO cells. The experiments were repeated three times independently with similar results.

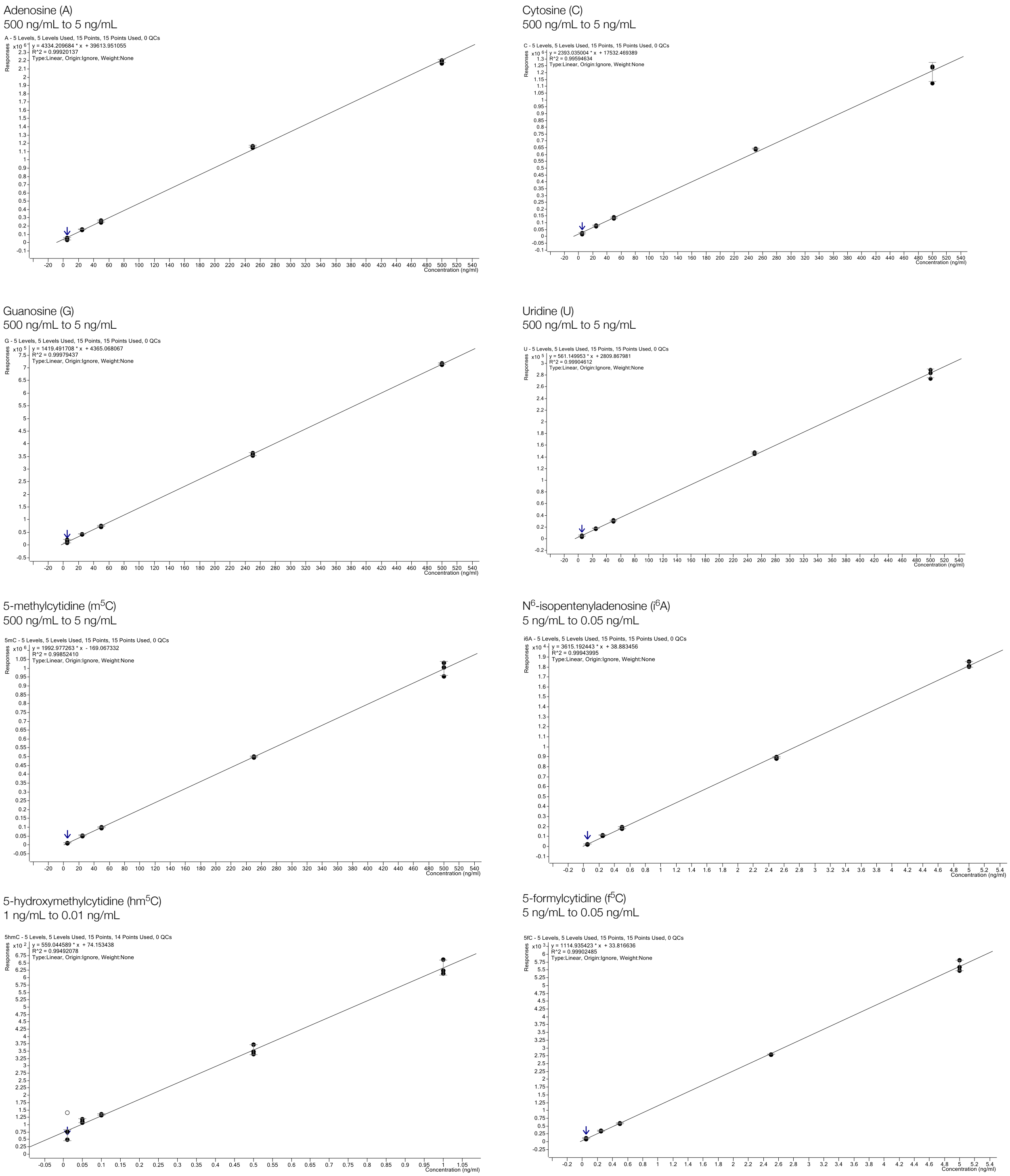

Supplementary Figure 14. Standard curves used for the LC-QQQ-MS quantification of hm^5C and f^5C in RNA from WT and ALKBH1/TET2/NSUN2/NSUN3 KO cells. Two technical replicates for each standard were used to generate standard curves.

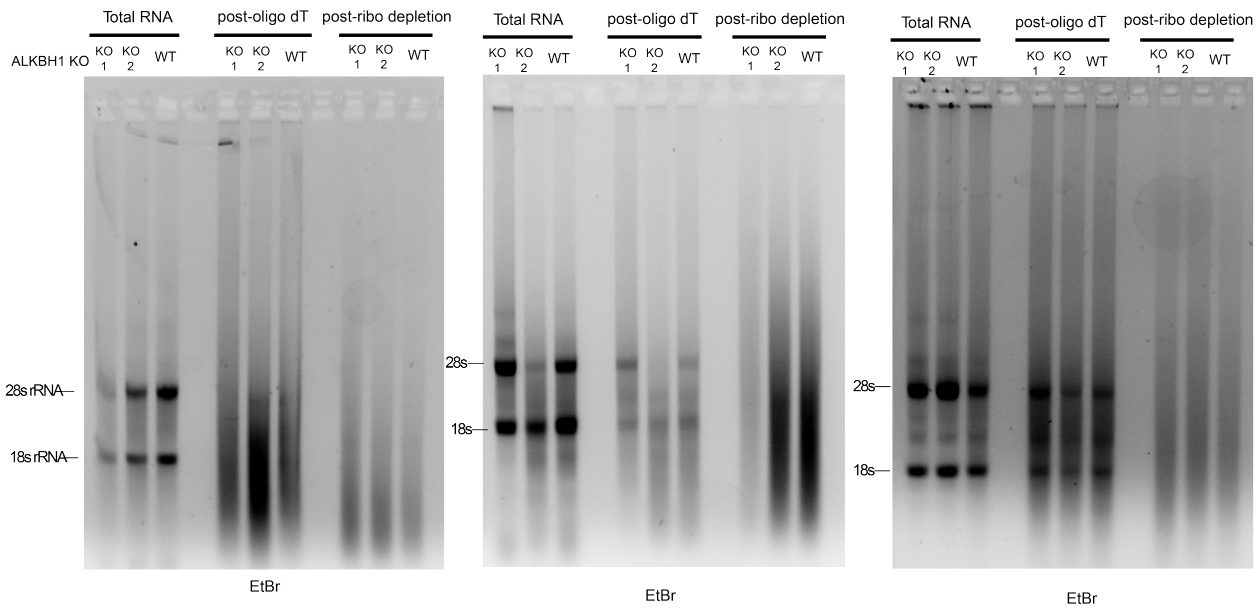

Supplementary Figure 15. Enrichment of mRNA from total mammalian RNA in ALKBH1 KO cells (biological triplicates). Extracted total RNA was first subjected to an oligo(dT) pulldown to isolate poly(A)RNA, which was further enriched by depleting ribosomal and small RNA fragments that co-purified during the pulldown. The quality at each step was checked by agarose gel electrophoresis.

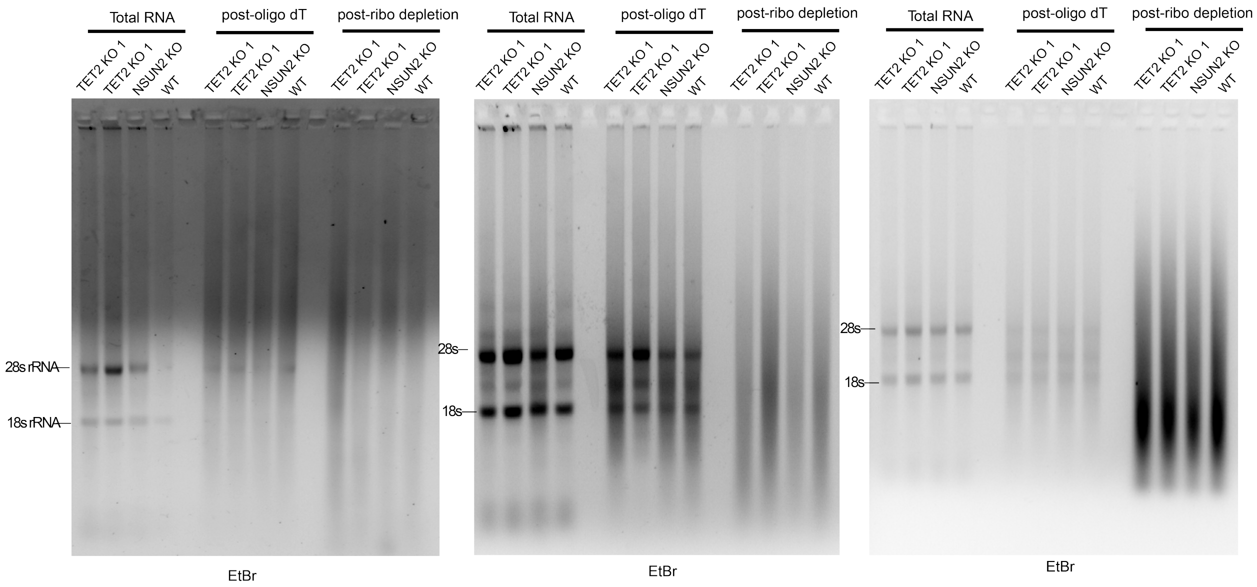

Supplementary Figure 16. Enrichment of mRNA from total mammalian RNA in TET2 and NSUN2 KO Cells (biological triplicates). Extracted total RNA was first subjected to an oligo(dT) pulldown to isolate poly(A)RNA, which was further enriched by depleting ribosomal and small RNA fragments that co-purified during the pulldown. The quality at each step was checked by agarose gel electrophoresis.

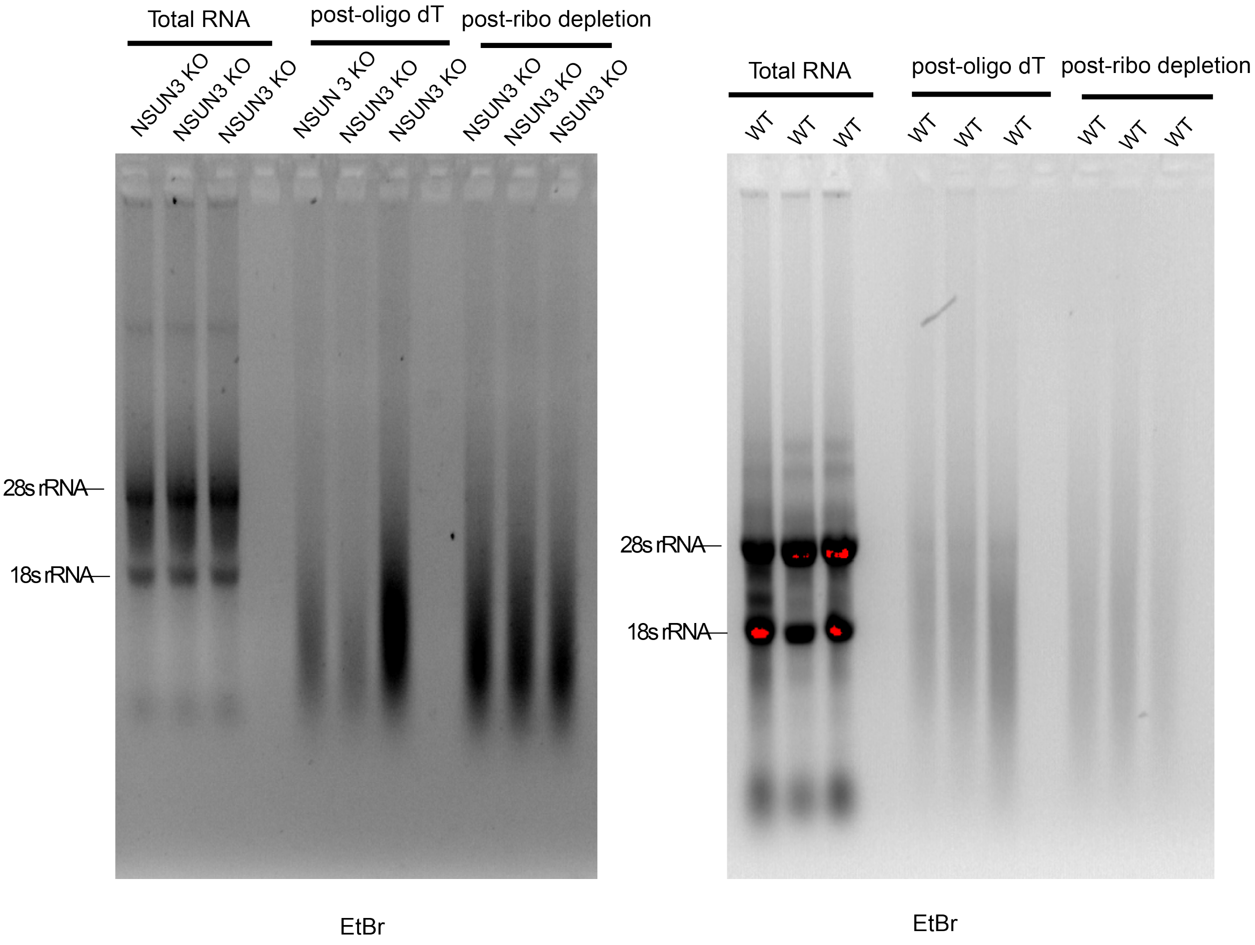

Supplementary Figure 17. Enrichment of mRNA from total mammalian RNA in NSUN3 KO cells (biological triplicates). Extracted total RNA was first subjected to an oligo-dT pulldown to isolate polyA RNA, which was further enriched by depleting ribosomal and small RNA fragments that co-purified during the pulldown. The quality at each step was checked by agarose gel electrophoresis.

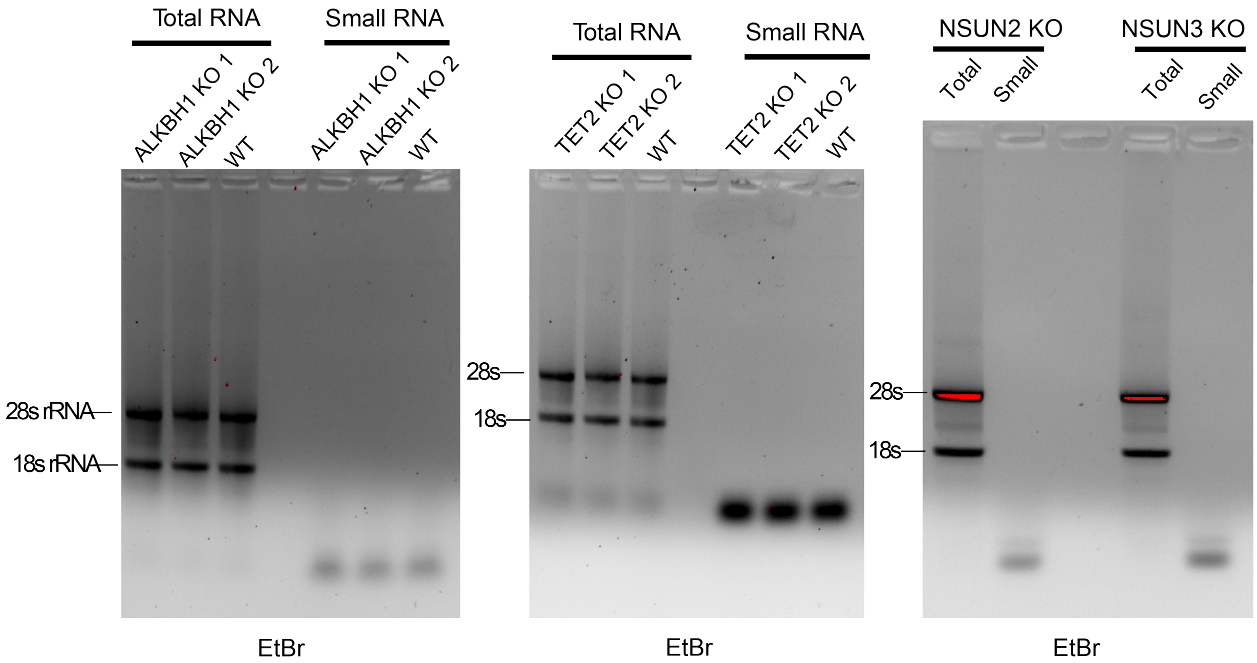

Supplementary Figure 18. Enrichment of small RNA from total mammalian RNA in ALKBH1, NSUN2, NSUN3, and TET2 KO Cells. Extracted total RNA was enriched for small RNA following the protocol for small RNA isolation from Zymo Clean and Concentrator-5. Each extraction was performed in triplicate, with comparable results.

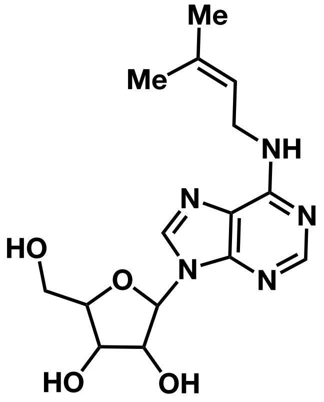

N⁶-isopentenyladenosine (i⁶A)

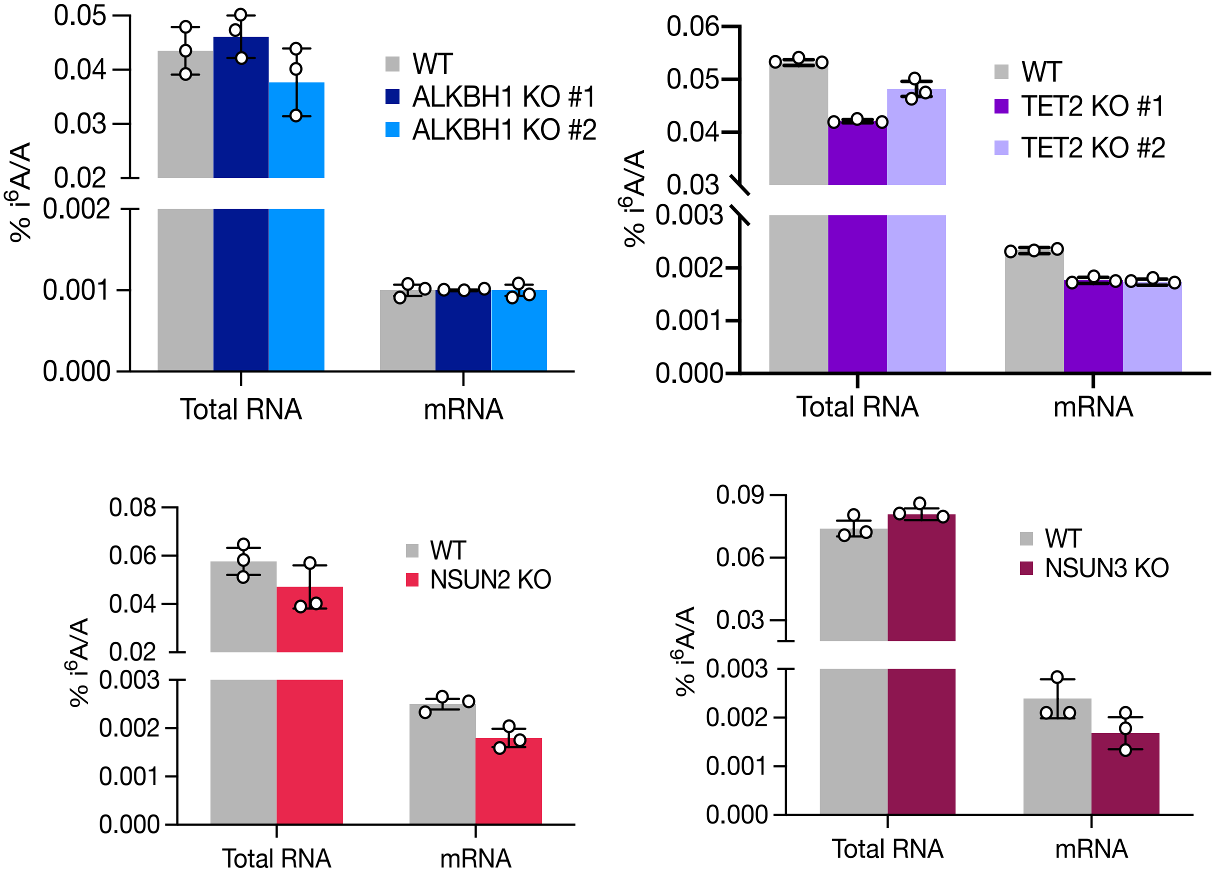

Supplementary Figure 19. Depletion of i^{6}A in mammalian mRNA from WT 293T and ALKBH1/TET2/NSUN2/NSUN3 KO cells. RNA samples were extracted and processed as in Supplementary Fig. 10, digested, dephosphorylated, and analyzed by LC-QQQ-MS. Levels in total and mRNA are consistent with literature reports.[1] i^{6}A is present in mRNA at levels at least 20-fold lower than in total RNA. Three independent biological replicates were analyzed. Data represent mean values ± s.d. Source data are provided as a Source Data file.

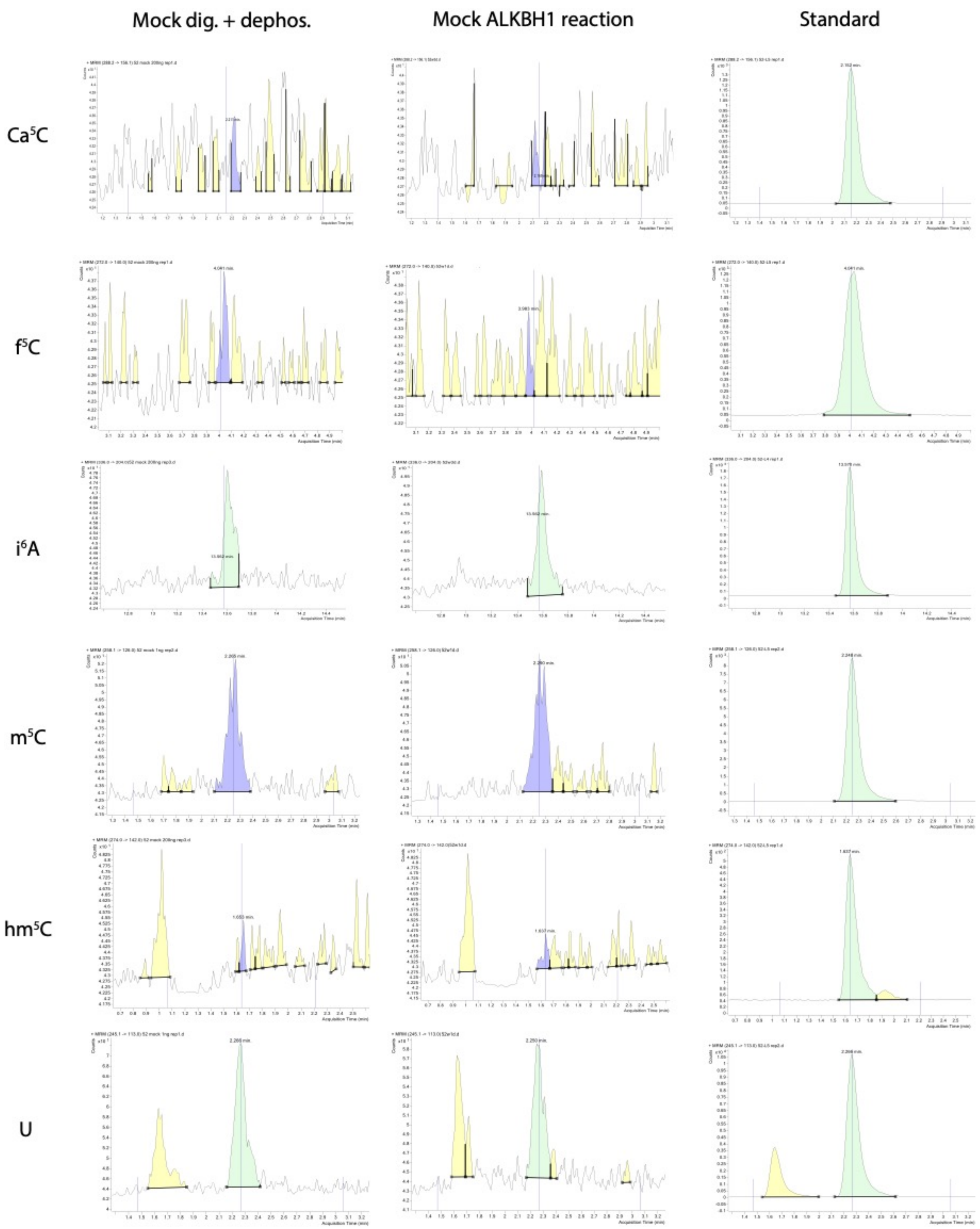

Supplementary Figure 20. Levels of several modified and unmodified nucleosides that arise from mock (no RNA) enzymatic digestion and dephosphorylation (left column) or ALKBH1 in vitro oxidation (middle column). Chromatographic traces for the standards are included for reference (right column). In all cases, nucleoside levels are negligible or completely inexistent.

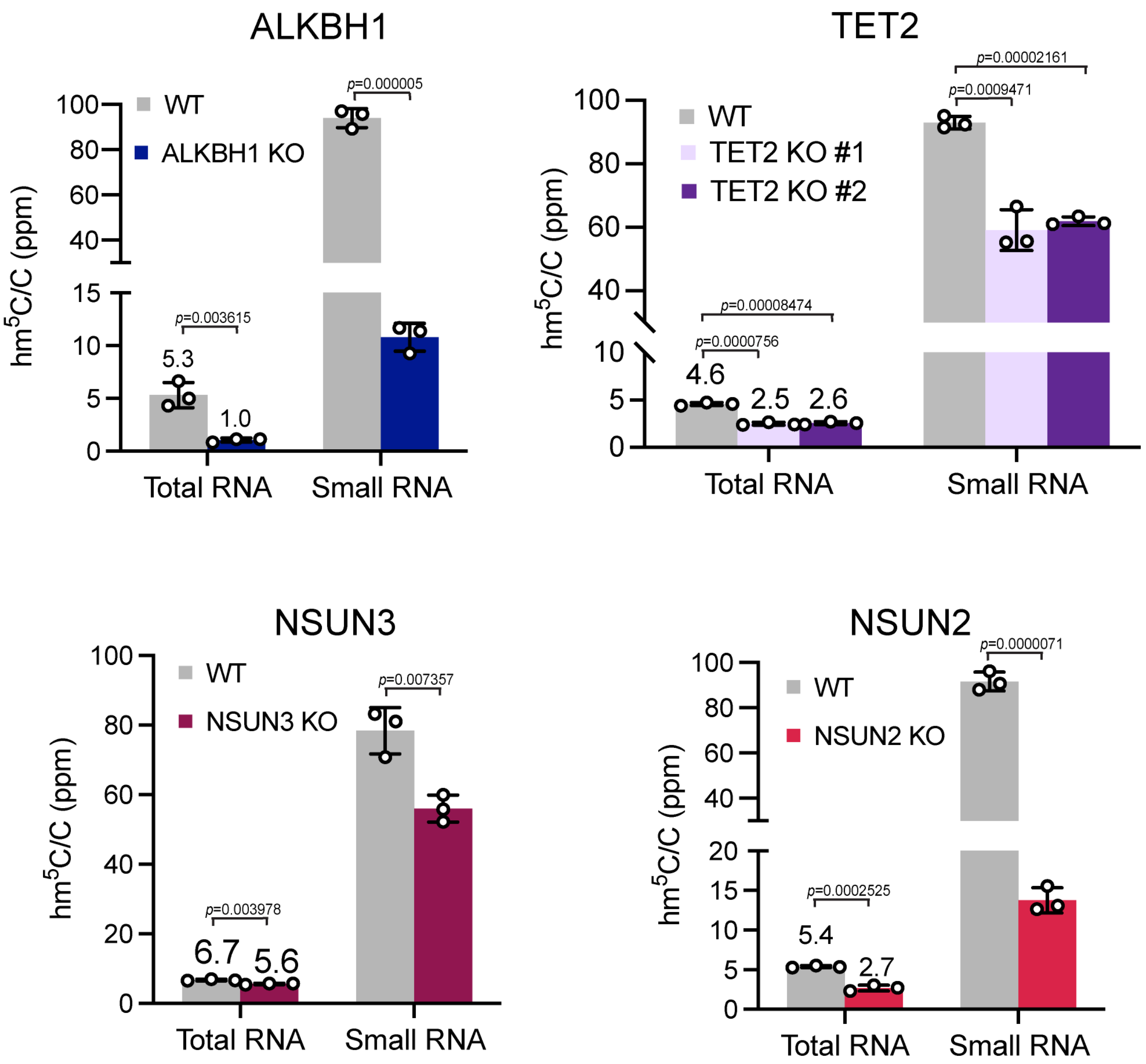

Supplementary Figure 21. Quantification of hm^{5}C levels in total and small RNA of WT 293T cells and various knockouts. Three independent biological replicates were analyzed, and data represent mean values ± s.d. An unpaired t-test (two-tailed) was used to measure the statistical significance *$p < 0.05$, ** $p < 0.01$, *** $p < 0.001$, **** $p < 0.0001$. Source data are provided as a Source Data file.

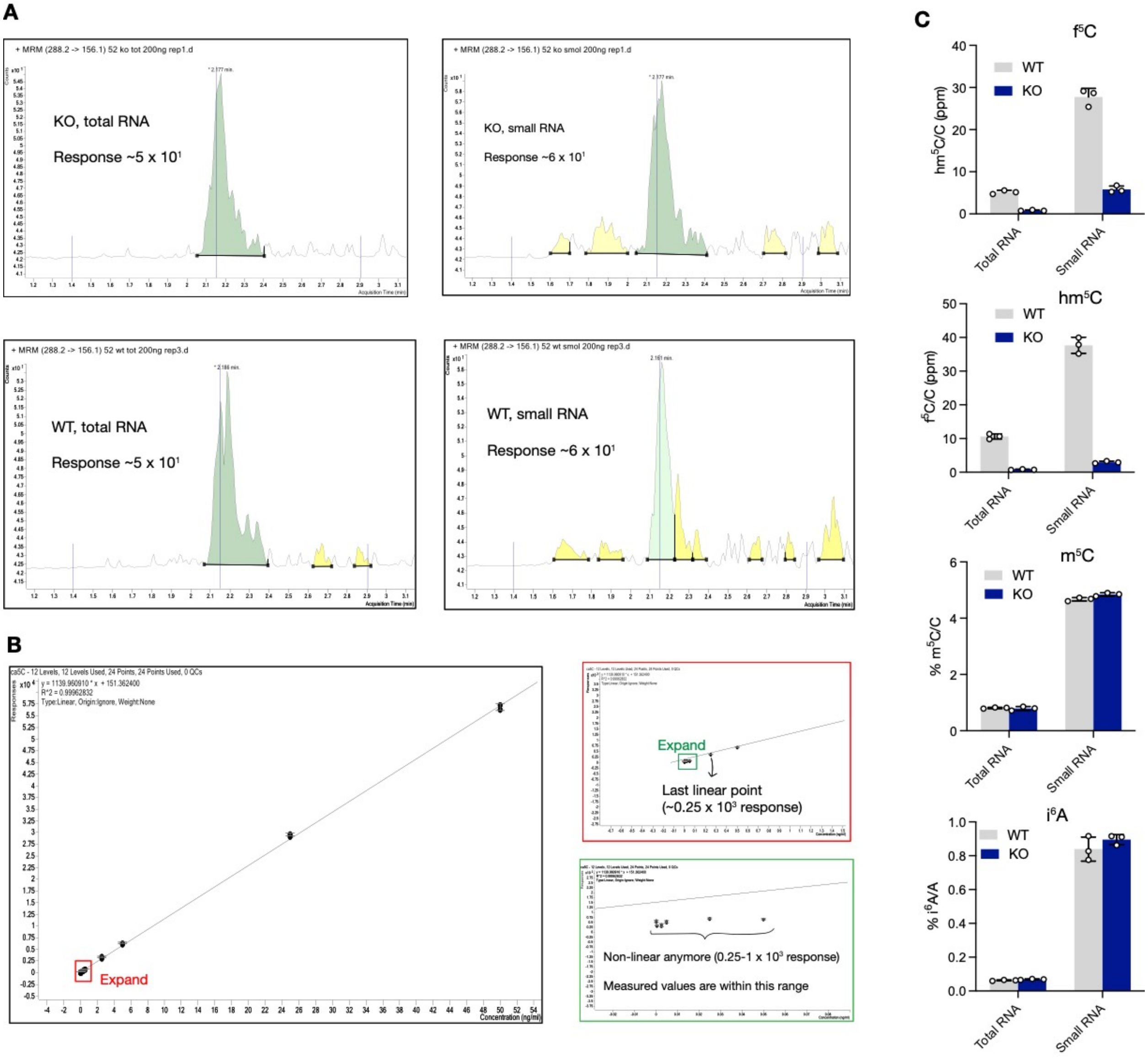

Supplementary Figure 22. 5-carboxycytidine (ca^{5}C) is not formed in cells by ALKBH1. **(A)** Raw signals for ca^{5}C in representative WT and ALKBH1 total and small RNA samples (200 ng) as quantified by LC-MS/MS. **(B)** Calibration curve for ca^{5}C ranging from 50 to 0.00025 ng/mL. Portions of the curve are expanded to highlight the non-linear nature below 0.25 ng/mL. **(C)** Levels of other modifications (f^{5}C, hm^{5}C, i^{6}A, m^{5}C) in the same samples remain consistent with values reported in this and other manuscripts. Three independent biological replicates were analyzed. Data represent mean values ± s.d. Source data are provided as a Source Data file.

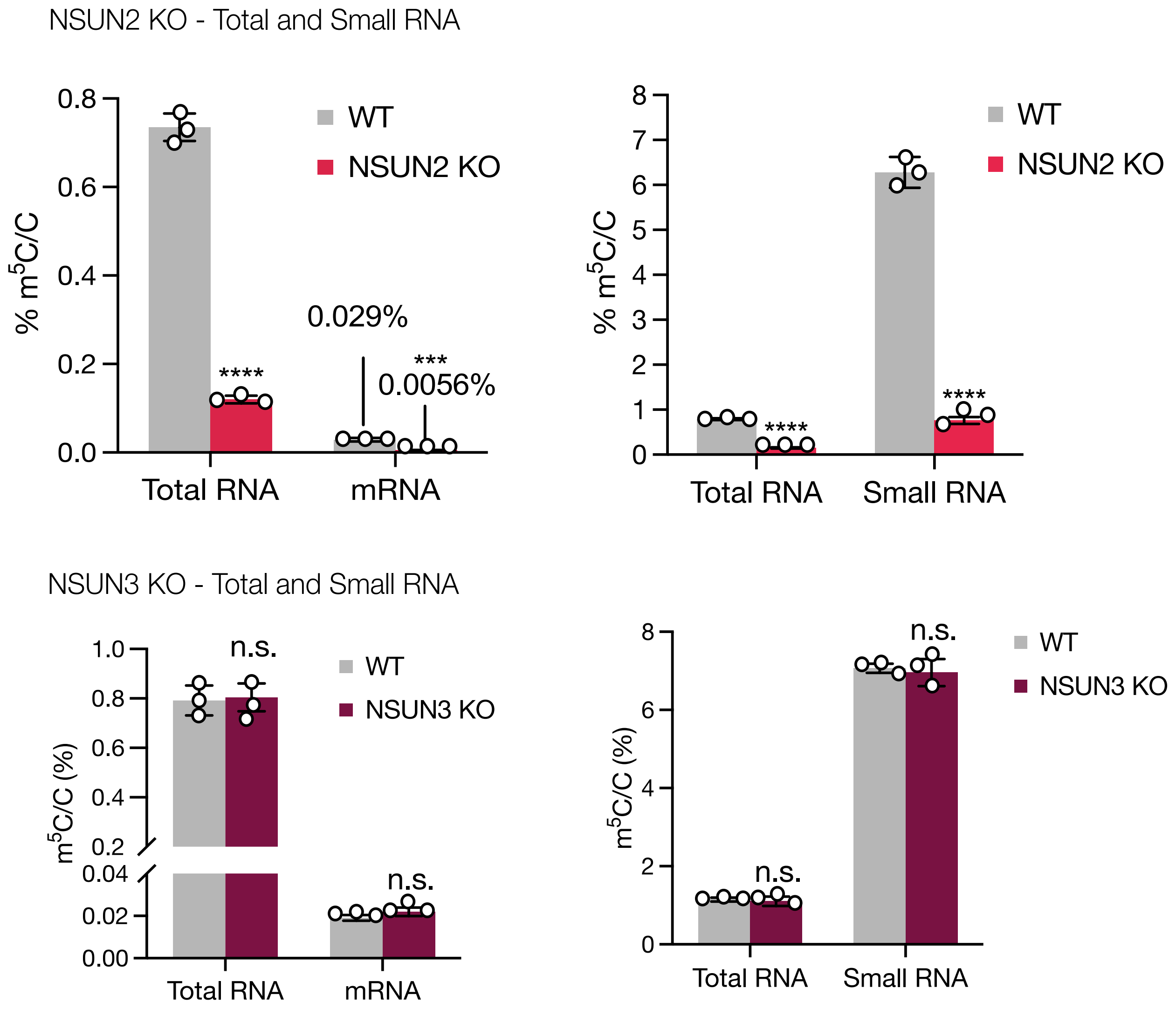

Supplementary Figure 23. Levels of m^5C in total RNA, small RNA, and mRNA from WT 293T and NSUN2/NSUN3 KO cells analyzed by LC-QQQ-MS. Experiment was performed in triplicate. Three independent biological replicates were analyzed. Data represent mean values ± s.d. An unpaired t-test (two-tailed) was performed to evaluate the statistical significance. *p* value, 0.0000049 (NSUN2 KO vs WT total RNA);0.00054 (NSUN2 KO vs WT mRNA); 0.0000014 (NSUN2 KO vs total RNA); 0.0000107 (NSUN2 KO vs WT small RNA). Source data are provided as a Source Data file.

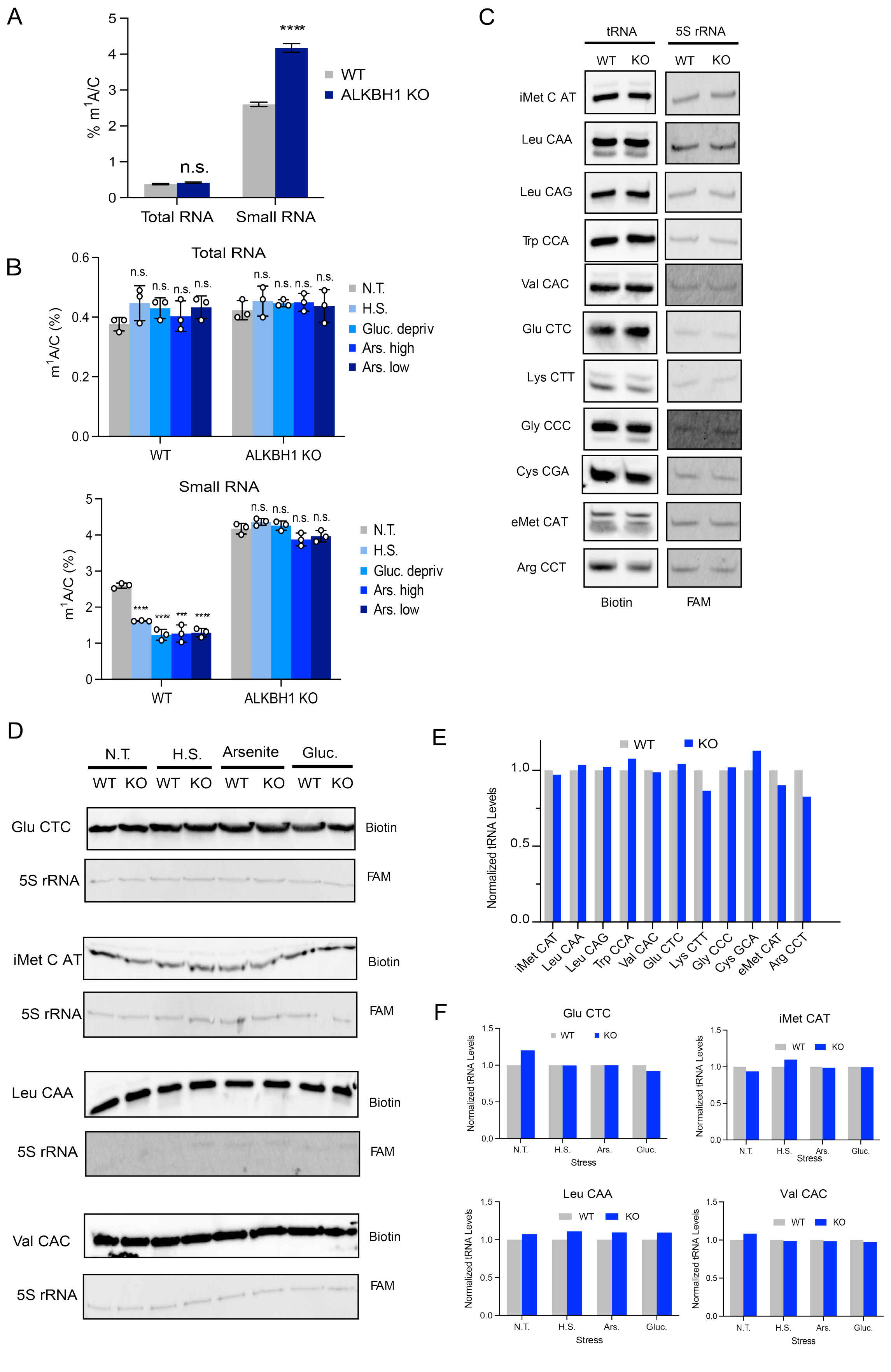
A
% m1A/C
n.s.
****
WT
ALKBH1 KO
Total RNA
Small RNA
B
Total RNA
m1A/C (%)
N.T.
H.S.
Gluc. depriv
Ars. high
Ars. low
WT
ALKBH1 KO
Small RNA
C
tRNA
5S rRNA
WT
KO
iMet C AT
Leu CAA
Leu CAG
Trp CCA
Val CAC
Glu CTC
Lys CTT
Gly CCC
Cys CGA
eMet CAT
Arg CCT
Biotin
FAM
D
N.T.
H.S.
Arsenite
Gluc.
Glu CTC
5S rRNA
iMet C AT
Leu CAA
Val CAC
E
Normalized tRNA Levels
iMet CAT
Leu CAA
Leu CAG
Trp CCA
Val CAC
Glu CTC
Lys CTT
Gly CCC
Cys GCA
eMet CAT
Arg CCT
F
Glu CTC
iMet CAT
Leu CAA
Val CAC
N.T.
H.S.
Ars.
Gluc.
Stress

Supplementary Figure 24. The impact of cellular stress in ALKBH1-dependent levels of m^1A. **(A)** Quantification of m^1A levels in total and small RNA from WT and ALKBH1 KO cell lines. *p* value, 0.7048 (ALKBH1 KO vs WT total RNA); 0.000034 (ALKBH1 KO vs WT small RNA). **(B)** Quantification of m^1A levels in total and small RNA from WT and ALKBH1 KO cell lines under cellular stress; N.T., no treatment; H.S., heat shock (42 ^{o}C for 1 hour); Gluc. depriv, glucose deprivation (5 mM glucose for 8 hours); Ars. high, oxidative stress (0.5 mM $NaAsO_2$ for 1 hour); Ars. low, oxidative stress (0.2 mM $NaAsO_2$ for 4 hour). *p* value**,** 0.000002 (H.S vs N.T.);0.000001(Gluc.depriv vs N.T.); 0.000012 (Ars.high vs N.T.) ; 0.000002 (Ars.low vs N.T). **(C)** Relative levels of various tRNAs in WT or ALKBH1 KO cells. Detection of 5S RNA was used as a loading control. **(D)** Relative levels of various tRNAs in WT or ALKBH1 KO cells under various cellular stresses. Detection of 5S RNA was used as a loading control. **(E)** Quantification of relative levels of various tRNAs from part C. Quantification was done by densitometry in the ImageJ software, and the intensity of each band was normalized to that of the corresponding 5S RNA band. KO values are plotted as a ratio of WT values. **(F)** Quantification of relative levels of various tRNAs from part D. Quantification was done by densitometry in the ImageJ software, and the intensity of each band was normalized to that of the corresponding 5S RNA band. KO values are plotted as a ratio of WT values. For A and B, three independent biological replicates were analyzed, and data represent mean values ± s.d. An unpaired t-test (two-tailed) was used to measure the statistical significance *$p < 0.05$, ** $p < 0.01$, *** $p < 0.001$, **** $p < 0.0001$. Source data are provided as a Source Data file.

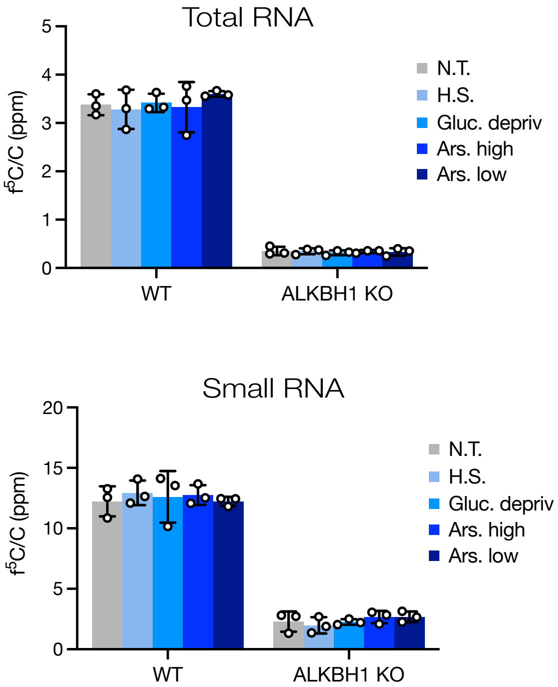

Supplementary Figure 25. Quantification of f^5C levels in total and small RNA from WT and ALKBH1 KO cell lines under cellular stress; N.T., no treatment; H.S., heat shock (42 ^oC for 1 hour); Gluc. depriv, glucose deprivation (5 mM glucose for 8 hours); Ars. high, oxidative stress (0.5 mM $NaAsO_2$ for 1 hour); Ars. low, oxidative stress (0.2 mM $NaAsO_2$ for 4 hour). Three independent biological replicates were analyzed, and data represent mean values ± s.d. An unpaired t-test (two-tailed) was used to measure the statistical significance. No changes were identified as statistically significant. Source data are provided as a Source Data file.

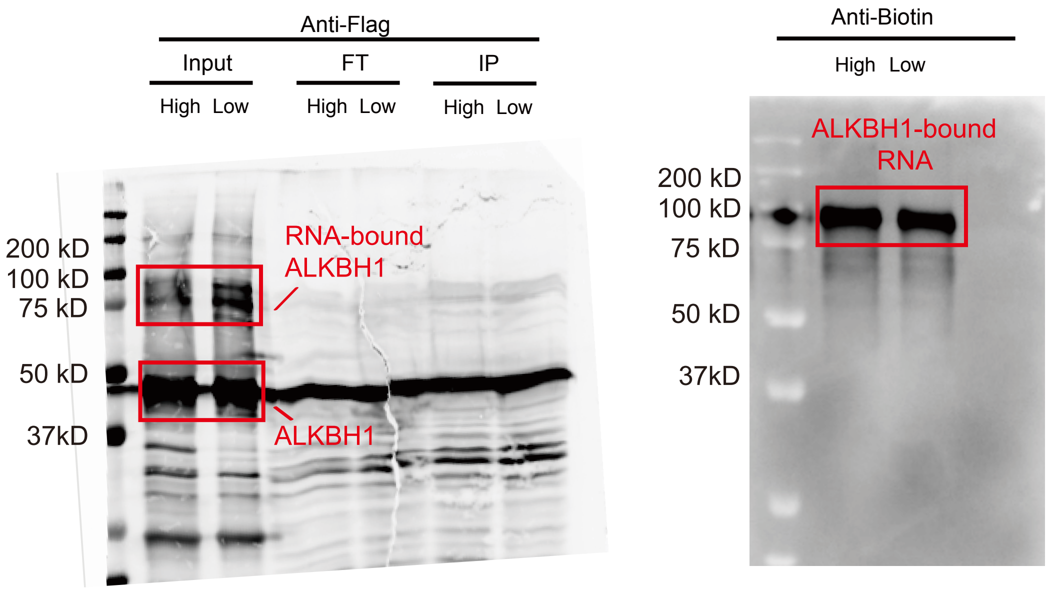

Supplementary Figure 26. 5-EC-iCLIP western blot analysis of 3xFLAG-ALKBH1 immunoprecipitation (IP). Expression of 3xFLAG-ALKBH1 was induced in stable cell lines. Cells were treated with 1mM 5-EC overnight, and RNA-protein crosslinks were partially digested with high and low amounts of RNase and further enriched by FLAG IP. RNA bound to the enriched protein was then biotinylated (full biotin blot from Fig. 4B in main text). Experiment was performed twice.

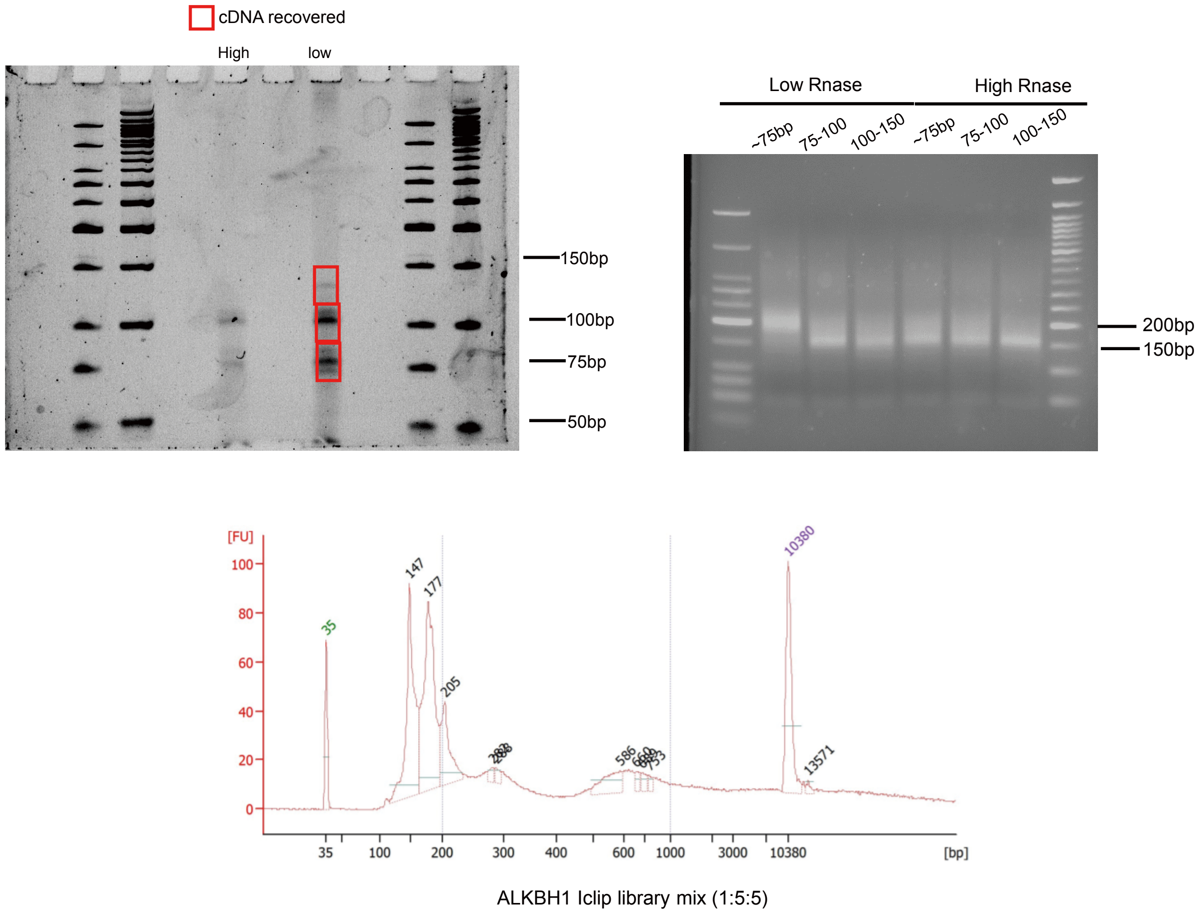

Supplementary Figure 27. 5-EC-iCLIP library preparation for next generation sequencing. Top left: TBE urea gel showing the recovered cDNA. Top right: Agarose gel showing the PCR product of iCLIP. Bottom: bioanalyzer analysis showing the mixed libraries for Illumina sequencing. The experiments were repeated two times independently with similar results.

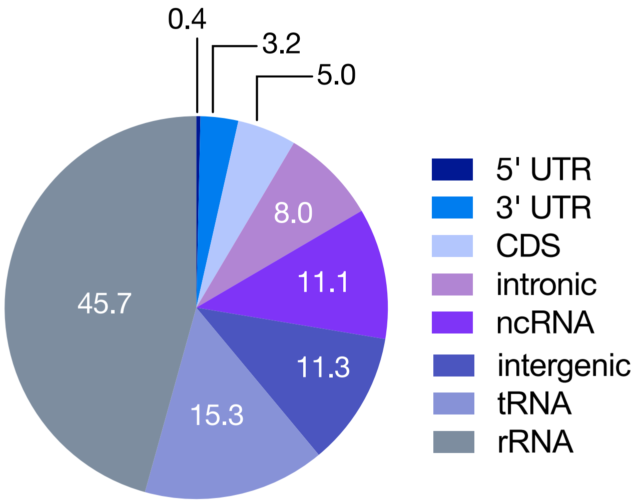

Supplementary Figure 28. RNA composition of untreated control sample (no 5-EC labeling) according to uniquely mapped reads.

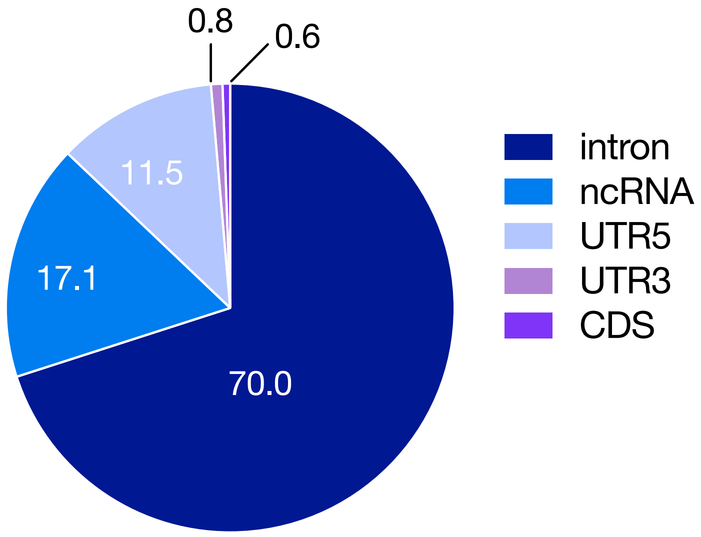

Supplementary Figure 29. Composition of non-tRNA/rRNA identified by 5-EC-iCLIP according to uniquely mapped reads.

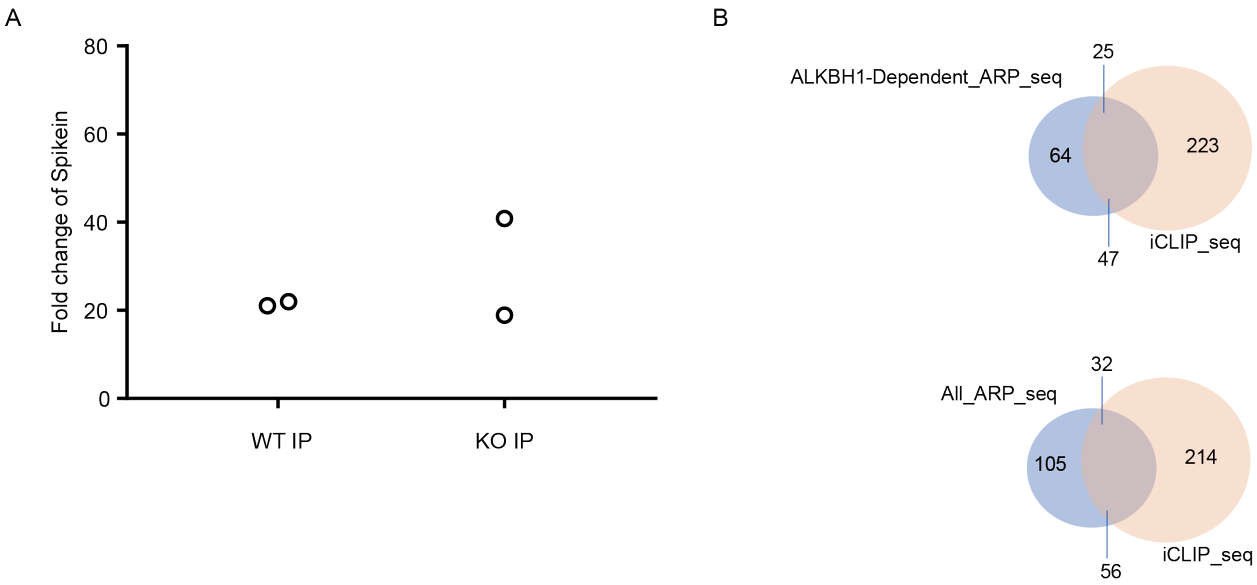

Supplementary Figure 30. ARP sequencing data. (A) Fold enrichment of f^5C-containing spike-in oligo during ARP sequencing in both WT and ALKBH1 KO 293T cells after IP. Two independent biological replicates were performed. Source data are provided as a Source Data file. (B) Venn diagram showing overlap between peaks detected by ARP sequencing and 5-EC-iCLIP. Source data are provided as a Source Data file.

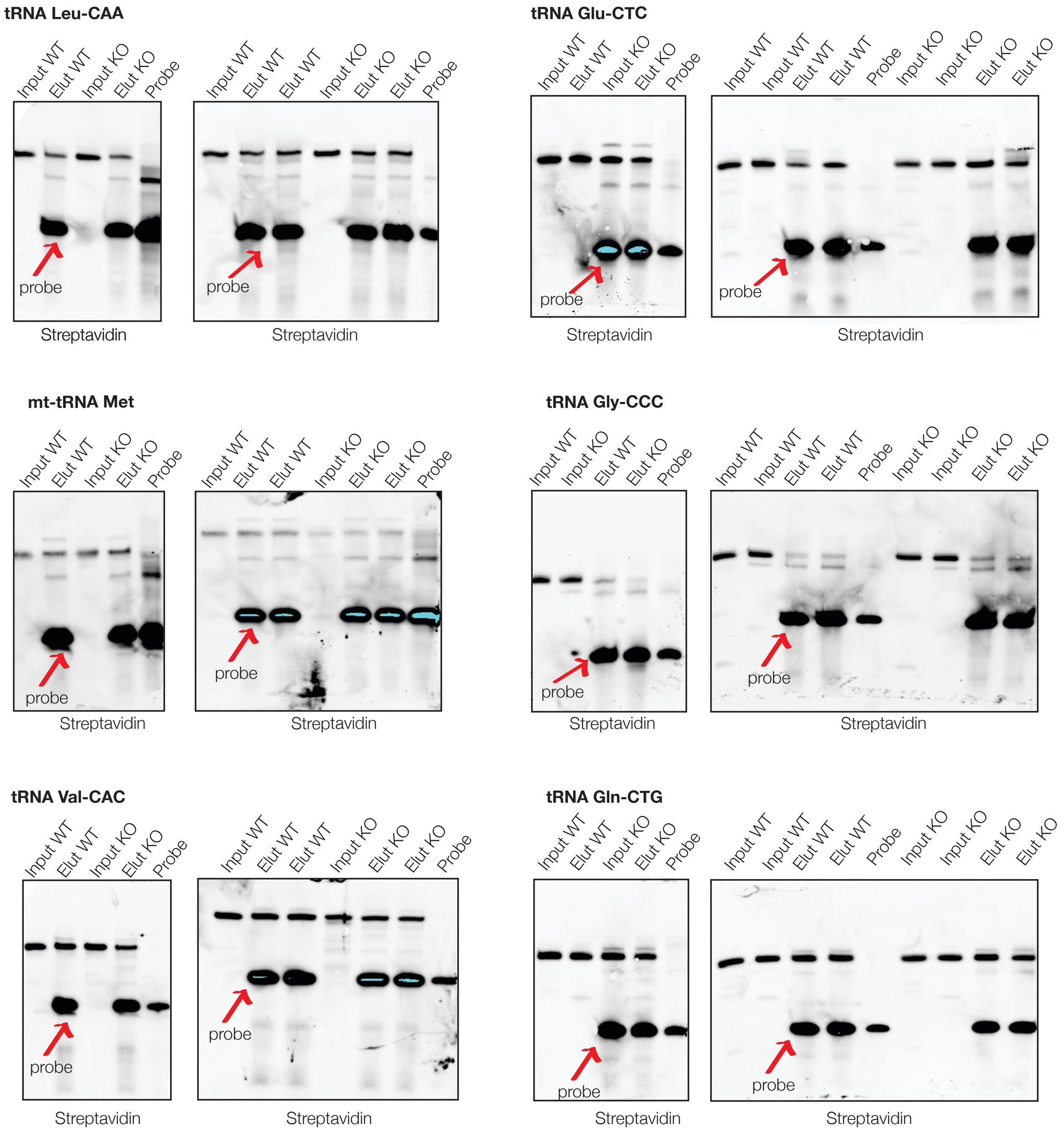

Supplementary Figure 31. Recovery of native tRNAs from WT and ALKBH1 KO cells. Individual tRNAs from bulk small RNA (input) were incubated with a complimentary biotinylated DNA probe, eluted, and analyzed by northern blot. For detection, the membrane-bound tRNA was annealed to the same biotinylated probe and further stained with a streptavidin-conjugated dye. The experiments were repeated three times independently with similar results.

.

Adenosine (A)
500 ng/mL to 5 ng/mL

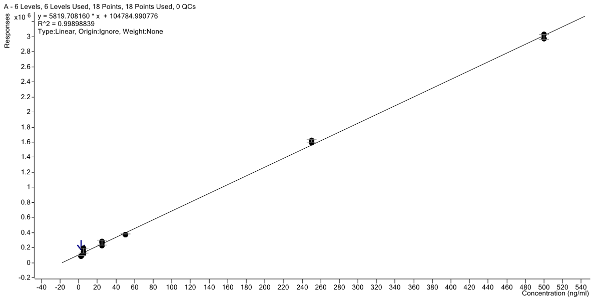

Cytosine (C)
500 ng/mL to 5 ng/mL

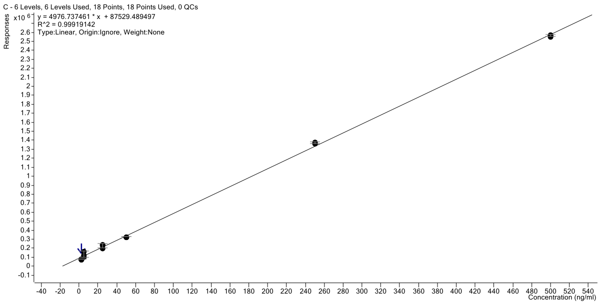

Guanosine (G)
500 ng/mL to 5 ng/mL

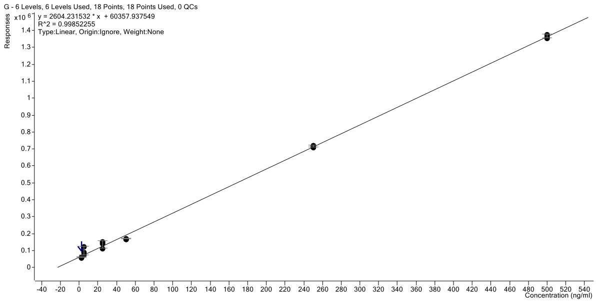

Uridine (U)
500 ng/mL to 5 ng/mL

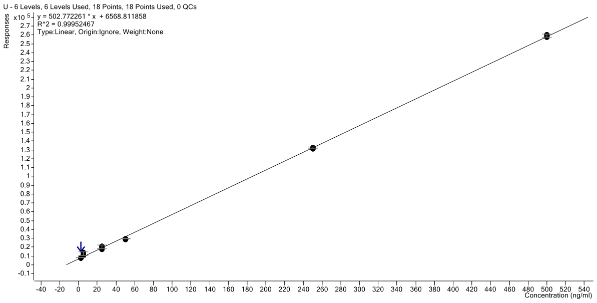

5-methylcytidine (m^5C)
500 ng/mL to 5 ng/mL

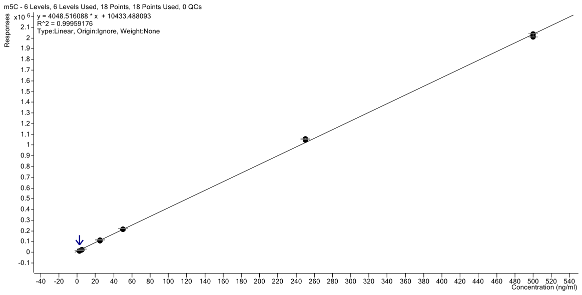

5-formylcytidine (f^5C)
25 ng/mL to 0.25 ng/mL

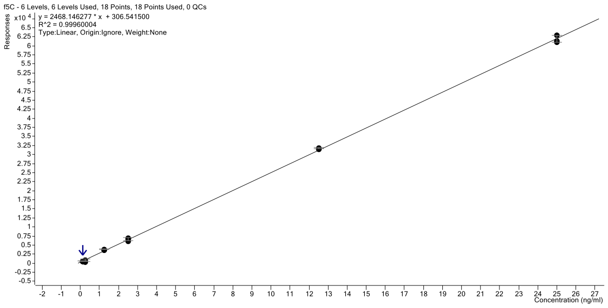

5-hydroxymethylcytidine (hm^5C)
25 ng/mL to 0.25 ng/mL

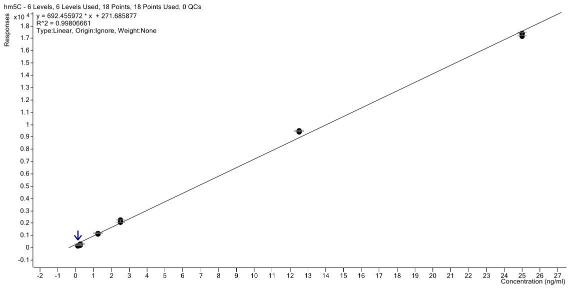

Supplementary Figure 32. Standard curves used for the LC-QQQ-MS quantification of hm^5C and f^5C in synthetic RNA substrates following *in vitro* oxidation with ALKBH1. Two technical replicates for each standard are used to generate standard curves.

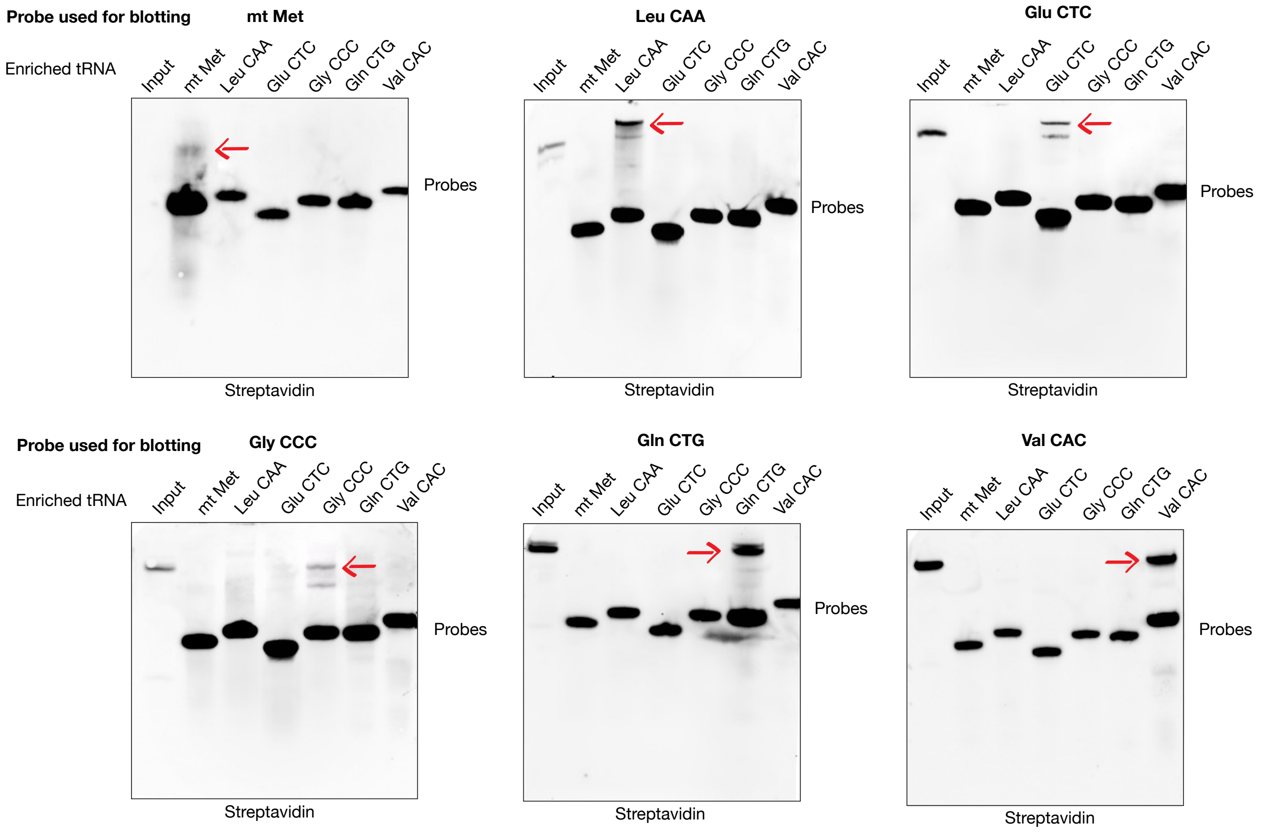

Supplementary Figure 33: tRNA antisense pulldowns with biotinylated probes are specific. Various tRNAs analyzed in this study were enriched by antisense pulldown and analyzed by northern blot using the complementary probe (the one used for pulldown) or a non-complementary probe to assess specificity. The experiments were repeated three times independently with similar results.

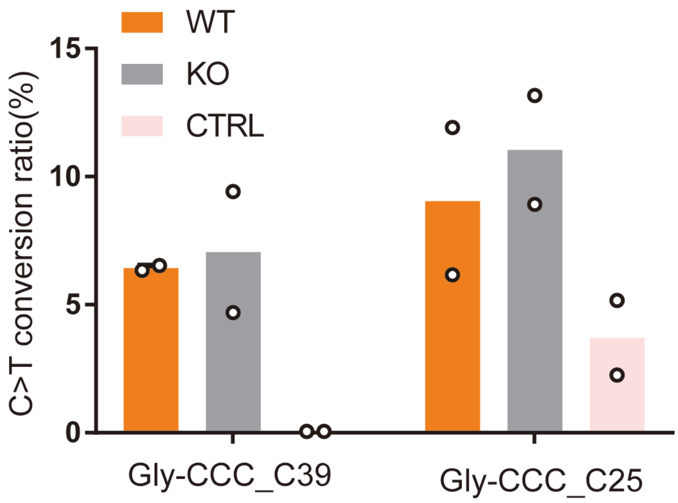

Supplementary Fig 34. ALKBH1-independent f^5C sites in tRNA-Gly-CCC and tRNA-Val-CAC by detected by pyridine borane sequencing. Two independent biological replicates were performed and analyzed. Source data are provided as a Source Data file.

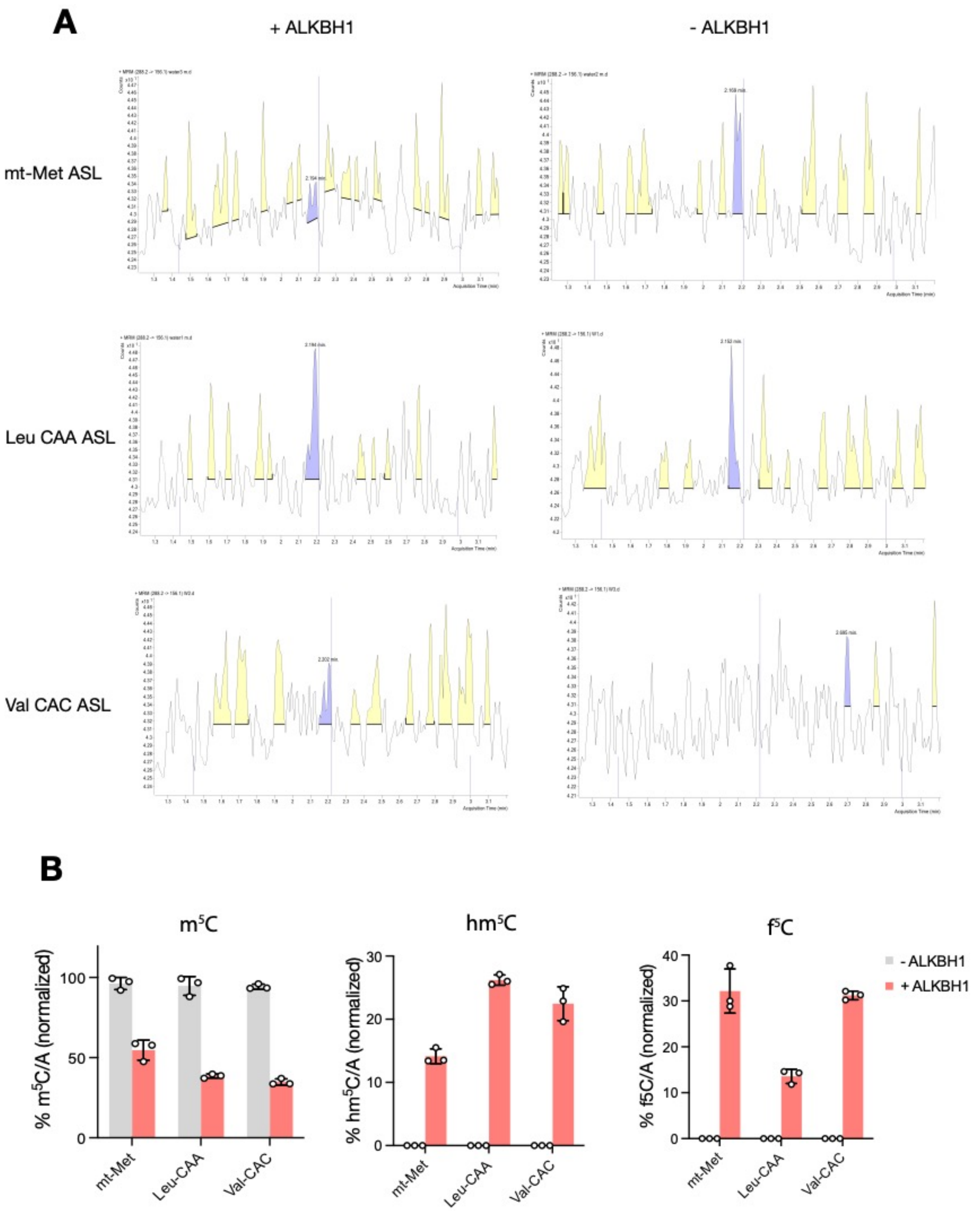

Supplementary Figure 35: ALKBH1 cannot catalyze the formation of ca^{5}C in vitro on model tRNA anticodon stem-loop substrates. **(A)** Raw ca^{5}C LC-MS/MS measurements in select tRNA anticodon stem-loop substrates after oxidative in vitro reaction with or without ALKBH1. Values are negligible. **(B)** Quantification of m^{5}C, hm^{5}C, and f^{5}C for the same samples. Values are similar to those reported in the main text of this manuscript. Three independent biological replicates were analyzed. Data represent mean values ± s.d. Source data are provided as a Source Data file.

Oligo 5:
5’ CAG CGA N7– m^5C – N7 UCG UGC UGC UCG U

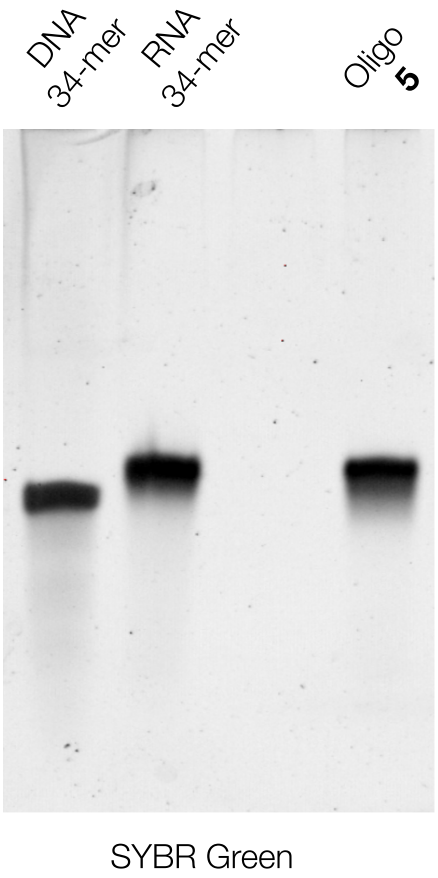

Supplementary Figure 36. Characterization of oligo substrate **5**. Following solid-phase oligonucleotide synthesis and standard cleavage and deprotection, substrate **5** was purified and characterized by denaturing urea-PAGE. The experiments were repeated three times independently with similar results.

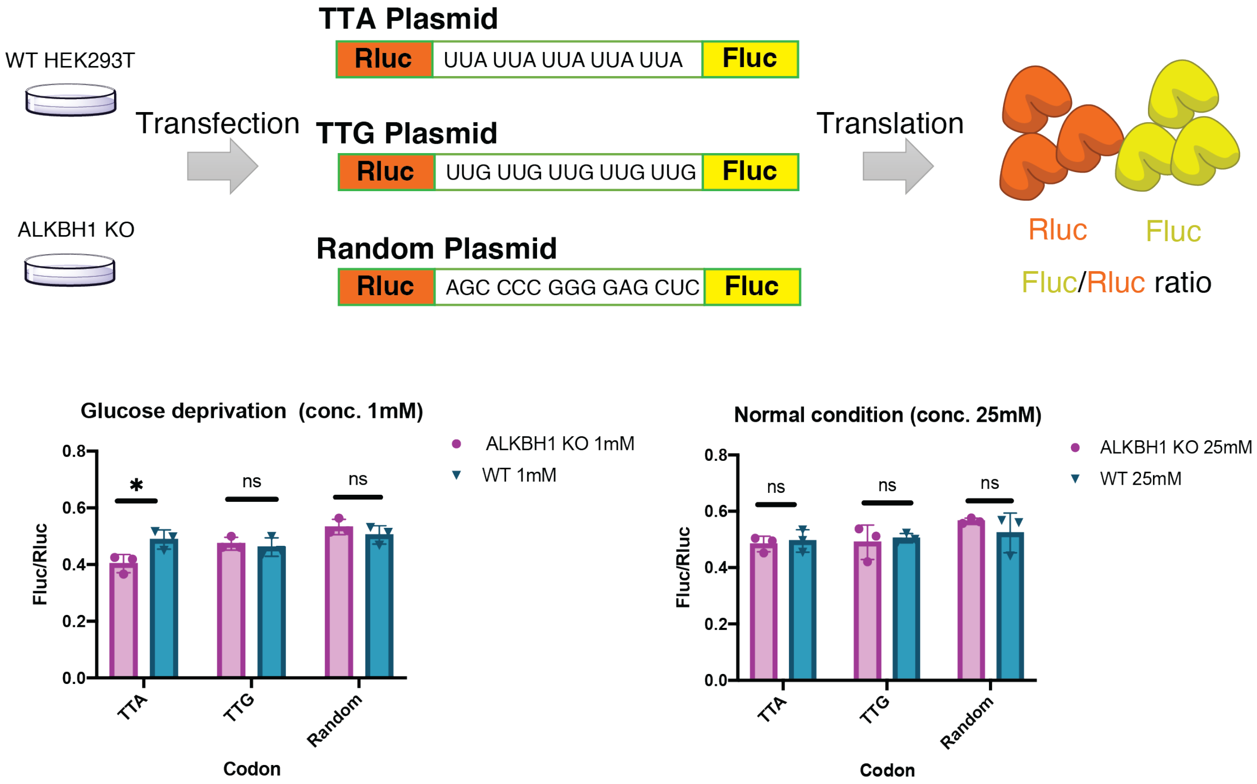

Supplementary Figure 37. Translation of TTG or TTA codons in WT 293T or ALKBH1 KO cells as quantified by dual luciferase assay. Fluc signal is normalized to Rluc signal for each condition. Three independent biological replicates were analyzed, and unpaired two-sided student T test was applied and * as p = 0.034. Source data are provided as a Source data file.

SUPPLEMENTARY TABLES

Supplementary Table 1. Dynamic multiple reaction monitoring (DMRM) parameters of for quantification nucleosides by LC-QQQ-MS.

Nucleoside	Fragmentor Energy (V)	Collision Energy (V)	Parent Ion $[M+H]^+$	Product Ion	Source/Vendor
A	100	20	268.1	136.1	Sigma
i^6A	100	20	336.0	204.0	Cayman
G	80	13	284.1	152.1	Sigma
C	70	14	244.1	112.1	Sigma
m^5C	70	14	258.1	126.1	Carbosynth
hm^5C	70	14	274.0	142.0	Carbosynth
5-EC	70	14	272.0	140.0	synthesized
f^5C	70	14	268.1	136.1	Berry & Associates
U	70	7	245.1	113.1	Sigma
5-EU	70	7	273.0	141.0	Carbosynth
ca^5C	80	14	288.2	156	Carbosynth

Supplementary Table 2. Nucleoside concentration (ng/mL) in total RNA of WT HEK 293T cells treated or untreated with 1 mM 5-EC as measured by LC-QQQ-MS. Experiments were performed in triplicate. Abundant nucleosides (ACGU) were quantified in 1 ng of RNA, while modified nucleosides (5-EC, m^5C) were quantified in 100 ng of sample. Raw values correspond to data of Fig. 1D in main text.

Treatment	Amount	C	U	m^5C	A	G	5-EC	5-EU
5-EC	1 ng	461.12	155.88	497.04	243.14	639.51	624.42	4.030
5-EC	1 ng	478.79	358.90	484.69	222.22	1377.11	602.67	3.974
5-EC	1 ng	423.92	292.21	420.55	420.60	1188.87	632.96	4.397
Control	1 ng	284.51	124.57	224.50	175.79	344.76	0	0
Control	1 ng	285.50	123.63	239.42	170.34	337.26	0	0
Control	1 ng	291.68	126.26	248.37	168.48	335.61	0	0

Supplementary Table 3: f^5C nucleoside concentration (ng/mL) in total RNA of WT 293T cells after cells after overnight treatment with 1mM 5-EC or vehicle as measured by LC-MS/MS. Experiments were performed in triplicate. Nucleosides marked with † were quantified in 200 ng of RNA. All others were quantified in 1 ng of RNA. Raw values correspond to the data of Supplementary Fig. 4.

Treatment	C	m5C	U	5-EC†	A	f5C†	G
DMSO	164.8	1.57	72.9	0	86.9	0.330	246.5
DMSO	156.2	1.59	73.4	0	77.9	0.346	222.7
DMSO	160.9	1.60	77.5	0	82.2	0.350	226.4
5-EC	141.2	1.51	71.6	200.4	73.2	0.301	196.7
5-EC	144.9	1.49	66.4	194.4	71.4	0.327	192.2
5-EC	138.9	1.48	63.3	189.7	66.9	0.304	182.5

Supplementary Table 4. Sequences and characterization of anticodon stem-loop (ASL) substrates and linear RNA oligo substrates **1**-**8**.

Substrate	Sequence	z	Exp. mass	Obs. mass (ESI-TOF HRMS)
Mt-Met ALS	5’ UAU CGG GCC m^5CAU ACC CCG AA	3	2110.97281	2110.97582
iMet-CAT ASL	5’ UCG UGG GCC m^5CAU AAC CCA GA	3	2124.30819	2124.3133
Val-CAC ASL	5’ CGU UCG CCU m^5CAC ACG CGA AA	3	2110.97281	2110.96902
Leu-CAA ASL	5’ CGC CAG ACU m^5CAA GUU CUG GU	3	2216.96045	2116.94294
Gln-CTG ASL	5’ CUC UGG ACU m^5CUG AAU CCA GC	4	1577.46875	1577.46582
Glu-CTC ASL	5’ UUC GGC GCU m^5CUC ACC GCC GC	4	1569.21602	1559.20509
Gly-CCC ASL	5’ UUC UCG CCU m^5CCC ACG CGG GA	4	1575.21875	1575.21534
Leu-CAG ASL	5’ CGC UGC GUU m^5CAG GUC GCA GU	4	1595.46775	1594.46023
1	5’ Bio-UAU CGG GCC (5-EC)AU ACC CCG AA	4	1686.76288	1686.75218
2	5’ Bio-UAU CGG GCC (m^5C)AU ACC CCG AA	4	1684.26678	1684.2564
3	5’ UAU AGA GCC m^5CAU ACC CCG AA	3	2113.64491	2113.64849
4	5’ UAU UGU GCC m^5CAU ACC CCG AA	3	2098.29343	2098.29488
5	5’ CAG CGA NNN NNN Nm^5CN NNN NNN UCG UGC UGC UCG U	n/a	N/A (library)	N/A (library)
6	5’ UUU GAA m^5CCC GG	3	1158.50233	1158.50818
7	5’ UUU GAA Cm^5CC GG	3	1158.50233	1158.49967
8	5’ UUU GAA CCm^5C GG	3	1158.50233	1158.49185

Supplementary Table 5. Sequences of guide RNAs for CRISPR-Cas9 KO cell generation.

KO	Sequence
ALKBH1 KO1/2	TTCGGAAACTTTTCCGCTTC
TET2 KO1	TGGAGAAAGACGTAACTTCG
TET2 KO2	ATCGCATACCTCAGGGCAGA
NSUN2	GCTGCGGATTGCAACACGCG
NSUN3	TGTTCAAATGATCGAAGCTG

Supplementary Table 6. Nucleoside concentration (ng/mL) in total RNA and mRNA of WT HEK 293T and ALKBH1 KO cells measured by LC-QQQ-MS. Experiments were performed in triplicate. Nucleosides marked with † were quantified in 200 ng of RNA. All others were quantified in 1 ng of RNA. Raw values correspond to data of Figs. 3B-C in main text.

Cell Line	RNA type	$hm^5C^†$	C	U	m^5C	A	$f^5C^†$	G	i^6A
KO 1	Total	0.0133	175.7	85.0	1.5134	134.9	0.0202	272.4	0.0732
KO 1	Total	0.0136	172.6	77.4	1.3912	120.4	0.0162	221.0	0.0406
KO 1	Total	0.0212	199.6	92.8	1.7586	182.6	0.0212	330.9	0.0092
KO 1	mRNA	0	161.4	144.4	0.0461	207.1	0.0104	238.7	0.4133†
KO 1	mRNA	0	148.4	128.6	0.0409	191.5	0.0108	216.7	0.3882†
KO 1	mRNA	0	167.4	147.7	0.0393	259.4	0.0138	262.9	0.5325†
KO 2	Total	0.0184	208.3	105.9	1.7324	168.6	0.0408	314.5	0.0759
KO 2	Total	0.0171	184.7	89.6	1.5825	144.6	0.0289	290.2	0.0554
KO 2	Total	0.0242	212.6	98.2	1.7686	195.4	0.0495	353.1	0.0144
KO 2	mRNA	0	157.8	139.5	0.0304	193.7	0.0116	231.1	0.4362†
KO 2	mRNA	0	147.8	126.8	0.0401	187.4	0.0108	210.5	0.4062†
KO 2	mRNA	0	150.1	134.5	0.0324	232.5	0.0177	238.0	0.5766†
WT	Total	0.1463	240.3	119.9	1.9143	188.7	0.4143	363.0	0.0921
WT	Total	0.1262	209.0	94.3	1.9961	161.4	0.351	322.4	0.0615
WT	Total	0.151	224.5	105.1	1.9910	205.7	0.346	371.5	0.0282
WT	mRNA	0	144.1	123.6	0.0374	174.2	0.0505	211.7	0.2661†
WT	mRNA	0	145.5	122.2	0.0355	184.2	0.049	215.4	0.2554†
WT	mRNA	0	147.8	130.1	0.0323	214.8	0.0521	236.0	0.2185†

Supplementary Table 7. Nucleoside concentration (ng/mL) in total and small RNA of WT HEK 293T and ALKBH1 KO cells measured by LC-QQQ-MS. Experiments were performed in triplicate. Nucleosides marked with † were quantified in 200 ng of RNA. All others were quantified in 1 ng of RNA. Raw values correspond to data of Figs. 3B-D in main text.

Cell Line	RNA type	hm^5C†	C	U	m^5C	A	f^5C†	G	i^6A†
KO 1	Total	0.041	360.32	185.97	2.512	317.90	0.0388	567.02	17.584
KO 1	Total	0.0298	360.53	186.40	2.918	320.32	0.0346	569.92	17.662
KO 1	Total	0.0414	363.72	188.45	2.741	322.92	0.0300	574.01	17.356
KO 1	small	0.3026	236.67	206.94	19.741	345.93	0.0809	552.35	144.518
KO 1	small	0.3026	329.44	205.11	15.748	346.30	0.0815	555.84	155.959
KO 1	small	0.3746	323.96	208.55	18.201	346.44	0.0974	554.89	154.429
WT	Total	0.2052	411.68	214.78	3.336	357.05	0.4510	639.57	14.235
WT	Total	0.272	409.78	212.66	3.252	355.78	0.3839	645.67	13.862
WT	Total	0.1349	407.50	210.32	2.937	356.26	0.4932	643.97	14.265
WT	small	3.0133	314.98	191.44	17.782	324.69	1.3855	527.66	144.514
WT	small	3.2078	330.60	196.94	17.297	177.58	1.3854	356.54	98.065
WT	small	2.9223	327.72	194.30	19.971	159.61	1.4053	364.29	86.706

Supplementary Table 8. Nucleoside concentration (ng/mL) in total RNA and mRNA of WT HEK 293T and TET2 KO cells measured by LC-QQQ-MS. Experiments were performed in triplicate. Nucleosides marked with † were quantified in 200 ng of RNA. All others were quantified in 1 ng of RNA. Raw values correspond to data of Fig. 3E in the main text.

Cell Line	RNA type	hm^{5}C†	C	U	m^{5}C†	A	f^{5}C†	G	i^{6}A†
KO 1	Total	0.0375	178.1	85.3	271.3	132.4	0.423	255.5	14.111
KO 1	Total	0.0325	164.3	79.4	266.5	133.7	0.408	273.8	14.078
KO 1	Total	0.0356	164.7	80.2	266.7	130.9	0.390	263.9	14.049
KO 1	mRNA	0	157.4	126.4	4.87	182.8	0.077	226.6	0.856
KO 1	mRNA	0	157.0	125.2	4.78	180.6	0.074	222.9	0.833
KO 1	mRNA	0	151.1	126.0	4.73	181.4	0.084	226.5	0.842
KO 2	Total	0.0254	178.6	86.7	293.6	147.4	0.289	286.8	12.410
KO 2	Total	0.0326	179.6	87.0	285.2	145.3	0.301	281.7	12.336
KO 2	Total	0.0401	179.4	88.5	286.8	146.7	0.289	281.1	12.268
KO 2	mRNA	0	142.1	120.8	3.27	173.1	0.040	203.0	0.631
KO 2	mRNA	0	143.7	120.4	3.21	172.8	0.040	200.7	0.629
KO 2	mRNA	0	132.4	116.5	3.20	172.0	0.042	205.1	0.606
WT	Total	0.1463	183.6	83.1	293.4	136.1	0.347	266.5	12.715
WT	Total	0.1203	181.5	83.7	295.5	138.0	0.375	269.6	13.358
WT	Total	0.1238	184.1	83.4	291.1	136.6	0.361	264.9	13.513
WT	mRNA	0	138.8	111.0	3.79	159.9	0.057	192.3	0.567
WT	mRNA	0	123.8	107.3	3.72	159.8	0.055	195.0	0.540
WT	mRNA	0	128.6	109.0	3.83	162.5	0.056	197.0	0.560

Supplementary Table 9. Nucleoside concentration (ng/mL) in total RNA and small of WT HEK 293T and TET2 KO cells measured by LC-QQQ-MS. Experiments were performed in triplicate. Nucleosides marked with † were quantified in 200 ng of RNA. All others were quantified in 1 ng of RNA.

Cell Line	RNA Type	hm5C†	C	m5C	U	A	f5C†	G	i6A†
KO1	Total	0.061	124.8	1.25	74.4	186.3	0.277	211.0	15.4
KO1	Total	0.061	127.3	1.21	75.9	185.4	0.268	209.5	15.4
KO1	Total	0.062	115.9	1.03	68.8	181.4	0.260	187.5	15.6
KO2	Total	0.073	149.4	1.48	86.2	171.2	0.318	229.3	18.1
KO2	Total	0.079	145.4	1.35	86.0	173.9	0.315	221.3	16.6
KO2	Total	0.061	119.6	1.19	68.6	172.3	0.304	190.4	14.6
WT	Total	0.114	129.1	1.31	78.7	180.5	0.306	172.6	15.5
WT	Total	0.138	150.4	1.22	89.5	192.0	0.325	209.6	20.5
WT	Total	0.126	133.5	1.36	78.7	182.7	0.301	180.5	18.6
KO1	Small	1.34	120.5	6.91	79.0	146.9	0.989	184.1	127.4
KO1	Small	1.66	125.0	7.59	75.7	146.5	0.988	183.7	141.2
KO1	Small	1.57	142.2	7.78	85.2	137.9	1.20	193.7	117.0
KO2	Small	1.47	120.8	8.11	71.2	142.2	1.09	171.5	168.4
KO2	Small	1.51	123.0	8.30	74.2	129.1	0.98	183.0	149.0
KO2	Small	1.53	120.5	8.17	72.6	139.7	1.05	190.6	165.3
WT	Small	2.29	120.5	8.19	73.9	136.1	1.08	209.3	157.7
WT	Small	2.55	139.7	9.21	87.0	173.0	1.19	220.5	183.9
WT	Small	2.56	138.5	9.33	86.8	198.8	1.06	203.9	170.7

Supplementary Table 10. ca^5C nucleoside concentration (ng/mL) in total RNA and small of WT HEK 293T and ALKBH1 measured by LC-QQQ-MS. Experiments were performed in triplicate. Nucleosides marked with † were quantified in 200 ng of RNA. All others were quantified in 1 ng of RNA. Raw values correspond to the data of Supplementary Fig. 22

Cell Line	RNA Type	hm5C†	C	ca5C†	m5C	U	A	f5C†	G	i6A†
KO	Total	0.0321	170.7	0	1.47	81.7	115.9	0.0303	199.7	15.1
KO	Total	0.0318	216.7	0	1.56	101.7	104.2	0.0289	239.7	14.4
KO	Total	0.0321	212.3	0	1.69	102.7	102.2	0.03184	232.3	14.5
KO	Small	0.2964	219.7	0	10.67	115.1	112.3	0.1265	236.7	193.8
KO	Small	0.1725	164.5	0	7.86	85.4	76.0	0.109	167.1	138.4
KO	Small	0.2082	190.3	0	9.32	97.9	97.2	0.1011	202.3	177.8
WT	Total	0.264	255.7	0	2.01	120.3	100.4	0.502	249.1	12.0
WT	Total	0.2207	233.3	0	1.90	108.5	93.9	0.5306	231.9	11.9
WT	Total	0.2742	245.4	0	2.03	116.3	98.2	0.5224	247.5	12.8
WT	Small	1.171	200.7	0	9.41	107.1	105.1	1.5192	181.2	173.7
WT	Small	1.028	202.2	0	9.56	106.8	102.7	1.6159	193.1	188.2
WT	Small	1.1461	199.7	0	9.20	105.0	92.4	1.4049	198.4	143.2

Supplementary Table 11. Nucleoside concentration (ng/mL) in total RNA and mRNA of WT HEK 293T and NSUN2 KO cells measured by LC-QQQ-MS. Experiments were performed in triplicate. Nucleosides marked with † were quantified in 200 ng of RNA. All others were quantified in 1 ng of RNA. Raw values correspond to data of Fig. 3F in the main text.

Cell Line	RNA Type	5hmC†	C	U	m5C†	A	f5C†	G	i6A†
KO	Total	0.0252	42.59	12.17	7.27	30.27	0.073	46.61	2.49
KO	Total	0.0127	36.83	14.05	7.34	32.54	0.084	52.47	2.63
KO	Total	0.0137	44.22	12.31	7.41	22.98	0.098	45.19	2.74
KO	mRNA	0	133.3	95.84	1.59	46.28	0.061	71.17	0.13
KO	mRNA	0	134.5	95.86	1.48	54.66	0.047	83.4	0.18
KO	mRNA	0	129.0	92.67	1.37	4.99	0.050	17.87	0.025
WT	Total	0.0231	46.69	14.67	59.42	17.07	0.125	46.49	1.78
WT	Total	0.0217	53.02	12.61	56.93	16.33	0.107	44.63	2.13
WT	Total	0.0174	28.36	14.41	64.97	14.23	0.138	42.70	1.57
WT	mRNA	0	126.3	104.9	6.02	33.04	0.070	67.43	0.089
WT	mRNA	0	79.65	61.74	5.14	19.88	0.071	40.89	0.090
WT	mRNA	0	87.09	57.68	5.20	11.56	0.073	30.24	0.090

Supplementary Table 12. Nucleoside concentration (ng/mL) in total and small RNA of WT HEK 293T and NSUN2 KO cells measured by LC-QQQ-MS. Experiments were performed in triplicate. Nucleosides marked with † were quantified in 200 ng of RNA. All others were quantified in 1 ng of RNA. Raw values correspond to data of Fig. 3F in the main text.

Cell Line	RNA type	hm^{5}C†	C	U	m^{5}C	A	f^{5}C†	G	i6A†
KO	Total	0.107	393.82	220.13	0.583	368.66	0.395	758.47	17.917
KO	Total	0.121	398.96	224.96	0.693	360.71	0.462	733.65	16.050
KO	Total	0.094	403.99	227.79	0.569	360.01	0.388	729.19	16.696
KO	Small	0.385	294.11	179.27	2.352	267.15	0.291	512.95	156.525
KO	Small	0.368	291.49	177.50	1.905	252.83	0.268	492.37	143.885
KO	Small	0.424	272.51	169.00	2.289	242.53	0.280	503.36	144.267
WT	Total	0.201	375.44	208.82	2.997	314.92	0.429	689.31	17.842
WT	Total	0.212	384.72	210.96	2.911	303.79	0.414	662.90	16.378
WT	Total	0.203	385.27	213.94	3.085	291.50	0.431	644.45	15.940
WT	Small	2.084	296.47	192.94	19.16	200.63	0.907	434.30	130.290
WT	Small	2.504	284.58	184.92	18.72	176.93	0.977	414.36	102.734
WT	Small	2.825	293.87	187.81	17.05	209.14	0.979	368.28	146.828

Supplementary Table 13. Nucleoside concentration (ng/mL) in total RNA and mRNA of WT HEK 293T and NSUN3 KO cells measured by LC-QQQ-MS. Experiments were performed in triplicate. Nucleosides marked with † were quantified in 200 ng of RNA. All others were quantified in 1 ng of RNA. Raw values correspond to data of Fig. 3G in the main text.

Cell Line	RNA Type	hm5C†	C	m5C	U	A	f5C†	G	i6A†
KO	Total	0.206	167.64	0.204	72.60	105.49	0.225	287.75	17.82
KO	Total	0.224	151.96	0.194	64.22	101.31	0.201	262.33	16.25
KO	Total	0.209	178.89	0.240	75.74	120.96	0.209	313.67	18.78
KO	mRNA	0.000	120.70	4.97†	90.78	127.90	0.053	199.21	0.511
KO	mRNA	0.000	87.539	3.60†	68.83	92.82	0.043	145.31	0.439
KO	mRNA	0.000	73.248	3.62†	56.87	73.76	0.042	118.83	0.414
WT	Total	0.257	179.35	0.242	77.66	121.99	0.410	318.17	18.09
WT	Total	0.282	212.74	0.224	89.48	141.31	0.485	368.46	19.45
WT	Total	0.254	173.66	0.217	81.19	127.24	0.445	328.86	19.89
WT	mRNA	0.000	174.50	7.24†	123.0	175.64	0.163	293.91	0.442
WT	mRNA	0.000	81.657	3.12†	59.29	177.67	0.090	133.95	0.558
WT	mRNA	0.000	112.8	3.96†	80.80	108.10	0.103	182.11	0.478

Supplementary Table 14. Nucleoside concentration (ng/mL) in total and small RNA of WT HEK 293T and NSUN3 KO cells measured by LC-QQQ-MS. Experiments were performed in triplicate. Nucleosides marked with † were quantified in 200 ng of RNA. All others were quantified in 1 ng of RNA. Raw values correspond to data of Fig. 3G in the main text.

Cell Line	RNA Type	hm5C†	C	U	m5C	A	f5C†	G	i6A†
KO	Total	0.2565	165.64	73.46	2.043	112.62	0.238	289.86	15.79
KO	Total	0.171	158.82	68.91	1.501	105.98	0.227	274.22	16.19
KO	Total	0.2074	181.46	82.84	2.059	113.95	0.223	307.37	17.29
KO	Small	2.1472	179.03	89.46	12.58	134.53	1.117	309.97	0.724
KO	Small	2.1101	188.96	95.79	12.27	145.24	1.144	333.99	0.732
KO	Small	1.8802	180.19	93.53	13.21	137.64	1.041	317.96	0.714
WT	Total	0.237	178.83	79.35	2.094	119.82	0.415	309.23	17.06
WT	Total	0.2917	171.43	75.92	1.851	113.33	0.390	293.85	17.76
WT	Total	0.2811	176.12	76.87	2.095	115.54	0.407	299.21	15.55
WT	Small	2.9448	181.61	86.89	12.81	134.73	1.084	311.28	0.728
WT	Small	2.8748	172.69	81.62	12.45	126.27	1.030	293.21	0.668
WT	Small	2.6429	186.68	87.42	12.95	133.99	1.058	310.47	0.711

Supplementary Table 15. m^{1}A Nucleoside concentration (ng/mL) in total RNA and small of WT HEK 293T and ALKBH1 cells under stress measured by LC-QQQ-MS.N.T., no treatment; H.S., heat shock (42 ^{o}C for 1 hour); Gluc., glucose deprivation (5 mM glucose for 8 hours); Ars. high, oxidative stress (0.5 mM $NaAsO_2$ for 1 hour); Ars. low, oxidative stress (0.2 mM $NaAsO_2$ for 4 hour). Experiments were performed in triplicate. Nucleosides marked with † were quantified in 200 ng of RNA. All others were quantified in 1 ng of RNA. Raw values correspond to the data of Supplementary Figs. 24-25.

Cell Line	RNA Type	C	m5C	U	A	G	m1A	i6A	f5C†	treatment
WT	total	561.1	4.08	227.2	156.0	516.5	2.18	17.8	0.1882	none
WT	total	531.5	4.90	273.9	228.0	602.1	2.02	21.2	0.1686	none
WT	total	408.0	3.56	213.1	169.1	602.1	1.44	13.4	0.147	none
KO	total	387.5	2.88	187.3	290.3	602.2	1.58	15.8	0.0121	none
KO	total	417.8	3.72	209.1	314.1	653.4	1.91	18.2	0.0123	none
KO	total	345.1	3.07	169.1	244.8	653.4	1.39	13.3	0.0156	none
WT	small	252.6	20.30	141.6	98.1	369.4	6.60	91.9	0.3174	none
WT	small	299.2	24.11	163.9	100.2	323.6	7.97	136.4	0.397	none
WT	small	200.6	16.16	108.5	80.4	323.6	4.26	149.7	0.218	none
KO	small	234.9	16.71	127.5	159.4	329.3	10.09	102.9	0.0661	none
KO	small	294.3	23.02	164.9	164.4	380.5	12.41	133.1	0.0392	none
KO	small	235.1	17.74	129.4	130.0	380.5	9.42	150.8	0.0639	none
WT	total	368.7	3.90	182.1	181.7	448.0	1.83	17.7	0.1358	H.S.
WT	total	407.5	3.74	200.0	223.5	216.9	1.91	14.6	0.1169	H.S.
WT	total	370.0	3.66	182.7	170.4	216.9	1.41	12.3	0.122	H.S.
KO	total	366.5	3.62	173.8	273.3	575.5	1.70	18.2	0.0142	H.S.
KO	total	411.8	3.92	203.3	302.2	637.6	2.06	21.4	0.0114	H.S.
KO	total	353.5	3.49	171.3	250.4	637.6	1.43	13.5	0.0132	H.S.
WT	small	114.9	15.24	84.8	98.2	200.5	1.90	143.3	0.1618	H.S.
WT	small	290.5	24.23	159.4	124.5	152.3	4.67	113.2	0.3515	H.S.
WT	small	291.3	23.95	165.0	113.5	120.5	4.69	179.7	0.3684	H.S.
KO	small	304.0	24.35	163.6	186.2	423.1	13.52	92.9	0.082	H.S.
KO	small	328.3	26.75	177.6	164.0	397.7	14.37	154.4	0.0445	H.S.
KO	small	280.5	20.86	153.4	153.5	397.7	11.94	192.5	0.0532	H.S.
WT	total	428.2	3.73	216.4	187.4	329.2	1.65	15.3	0.1523	Ars. low
WT	total	458.8	4.56	230.9	190.4	297.4	2.05	17.0	0.1683	Ars. low
WT	total	329.3	3.10	164.4	160.4	300.8	1.53	13.4	0.118	Ars. low
KO	total	366.5	3.46	178.4	271.1	569.6	1.62	16.5	0.0146	Ars. low
KO	total	398.3	3.69	200.1	253.7	578.2	1.95	19.5	0.0099	Ars. low
KO	total	339.8	3.07	172.2	220.8	578.2	1.29	13.2	0.0119	Ars. low
WT	small	312.1	22.36	176.3	120.9	392.2	4.11	143.3	0.3882	Ars. low

WT	small	299.7	20.60	171.2	111.3	250.4	3.47	106.5	0.3732	Ars. low
WT	small	326.2	24.46	188.3	125.6	322.7	4.55	208.0	0.3851	Ars. low
KO	small	356.8	26.10	197.2	298.0	578.0	14.74	149.5	0.0805	Ars. low
KO	small	300.9	21.48	170.1	178.9	406.5	11.88	122.1	0.0943	Ars. low
KO	small	351.7	25.78	191.9	123.1	406.5	13.45	192.9	0.0932	Ars. low
WT	total	439.0	4.48	219.2	269.2	461.6	1.72	18.4	0.1525	Ars. high
WT	total	389.9	3.72	197.9	263.6	429.9	1.40	13.7	0.1071	Ars. high
WT	total	234.0	2.29	324.4	197.4	309.9	1.08	13.0	0.088	Ars. high
KO	total	402.1	3.88	198.5	296.1	621.6	1.83	17.5	0.0144	Ars. high
KO	total	393.2	3.75	196.2	262.1	572.4	1.87	19.2	0.012	Ars. high
KO	total	354.6	3.22	174.3	240.5	572.4	1.50	14.1	0.0127	Ars. high
WT	small	247.9	19.39	139.6	147.3	290.2	3.12	144.7	0.2993	Ars. high
WT	small	312.4	23.56	180.9	198.3	356.6	4.70	113.6	0.4271	Ars. high
WT	small	280.4	22.43	161.1	246.3	302.5	2.90	146.6	0.3255	Ars. high
KO	small	278.7	20.24	155.7	153.3	349.2	11.30	125.5	0.0853	Ars. high
KO	small	174.5	12.75	100.2	148.2	288.0	6.46	71.1	0.0364	Ars. high
KO	small	277.0	18.51	155.3	184.2	387.1	10.71	161.3	0.0786	Ars. high
WT	total	434.4	4.11	215.7	195.6	495.5	1.94	17.0	0.1578	Gluc.
WT	total	437.7	4.27	221.5	189.5	379.1	1.95	17.6	0.1441	Gluc.
WT	total	400.0	3.43	199.0	172.0	307.1	1.54	11.8	0.133	Gluc.
KO	total	350.1	3.31	172.6	266.5	560.3	1.54	16.5	0.0091	Gluc.
KO	total	387.2	3.27	192.8	266.6	576.5	1.80	18.6	0.0127	Gluc.
KO	total	363.0	3.30	178.1	252.2	576.5	1.59	12.1	0.013	Gluc.
WT	small	341.9	28.58	196.6	184.4	411.3	3.69	151.7	0.4632	Gluc.
WT	small	299.4	24.51	168.5	167.4	428.0	3.72	137.7	0.4227	Gluc.
WT	small	320.4	26.53	178.2	190.4	421.3	4.42	238.9	0.3255	Gluc.
KO	small	335.8	26.27	183.6	264.5	507.3	14.66	144.5	0.0742	Gluc.
KO	small	433.6	34.87	251.1	235.2	546.5	18.62	183.9	0.0887	Gluc.
KO	small	332.5	25.42	180.0	150.4	546.5	13.70	192.2	0.0835	Gluc.

Supplementary Table 16. Quantification of f^5C and hm^5C (ng/mL) content in native tRNAs isolated from WT or ALKBH1 KO cells. Nucleoside levels were measured in 100-600 ng RNA by LC-QQQ-MS. Values correspond to Fig. 5B-G in main text. Nucleosides marked with † were recorded from injections with 24x more material. Values correspond to Fig. 6B-G in main text.

tRNA	Cell Line	hm5C	C	U	m5C	A	f5C	G	i6A
Leu CAA	KO	0.41†	988.92	240.40	31.06	480.77	0.08†	1016.1	0.96
Leu CAA	KO	0.35†	959.98	236.79	32.67	464.79	0.08†	996.9	0.99
Leu CAA	KO	0.29†	919.46	236.06	24.68	459.08	0.09†	982.2	0.98
Leu CAA	WT	3.71†	1264.9	256.59	35.84	500.67	0.65†	1111.4	1.28
Leu CAA	WT	3.57†	1275.3	254.28	34.12	500.43	0.75†	1105.9	1.19
Leu CAA	WT	3.72†	1218.3	254.49	34.29	507.86	0.56†	1109.2	1.17
mt-Met	KO	0.09	113.90	83.59	5.12	56.82	0.09	161.74	0.37
mt-Met	KO	0.12	110.18	79.25	4.67	57.72	0.07	156.72	0.40
mt-Met	KO	0.08	123.02	61.91	4.68	49.95	0.07	129.45	0.29
mt-Met	WT	0.22	102.72	85.69	3.64	61.97	1.88	144.96	0.38
mt-Met	WT	0.31	103.40	84.85	3.50	64.33	1.81	145.42	0.37
mt-Met	WT	0.23	106.25	62.52	2.77	49.91	1.47	111.81	0.24
Val CAC	KO	0.03	100.54	140.43	3.96	201.47	0.11	355.97	0.04
Val CAC	KO	0.05	95.26	143.73	3.44	200.07	0.11	352.66	0.04
Val CAC	KO	0.04	99.81	148.75	3.16	212.73	0.10	362.09	0.06
Val CAC	WT	0.02	76.11	125.91	2.10	189.83	0.07	331.19	0.10
Val CAC	WT	0.03	85.30	132.90	2.91	207.83	0.10	338.22	0.09
Val CAC	WT	0.04	93.54	143.28	3.06	235.44	0.09	339.04	0.10
Glu CTC	KO	0.00	1140.2	708.18	7.57	335.36	0.36	1647.0	0.28
Glu CTC	KO	0.00	1210.8	1094.1	10.50	581.27	0.41	2795.5	0.20
Glu CTC	KO	0.00	141.15	422.75	0.98	234.55	0.05	967.12	0.22
Glu CTC	WT	0.00	520.59	300.48	3.12	225.86	0.23	738.73	0.25
Glu CTC	WT	0.00	585.25	489.55	4.95	243.33	0.29	1270.1	0.81
Glu CTC	WT	0.00	153.12	479.13	1.12	277.18	0.07	1242.3	0.27
Gly CCC	KO	0.00	250.85	258.30	2.56	127.59	0.26	527.64	0.27
Gly CCC	KO	0.00	360.71	425.39	1.80	230.43	0.28	895.29	0.20
Gly CCC	KO	0.00	59.86	204.64	0.21	111.63	0.05	433.38	0.32
Gly CCC	WT	0.00	136.95	193.03	0.42	82.51	0.26	336.67	0.48
Gly CCC	WT	0.00	140.99	308.25	0.68	136.46	0.21	562.69	0.45
Gly CCC	WT	0.00	48.80	202.66	0.15	117.45	0.07	479.18	0.45
Gln CTG	KO	0.00	435.16	397.24	2.82	268.50	0.16	725.22	0.32
Gln CTG	KO	0.00	498.91	648.17	2.28	563.49	0.21	1448.4	0.22

Gln CTG	KO	0.00	80.20	284.24	0.42	224.15	0.04	556.34	0.24
Gln CTG	WT	0.00	374.17	328.32	1.86	215.72	0.15	579.98	0.30
Gln CTG	WT	0.00	400.32	519.08	2.25	371.06	0.19	1002.6	0.26
Gln CTG	WT	0.00	90.73	343.58	0.49	272.67	0.03	668.84	0.24

Supplementary Table 17. Sequences of labeled probes for tRNA antisense pulldown and northern blotting. Bio = biotin.

RNA	Sequence
mt-tRNA-Met	5’ Bio-TGGTAGTACGGGAAGGGTATAACCAACATT
tRNA-Leu-CAA	5’ Bio-GAGTCTGGCGCCTTAGACCACTCGGCCATCCTGAC
tRNA-Glu-CTC	5’ Bio-TTCCCTGACCGGGAATCGAACCCGGGCCG
tRNA-Gly-CCC	5’ Bio-GGAGGCGAGAATTCTACCACTGAACCACCAATGC
tRNA-Gln-CTG	5’ Bio-AGGTCCCACCGAGATTTGAACTCGGATCGCTGG
tRNA-Val-CAC	5’ Bio-TGTTTCCGCCCGGTTTCGAACCGGGGACCTTTCGCGT
tRNA-iMet-CAT	5’ Bio-TAGCAGAGGATGGTTTCGATCCATCA
tRNA-Leu-CAG	5’ Bio-GCGCCTTAGACCGCTCGGCCATCCTGAC
tRNA-Trp-CCA	5’ Bio-TGACCCCGACGTGATTTGAACACGCAACCT
tRNA-Lys-CTT	5’ Bio-CCAACGTGGGGCTCGAACCCACGACCCT
tRNA-Gly-GCA	5’ Bio-AGTCAAATGCTCTACCACTGAGCTATACCCCC
tRNA-eMet-CAT	5’ Bio-TGCCCCGTGTGAGGATCGAACTCACGACCT
tRNA-Arg-CCT	5’ Bio-CACCCCAGGTGGGACTCGAACCCACAAT
5S RNA	5’ FAM-AAAGCCTACAGCACCCGGTAT

Supplementary Table 18. Generation of f^5C and hm^5C (ng/mL) in tRNA ASL substrates after oxidation with ALKBH1 *in vitro*. Nucleoside levels were measured by LC-QQQ-MS. Values correspond to Fig. 7C in main text.

ASL	Enz.	hm5C	C	m5C	U	A	f5C	G
iMet CAT	–	0	66.4	19.23	49.50	100.01	0	103.98
iMet CAT	–	0	66.11	19.94	48.43	96.46	0	103.04
iMet CAT	–	0	65.92	18.89	48.55	95.16	0	101.65
iMet CAT	+	1.20	38.83	7.87	28.72	58.47	1.085	58.56
iMet CAT	+	1.84	38.45	7.78	28.71	57.25	1.208	58.62
iMet CAT	+	1.12	38.42	7.93	28.98	55.93	1.100	57.52
Val CAC	–	0	114.29	25.4	73.52	145.61	0	126.28
Val CAC	–	0	113.80	25.71	73.48	141.86	0	124.21
Val CAC	–	0	114.80	27.44	73.23	139.50	0	123.94
Val CAC	+	3.63	55.24	6.30	35.49	72.39	4.141	60.23
Val CAC	+	2.56	55.70	6.17	35.84	71.61	4.203	59.44
Val CAC	+	2.52	56.50	6.16	36.27	70.79	4.141	59.32
Leu CAA	–	0	7.26	6.88	22.74	21.92	0	40.14
Leu CAA	–	0	7.62	5.93	23.38	21.75	0	39.45
Leu CAA	–	0	7.60	6.28	24.08	21.67	0	39.51
Leu CAA	+	5.20	35.29	9.12	65.91	78.74	3.801	111.50
Leu CAA	+	4.84	35.68	9.33	67.61	77.51	3.095	108.67
Leu CAA	+	5.12	35.76	9.33	66.79	76.00	3.419	107.23
Gln CTG	–	0	2.67	2.08	9.88	9.75	0.028	14.28
Gln CTG	–	0	4.23	1.91	9.91	9.33	0.038	13.70
Gln CTG	–	0	4.53	2.47	10.65	10.43	0.0414	15.24
Gln CTG	+	0.16	1.35	5.65	20.01	30.99	0.600	30.12
Gln CTG	+	0.17	0.98	5.05	17.79	26.67	0.555	25.97
Gln CTG	+	0.15	0.92	5.02	18.45	25.73	0.421	24.58
Glu CTC	–	0	5.24	2.37	9.76	2.85	0.042	19.88
Glu CTC	–	0	2.35	1.21	5.37	1.26	0.018	9.54
Glu CTC	–	0	4.25	1.44	8.28	1.54	0.022	10.25
Glu CTC	+	0.91	3.36	3.84	12.51	7.38	1.508	27.66
Glu CTC	+	0.91	6.36	3.71	14.00	6.13	1.510	32.67
Glu CTC	+	1.13	7.18	4.25	18.48	7.25	2.005	43.25
Gly CCC	–	0	181.02	51.72	182.76	106.48	0.169	524.57
Gly CCC	–	0	228.17	64.90	214.44	144.48	0.190	608.86
Gly CCC	–	0	203.57	59.35	201.57	122.35	0.174	500.22
Gly CCC	+	31.9	290.86	48.17	264.00	317.57	48.81	729.06
Gly CCC	+	35.6	292.63	60.23	278.56	330.88	46.24	759.72

Gly CCC	+	28.7	250.53	50.5	250.53	300.56	36.57	600.25
Leu CAG	–	0	1.58	4.44	19.53	9.77	0.165	51.77
Leu CAG	–	0	1.52	2.25	9.82	3.96	0.063	24.08
Leu CAG	–	0	0.38	1.14	7.36	2.45	0.044	18.76
Leu CAG	+	4.65	13.38	8.36	44.08	45.87	6.605	121.83
Leu CAG	+	1.21	4.35	2.37	14.13	11.49	1.545	35.51
Leu CAG	+	2.65	3.58	4.92	34.42	27.57	3.485	70.27
mt-Met	–	0	51.21	32.64	56.85	160.08	0.314	143.43
mt-Met	–	0	38.61	28.63	44.34	141.29	0.215	118.09
mt-Met	–	0	40.85	25.55	42.47	130.74	0.201	111.47
mt-Met	+	3.62	58.60	20.73	61.00	195.09	10.853	158.96
mt-Met	+	0.59	3.69	3.27	10.42	27.20	1.234	23.64
mt-Met	+	3.24	16.27	18.27	50.27	150.25	8.352	100.54

Supplementary Table 19. ca^5C nucleoside concentration (ng/mL) in various tRNA ASLs after *in vitro* oxidation with ALKBH1 as measured by LC-QQQ-MS. Experiments were performed in triplicate. Raw values correspond to the data of Supplementary Fig. 22

ASL	ALKBH1	hm5C	C	ca5C	m5C	U	A	f5C	G
mt-Met	No	0	90.4	0	22.2	47.7	111.9	0	171.3
mt-Met	No	0	112.2	0	25.1	55.5	136.5	0	198.0
mt-Met	No	0	120.1	0	28.4	60.1	145.8	0	209.5
Leu CAA	No	0	22.8	0	7.84	25.5	35.5	0	98.0
Leu CAA	No	0	18.2	0	8.20	21.5	33.2	0	91.6
Leu CAA	No	0	16.3	0	6.11	15.4	25.2	0	81.0
Val CAC	No	0	31.6	0	7.82	17.3	41.8	0	87.9
Val CAC	No	0	39.4	0	9.44	22.3	50.6	0	97.7
Val CAC	No	0	32.8	0	7.60	17.0	39.5	0	86.8
mt-Met	Yes	4.95	164.8	0	21.6	81.4	184.0	11.0	251.7
mt-Met	Yes	3.97	128.1	0	13.9	63.8	146.4	11.0	208.2
mt-Met	Yes	15.9	457.3	0	59.3	234.7	514.0	29.6	638.8
Leu CAA	Yes	1.59	12.2	0	2.27	16.7	23.4	0.84	77.1
Leu CAA	Yes	1.52	12.2	0	2.16	15.1	23.8	0.85	74.2
Leu CAA	Yes	1.93	17.9	0	3.00	22.9	30.2	0.89	87.5
Val CAC	Yes	7.43	132.9	0	10.0	66.9	149.0	9.58	187.8
Val CAC	Yes	0.62	12.2	0	1.18	6.47	15.9	0.99	56.9
Val CAC	Yes	2.08	39.3	0	3.05	21.5	45.4	2.75	90.8

Supplementary Table 20. Generation of f^{5}C and hm^{5}C (ng/mL) and decrease of m^{5}C in substrates **3-5** after oxidation with ALKBH1 *in vitro*. Nucleoside levels were measured by LC-QQQ-MS. The ASL of mt-tRNA-Met (mt-Met) is included as a positive control. Values correspond to Fig. 7E in main text.

Substrate	Enz	hm5C	C	U	m5C	A	f5C	G
3	No	0.00	107.90	92.86	43.15	278.08	0.00	127.34
3	No	0.00	106.93	92.07	42.01	269.61	0.00	122.12
3	No	0.00	106.50	90.62	42.20	265.65	0.00	120.04
4	No	0.00	224.85	313.30	74.63	388.05	0.00	244.49
4	No	0.00	223.55	307.20	74.49	382.95	0.00	242.03
4	No	0.00	224.16	310.75	75.10	385.15	0.00	243.56
5	No	0.00	2706.02	4585.14	645.67	2848.96	0.00	5961.24
5	No	0.00	2523.48	4234.43	606.83	2697.73	0.00	5623.73
5	No	0.00	2307.67	4890.04	519.41	2627.61	0.00	5364.60
mt-Met	No	0.00	71.18	62.74	21.89	122.07	0.00	114.00
mt-Met	No	0.00	71.35	62.70	21.51	121.48	0.00	113.48
mt-Met	No	0.00	72.22	63.27	22.20	121.19	0.00	112.04
3	Yes	3.57	89.38	77.18	27.66	236.33	0.93	102.71
3	Yes	3.09	88.75	77.28	27.54	236.02	0.85	102.20
3	Yes	3.15	89.03	76.95	27.90	235.53	0.84	102.33
4	Yes	0.19	171.30	224.79	51.24	279.13	0.05	175.46
4	Yes	0.12	170.42	218.17	52.18	278.46	0.07	175.30
4	Yes	0.17	172.25	225.71	54.39	278.48	0.05	172.99
5	Yes	3.34	2793.24	4859.18	654.11	2731.75	1.76	5403.80
5	Yes	3.73	2873.43	4897.33	643.54	2738.18	1.69	5431.68
5	Yes	3.28	2864.30	4585.24	645.66	2658.31	1.73	5404.35
mt-Met	Yes	4.28	49.48	42.14	9.69	88.49	1.63	72.50
mt-Met	Yes	4.85	49.59	42.83	9.77	88.87	1.58	72.78
mt-Met	Yes	4.84	49.89	43.92	9.77	89.09	1.79	73.45

Supplementary Table 21. Generation of f^{5}C and hm^{5}C (ng/mL) and decrease of m^{5}C in substrates **6-8** after oxidation with ALKBH1 *in vitro*. Nucleoside levels were measured by LC-QQQ-MS. The ASL of mt-tRNA-Met (mt-Met) is included as a positive control. Values correspond to Fig. 7E in main text.

Substrate	Enz.	hm5C	C	U	m5C	A	f5C	G
6	no	0.00	0.00	1.67	0.58	1.114	0.00	7.75
6	no	0.00	2.02	3.16	0.89	2.094	0.00	12.61
6	no	0.00	1.95	2.38	0.68	1.584	0.00	10.14
7	no	0.00	1.98	3.49	1.00	2.229	0.00	9.94
7	no	0.00	12.17	3.73	1.10	2.507	0.00	124.61
7	no	0.00	0.00	4.02	1.25	2.674	0.00	9.35
8	no	0.00	2.05	3.92	1.23	2.623	0.00	11.47
8	no	0.00	2.14	3.06	0.95	1.995	0.00	15.47
8	no	0.00	0.00	1.59	0.45	1.100	0.00	7.74
mt-Met	no	0.00	20.81	74.84	22.12	50.20	0.29	95.05
mt-Met	no	0.00	44.69	128.71	43.39	86.15	0.37	209.56
mt-Met	no	0.00	0.00	10.55	3.55	7.039	0.07	26.30
6	yes	0.00	2.28	3.40	0.97	2.453	0.00	14.65
6	yes	0.00	12.34	4.40	1.27	2.944	0.00	118.96
6	yes	0.00	0.00	8.30	2.58	5.522	0.00	10.73
7	yes	0.00	2.59	3.04	1.03	1.990	0.00	14.81
7	yes	0.00	2.47	3.21	0.93	2.175	0.00	12.25
7	yes	0.00	0.00	4.16	1.21	2.774	0.00	11.21
8	yes	0.00	2.32	3.40	0.86	2.305	0.13	13.81
8	yes	0.00	3.39	3.05	0.88	2.048	0.14	20.43
8	yes	0.00	0.00	3.18	0.82	2.106	0.11	9.79
mt-Met	yes	1.26	18.61	37.69	7.12	25.063	4.35	86.36
mt-Met	yes	1.57	23.08	41.72	7.54	28.356	4.13	104.25
mt-Met	yes	1.12	21.15	36.76	7.43	25.501	4.52	102.92

Supplementary Table 22. Fluc/Rluc normalized dual-luciferase assay signals detected in WT, ALKBH1 KO, or NSUN2 KO cell lines using different reporter constructs for Leu translation. Biological triplicates were analyzed. Values correspond to data in Supplementary Fig. 37.

[Glucose]	Cell Line	Construct	Fluc/Rluc
25 mM	WT	TTG	0.522593
25 mM	WT	TTG	0.500958
25 mM	WT	TTG	0.490108
25 mM	WT	TTA	0.496131
25 mM	WT	TTA	0.454656
25 mM	WT	TTA	0.533748
25 mM	WT	Random	0.441912
25 mM	WT	Random	0.559697
25 mM	WT	Random	0.567572
1 mM	WT	TTG	0.498829
1 mM	WT	TTG	0.450062
1 mM	WT	TTG	0.436086
1 mM	WT	TTA	0.450586
1 mM	WT	TTA	0.497583
1 mM	WT	TTA	0.516315
1 mM	WT	Random	0.530949
1 mM	WT	Random	0.513912
1 mM	WT	Random	0.468501
25 mM	ALKBH1 KO	TTG	0.421198
25 mM	ALKBH1 KO	TTG	0.537379
25 mM	ALKBH1 KO	TTG	0.511532
25 mM	ALKBH1 KO	TTA	0.452581
25 mM	ALKBH1 KO	TTA	0.495098
25 mM	ALKBH1 KO	TTA	0.50427
25 mM	ALKBH1 KO	Random	0.563185
25 mM	ALKBH1 KO	Random	0.556884
25 mM	ALKBH1 KO	Random	0.576555
1 mM	ALKBH1 KO	TTG	0.460817
1 mM	ALKBH1 KO	TTG	0.460831
1 mM	ALKBH1 KO	TTG	0.499743
1 mM	ALKBH1 KO	TTA	0.418712

1 mM	ALKBH1 KO	TTA	0.366305
1 mM	ALKBH1 KO	TTA	0.424741
1 mM	ALKBH1 KO	Random	0.56367
1 mM	ALKBH1 KO	Random	0.514439
1 mM	ALKBH1 KO	Random	0.519565

Supplementary Note: Synthetic Protocols and Compound Characterization

General synthetic methods S66

Synthesis of 5-ethynylcytidine (5-EC) (**3**) S67

Synthesis of 5-ethnylcytidine phosphoramidite (**11**) S68

Compound Characterization (NMR and ESI-MS) S76

General synthetic methods

Reagents were purchased in the highest available quality from commercial suppliers (Sigma-Aldrich, Chem Genes, Glen Research, Carbo Synth) and used without further purification. All reactions were carried out under argon or nitrogen atmosphere, unless otherwise noted. Analytical thin-layer chromatography (TLC) was performed on Silicycle SiliaPlate Glass Backed TLC Extra Hard Layer 60Å plates. Silica gel 230-400 mesh Grade 60 for column chromatography was purchased from Fischer Scientific. ^{1}H, ^{13}C, and ^{31}P spectra were recorded on a Bruker Ultrashield^{TM} 300, 400, or 500 Plus spectrometer. Chemical shifts (δ) are referenced to the residual solvent signal (DMSO-d_6: 2.50 ppm for ^{1}H and 39.52 ppm for ^{13}C spectra; $CDCl_3$: 7.26 ppm for 1 H and 77.16 ppm for ^{13}C spectra). The following abbreviations were used to denote multiplicities: s = singlet, d = doublet, t = triplet, q = quadruplet, m = multiplet, b = broad. Mass spectrometric analysis (HRMS) of nucleoside derivatives was performed on an ESI-TOF device. MS analysis of oligonucleotides was performed on an ESI-TOF device in negative mode.

Synthesis of 5-ethynylcytidine (5-EC) (3)

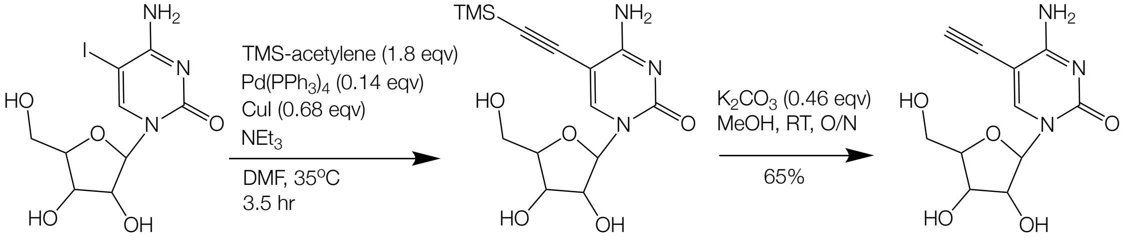

5-iodo cytidine (**1**)
1 eqv, 2.7 mmol

(**2**)

5-ethynyl cytidine (**3**)

TMS-protected 5-ethynylcytidine (**2**). 5-iodocytidine (**1**, 1 g, 2.7 mmol) was added to a 250 mL round-bottomed flask, and the flask was evacuated and backfilled with inert gas (3x). Anhydrous DMF (44 mL) and anhydrous triethylamine (35 mL) were added via syringe. TMS-acetylene (0.478 g, 1.8 mmol), tetrakis(triphenylphosphine)palladium(0) [$Pd(PPh_3)$, 0.483 g, 0.38 mmol], and copper iodide (CuI, 0.348 g, 1.8 mmol), were added under positive pressure of inert gas, and the mixture was stirred for 4.5 hr at 35 °C. Volatiles were evaporated, and the residue was re-dissolved in methanol, filtered through celite, and concentrated *in vacuo*. The crude was purified by column chromatography (silica, 10-25% methanol in DCM) to yield (**2**) as a yellow solid (522 mg, 55%). ^{1}H NMR (500 MHz, DMSO-d6): 0.22 (s, 9H, 3xCH3); 3.58 (ddd, 1H, H5'); 3.69 (ddd, 1H, H5''); 3.83 (dt, 1H, H4'); 3.95 (m, 2H, H2' + H3'); 5.00 (d, 1H, 2'-OH); 5.18 (t, 1H, 5'-OH); 5.37 (d, 1H, 3'-OH); 5.75 (d, 1H, H10'); 6.65 (s, br, 1H, NH); 7.80 (s, br, 1H, NH); 8.33 (s, 1H, H8). HRMS ESI-TOF $[M+Na]^+$ calc: 290.07174, found: 290.07139.

5-ethynylcytidine (**3**). Intermediate **2** (522 mg, 1.54 mmol) was dissolved in anhydrous methanol (15 mL), and potassium carbonate (K_2CO_3, 64 mg, 0.46 mmol) was added. The mixture was stirred at room temperature for 16 hours, over the course of which a

fine precipitate was formed. The reaction was vacuum-filtered, and the filter cake was washed with cold methanol. The filtrate was concentrated *in vacuo* and purified by column chromatography (silica, 15-25% methanol in DCM) to yield (**3**) as a light-yellow solid (268 mg, 65%). ^{1}H NMR (500 MHz, DMSO-d6): 3.59 (ddd, 1H, H5'); 3.71 (ddd, 1H, H5''); 3.85 (dt, 1H, H4'); 3.94 (m, 2H, H2' + H3'); 4.35 (s, 1H, CCH); 5.00 (d, 1H, 2'-OH); 5.21 (t, 1H, 5'-OH); 5.39 (d, 1H, 3'-OH); 5.74 (d, 1H, H10'); 6.85 (s, br, 1H, NH); 7.73 (s, br, 1H, NH); 8.38 (s, 1H, H8). ^{13}C NMR (125 MHz, DMSO-d6): 60.36 (C5'), 69.25 (C3'), 70.84 (*C*CH), 76.32 (C2'), 84.45 (C4'), 86.32 (C*C*H), 89.29 (C1'), 89.91 (C5), 146.29 (C6), 154.1 (C4), 164.62 (C2). HRMS ESI-TOF $[M+Na]^+$ calc: 290.07165, found: 290.07474.

Synthesis of 5-ethynylcytidine (5-EC) phosphoramidite (11)

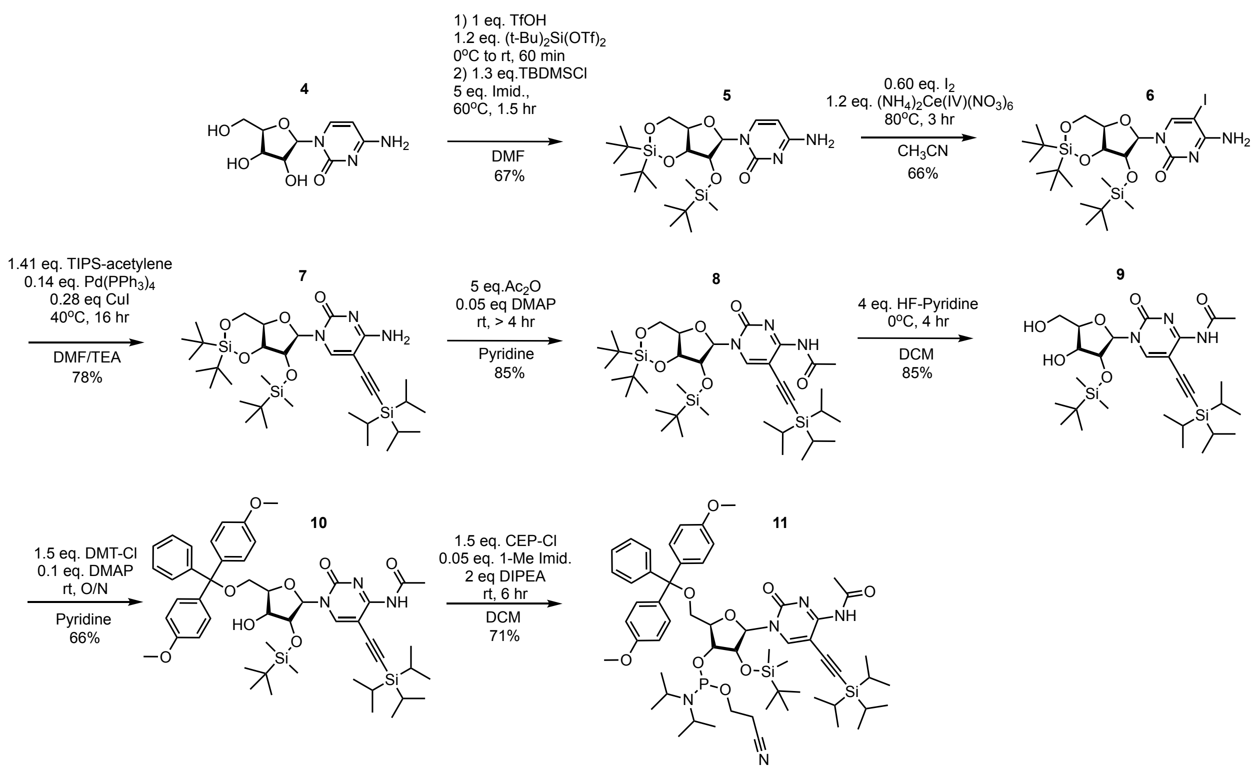

2′-*O*-(*tert*-butyldimethylsilyl)-3′,5′-*O*-(di-*tert*-butylsilylene)- cytidine (**5**). Cytidine (**4,** 2 g, 8.2 mmol) was dried under high vacuum then dissolved in anhydrous DMF (20 mL) then cooled to 0°C on ice. Then, TfOH (726 µL, 1.23 g, 8.2 mmol) was added via a syringe followed $(t\text{-}Bu)_2Si(OTf)_2$ (3.21 mL, 4.35 g, 9.86 mmol). Reaction progress was monitored by TLC. After 1 hour, the reaction was removed from the ice bath and warmed to rt. Imidazole (2.79 g, 41 mmol) was added and the reaction was heated to 60°C. TBDMS-Cl (1.62 g, 10.6 mmol) was added, and the reaction stirred for an additional 1.5 hours. The reaction was diluted with 200 mL EtOAc and extracted with 200 mL $NaHCO_3$ (x2) followed by washes with 200 mL H_2O (x2). The organic layer was dried over Na_2SO_4 and filtered. The solution was then evaporated and the crude residue purified by SiO_2 column chromatography pre-equilibrated with 0.1% triethylamine in DCM using a 1-4% MeOH in DCM gradient. This afforded compound **5** (2.75 g, 5.53 mmol, 67%) as a white foam once dried under high vacuum. R_f = 0.44 (7% MeOH in DCM). HR-MS (ESI-TOF) calculated [MH^+] = 498.2814, observed [MH^+] = 498.3130. ^{1}H-NMR (400 MHz, $CDCl_3$) δ = 0.14 (s, 3H, H_3C-Si), 0.21 (s, 3H, H_3C-Si), 0.93 (s, 9H, H_3C-Si), 1.02 (s, 9H, H_3C-Si), 1.04 (s, 9H, H_3C-Si), 3.85 (dd, 1H, H-C(3’)), 3.98 (t, 1H, H(a)-C(5’)), 4.20 (m, 1H, H-C(4’)), 4.29 (d, 1H, H-C(2’)), 4.50 (dd, 1H, H(b)-C(5’)), 5.67 (s, 1H, H-C(1’)), 5.84 (d, 1H, H-C(5)), 7.34 (s, 1H, H-C(6)). ^{13}C-NMR (126 MHz, $CDCl_3$) δ = -4.86, -4.30 (2x $SiCH_3$), 18.25, 20.34, 22.78 (3x $SiC(CH_3)_3$), 25.94, 27.00, 27.52 (3x $SiC(CH_3)_3$), 67.83 (C(5’)), 74.41 (C(4’)), 75.29 (C(2’)), 75.90 (C(3’)), 94.50 (C(1’)), 94.87 (C(5)), 140.23 (C(6)), 155.34 (C(2)), 165.74 (C(4)) ppm.

2′-*O*-(*tert*-butyldimethylsilyl)-3′,5′-*O*-(di-*tert*-butylsilylene)-5-iodocytidine (**6**). Compound **5** (2.73 g, 5.49 mmol) was dissolved in 30 mL anhydrous CAN. I_2 (0.835 g, 3.29 mmol) and ammonium (IV) cerium nitrate (3.61 g, 6.57 mmol) were added, and the reaction was refluxed at 80°C for 3 hr. The reaction was then cooled and diluted with 300 mL EtOAc and extracted twice with 200 mL $NaHCO_3$ and washed five times with $Na_2S_2O_3$. The organic layer was dried over Na_2SO_4, filtered, evaporated, and dissolved in a small amount of $CHCl_3$ then purified by silica gel column chromatography (0-5% acetone in $CHCl_3$) pre-equilibrated with toluene to afford compound **6** (2.27 g, 3.64 mmol, 66%) as a light-yellow foam. R_f = 0.656 (10% MeOH in $CHCl_3$). HR-MS (ESI-TOF) calculated [MH^+] = 624.1780, observed [MH^+] = 624.1226. ^{1}H-NMR (300 MHz, $CDCl_3$) δ = 0.15 (s, 3H, H_3C-Si), 0.22 (s, 3H, H_3C-Si), 0.94 (s, 9H, H_3C-Si), 1.02 (s, 9H, H_3C-Si), 1.05 (s, 9H, H_3C-Si), 3.84 (dd, 1H, H-C(3’)), 3.98 (t, 1H, H(a)-C(5’)), 4.21 (m, 1H, H-C(4’)), 4.30 (d, 1H, H-C(2’)), 4.52 (dd, 1H, H(b)-C(5’)), 5.65 (s, 1H, H-C(1’)), 7.66 (s, 1H, H-C(6)). ^{13}C-NMR (126 MHz, $CDCl_3$) δ = -4.82, -4.31 (2x $SiCH_3$), 18.21, 20.35, 22.85 (3x $SiC(CH_3)_3$), 25.92, 26.99, 27.50 (3x $SiC(CH_3)_3$), 55.95 (C(5)), 67.84 (C(5’)), 74.57 (C(4’)), 75.16 (C(2’)), 75.72 (C(3’)), 94.43 (C(1’)), 146.69 (C(6)), 153.94 (C(2)), 163.47 (C(4)) ppm.

2′-*O*-(*tert*-butyldimethylsilyl)-3′,5′-*O*-(di-*tert*-butylsilylene)-5-((triisopropylsilyl)ethynyl)cytidine (**7**). Compound **6** (2.25 g, 3.6 mmol) was dissolved in 130 mL anhydrous DMF and transferred to a dry 3-neck round bottom flask. The solution was then degassed via three cycles of freeze-pump-thaw under high vacuum. Then, in order, CuI (0.194 g, 1.02 mmol), TIPS-acetylene (0.931 g, 5.11 mmol, 1.29 mL), $Pd(PPh_3)_4$ (0.590 g, 0.511 mmol), and 20 mL triethylamine were added. The reaction was

allowed to proceed overnight (~16 hours) at 40°C under a N_2 atmosphere in the dark. The reaction was then diluted with 200 mL EtOAc and washed with 200 mL H_2O and again with 200 mL 0.5 M EDTA and again with 200 mL brine. The organic layer was dried over Na_2SO_4, filtered, and evaporated. The product was dissolved in a small amount of DCM and purified via silica gel column chromatography (1-20% EtOAc in hexane) pre-equilibrated with toluene to afford compound **7** (1.9 g, 2.80 mmol, 78%) as a whitish foam after drying under high vacuum. R_f = 0.53 (40% EtOAc in hexane). HR-MS (ESI-TOF) calculated [MH$^+$] = 678.4148, observed [MH$^+$] = 678.4379. ^{1}H-NMR (400 MHz, $CDCl_3$) δ = 0.15 (s, 3H, H_3C-Si), 0.22 (s, 3H, H_3C-Si), 0.94 (s, 9H, H_3C-Si), 1.02 (s, 9H, H_3C-Si), 1.04 (s, 9H, H_3C-Si), 1.10 (d, 18H, H_3C-Si), 3.87 (dd, 1H, H-C(3')), 3.99 (t, 1H, H(a)-C(5')), 4.23 (m, 1H, H-C(4')), 4.28 (d, 1H, H-C(2')), 4.55 (dd, 1H, H(b)-C(5')), 5.71 (s, 1H, H-C(1')), 7.57 (s, 1H, H-C(6)). ^{13}C-NMR (126 MHz, $CDCl_3$) δ = -4.86, -4.35 (2x $SiCH_3$), 11.14 (3x $SiC(CH_3)_2$), 18.22, 18.65 (3x $SiC(CH_3)_2$), 20.33, 22.84, 25.92, 26.96, 27.49 (3x $SiC(CH_3)_3$), 67.79 (C(5')), 74.55 (C(4')), 75.32 (C(2')), 75.62 (C(3')), 91.29 (C(1')), 94.23 (C(8)), 97.41 (C(5)), 98.02 (C(7)), 143.57 (C(6)), 153.57 (C(2)), 164.36 (C(4)) ppm.

N^4-Acetyl-5-((triisopropylsylil)ethynyl)-3′,5′-di-*O*-*tert*-butylsilyl-2′-*O*-tert-butyldimethylsilylcytidine (**8**)**.** Compound **7** (1.9 g, 2.80 mmol) and DMAP (17.1 mg, 0.140 mmol) were dissolved in 28.6 mL anhydrous pyridine. Ac_2O (1.43 g, 14.0 mmol, 1.33 mL) was added, and the reaction proceeded overnight at rt. The solution was diluted with 75 mL DCM and washed with 100 mL 50% $NaHCO_3$ then twice with 100 mL of 5% citric acid, once with 100 mL H_2O, and once with 100 mL brine. The organic layer was dried over Na_2SO_4, filtered, evaporated, dissolved in a few mL of hexane/DCM, and purified by silica

gel column chromatography (0-10% EtOAc in hexane) pre-equilibrated with toluene to yield **8** (1.71 g, 2.38 mmol, 85%) as a light orange foam once dried under high vacuum. R_f = 0.69 (20% EtOAc in hexane). HR-MS (ESI-TOF) calculated [MH^+] = 720.4254, observed [MH^+] = 720.4405. ^{1}H-NMR (400 MHz, $CDCl_3$) δ = 0.17 (s, 3H, H_3C-Si), 0.26 (s, 3H, H_3C-Si), 0.96 (s, 9H, H_3C-Si), 1.03 (s, 9H, H_3C-Si), 1.07 (s, 9H, H_3C-Si), 1.12 (d, 18H, H_3C-Si), 2.35 (s, 3H, (H_3C)CO), 3.82 (dd, 1H, H-C(3')), 3.99 (t, 1H, H(a)-C(5')), 4.33 (m, 1H, H-C(4')), 4.36 (d, 1H, H-C(2')), 4.59 (dd, 1H, H(b)-C(5')), 5.69 and 5.75 (s, 1H, H-C(1')), 7.78 and 7.99 (s, 1H, H-C(6)). ^{13}C-NMR (126 MHz, $CDCl_3$) δ = -4.93, -4.18 (2x $SiCH_3$), 11.14 (3x $SiC(CH_3)_2$), 18.52, 18.62 (3x $SiC(CH_3)_2$), 20.36, 22.80, 25.89, 26.95, 27.49 (3x $SiC(CH_3)_3$), 26.11 ($COCH_3$), 67.64 (C(5')), 75.03 (C(4')), 75.21 (C(2')), 75.61 (C(3')), 94.75 (C(1')), 95.84 (C(8)), 98.59 (C(5)), 101.82 (C(7)), 147.26 (C(6)), 152.83 (C(2)), 166.93 (C(4)), 170.75 ($COCH_3$) ppm.

N^4-Acetyl-5-((triisopropylsylil)ethynyl)-2′-*O*-*tert*-butyldimethylsilylcytidine (**9**). Compound **8** (1.71 g, 2.38 mmol) was dissolved in 50 mL anhydrous DCM and cooled to 0°C. HF-pyridine (0.19 g, 9.51 mmol, 247 µL) was carefully mixed with 741 µL anhydrous pyridine in a microfuge tube and added to the reaction dropwise. Reaction progress was monitored by TLC (30% EtOAc in hexane) and showed a complete conversion of the starting material at 4 hours. The reaction was then diluted with 200 mL $CHCl_3$ and washed with 100 mL Na_2CO_3. The organic layer was dried over Na_2SO_4 and evaporated then dissolved in hexane/DCM and purified by silica gel column chromatography (10-15% EtOAc in hexane) which gave **9** (1.17 g, 2.02 mmol, 85%). R_f = 0.41 (30% EtOAc in hexane). HR-MS (ESI-TOF) calculated [MH^+] = 580.3233, observed [MH^+] = 580.3377. ^{1}H-NMR (500

MHz, $CDCl_3$) δ = 0.16 (s, 3H, H_3C-Si), 0.23 (s, 3H, H_3C-Si), 0.92 (s, 9H, $(H_3C)_3C$-Si), 1.05 (d, 18H, $(H_3C)_2C$-Si), 2.34 (s, 3H, $(H_3C)CO$), 3.90 (dd, 1H, H-C(3')), 4.10 (t, 1H, H(b)-C(5')), 4.23 (m, 1H, H-C(4')), 4.44 (d, 1H, H-C(2')), 4.53 (dd, 1H, H(a)-C(5')), 5.59 and 5.76 (s, 1H, H-C(1')), 8.44 and 8.90 (s, 1H, H-C(6)). ^{13}C-NMR (126 MHz, $CDCl_3$) δ = -5.33, -4.47 (2x $SiCH_3$), 11.06 (3x $SiC(CH_3)_2$), 18.08 (3x $SiC(CH_3)_2$), 18.63 ($SiC(CH_3)_3$), 25.82 ($COCH_3$), 26.09 ($SiC(CH_3)_3$), 60.29)c(5')), 68.39 (C(4')), 74.84 (C(2')), 85.02 (C(3')), 93.22 (C(1')), 93.99 (C(8)), 98.11 (C(5)), 101.07 (C(7)), 147.77 (C(6)), 150.30 (C(4)), 153.37 (C(2)), 170.66 ($COCH_3$) ppm.

N^4-Acetyl-5-((triisopropylsylil)ethynyl)-5'-*O*-(4,4'-dimethoxytrityl)-2'-*O*-*tert*-butyldimethylsilylcytidine (**10**). Compound **9** (1.17 g, 2.02 mmol) was dissolved in 30 mL anhydrous pyridine. DMT-Cl (1.03 g, 3.03 mmol) was added in portions over approximately 1 hour. DMAP (0.109 mmol, 13.3 mg) was added with the first portion of DMT-Cl. An additional 0.05 eq. DMAP (0.111 mmol, 13.5 mg) was added after 3 hours, and the reaction proceeded overnight. The solution was then diluted with 200 mL EtOAc and washed three times with 100 mL 5% citric acid, once with 200 mL $NaHCO_3$, once with 200 mL H_2O, and once with 200 mL brine. The organic layer was then dried over Na_2SO_4. The crude product was dissolved in $CHCl_3$ and purified by silica column chromatography (5-15% EtOAc in hexane) pre-equilibrated with toluene to afford **10** as a white foam (1.17 g, 1.33 mmol, 66%). R_f = 0.63 (30% EtOAc in hexane). HR-MS (ESI-TOF) calculated [MH^+] = 882.4539, observed [MH^+] = 882.4735. ^{1}H-NMR (300 MHz, DMSO-d6) δ = 0.08 (s, 3H, H_3C-Si), 0.10 (s, 3H, H_3C-Si), 0.88 (s, 9H, $(H_3C)_3C$-Si), 0.93 (d, 18H, $(H_3C)_2C$-Si), 2.37 (s, 3H, $(H_3C)CO$), 3.21 (dd, 1H, H(a)-C(5')), 3.36 (t, 1H, H(b)-

C(5')), 3.74 (s, 6H, OCH_3), 3.98 (dd, 1H, H-C(3')), 4.09 (m, 1H, H-C(4')), 4.29 (d, 1H, H-C(2')), 5.09 (d, 1H, HO-C(3')), 5.77 (s, 1H, H-C(1')), 6.90 (d, 4H, H-C(ar)), 7.22-7.42 (m, 9H, H-C(ar)), 8.25 (s, 1H, H-C(6)), 9.11 (s, 1H, H-N(4)). ^{13}C-NMR (126 MHz, DMSO-d6) δ = -4.51, -4.31 (2x $SiCH_3$), 11.01 (3x $SiC(CH_3)_2$), 18.71 (3x $SiC(CH_3)_2$), 18.75 ($SiC(CH_3)_3$), 25.41 ($COCH_3$), 26.20 ($SiC(CH_3)_3$), 55.44 (2x OCH_3), 60.23 ($CH_2C(5)$), 63.12 (C(5')), 69.32 (C(3')), 76.45 (C(2')), 83.06 (C(4')), 86.19 (tert. C(DMT)), 91.67 (C(1')), 94.22 (C(8)), 98.48 C(5)), 100.94 (C(7)), 113.65 (C(ar)), 127.11 (C(ar)), 128.32 (C(ar)), 129.38 (C(ar)), 129.96 (C(ar)), 130.12 (C(ar)), 135.81 (C(ar)), 136.02 (2x C(ar)-O), 145.12 (C(ar)), 146.68 (C(6)), 152.90 (C(2)), 158.56 (C(ar)), 161.52 (C(4)), 170.87 ($COCH_3$) ppm.

N^4-Acetyl-5-((triisopropylsylil)ethynyl)-5'-O-(4,4'-dimethoxytrityl)-2'-*O*-*tert*-butyldimethylsilyl cytidine-3'-O-2-cyanoethyl-*N,N*-diisopropylphosphoramidite (**11**). Compound **10** (1.17 g, 2.02 mmol) was dissolved in 30 mL anhydrous DCM. DIPEA (0.343 g, 2.65 mmol, 464 µL) was then added followed by. CEP-Cl (0.471 g, 1.99 mmol, 444 µL) and 1-me imidazole (5.45 mg, 6.64 x 10^{-5} mol, 34 µL of a 16% solution in THF). The reaction stirred at rt for approximately 6 hours before being diluted with 200 mL $CHCl_3$ and washed once with 200 mL 50% $NaHCO_3$. The organic layer was dried over Na_2SO_4, evaporated, and the crude product was kept under high vacuum overnight. The crude product was dissolved in DCM and purified by silica gel chromatography (0% then 5% acetone in DCM) pre-equilibrated with 0.1% TEA in DCM. Purification afforded the final product **11** (1.56 g, 1.44 mmol, 71%) as a light yellow foam once dried under high vacuum. Product was aliquoted in 30 mg portions and stored in ABI oligosynthesizer vials under nitrogen at -20°C for use in oligosynthesis. R_f = 0.18 (25% EtOAC in hexane). HR-MS

(ESI-TOF) calculated [MH^+] = 1,082.5618, observed [MH^+] = 1,082.5611. ^{1}H-NMR (400 MHz, DMSO-d6) δ = 0.08 (s, 6H, H_3C-Si), 0.88 (s, 9H, $(H_3C)_3C$-Si), 0.95 (d, 18H, $(H_3C)_2C$-Si), 1.07-1.13 (m, 12H, $(H_3C)_2$-CH), 2.33 (s, 3H, $(H_3C)CO$), 2.78 (t, 2H, H_2C-CN), 3.23 (m, 2H, $(H_3C)_2$-CH), 3.54 (m, 4H, H_2-C(5')), 3.76 (s, 6H, OCH_3), 4.08 (m, 2H, H-C(4'), H-C(3')), 4.31 (m, 1H, H-C(2')), 5.87-5.99 (dd, t, 1H, H-C(1')), 6.89 (m, 4H, H-C(ar)), 7.32-7.44 (m, 9H, H-C(ar)), 8.29-8.59 (dd, H-C(6)), 9.20 (d, 1H, H-N(4)). ^{13}C-NMR (126 MHz, CD_3CN) δ = -4.93, -4.72 (2x $SiCH_3$), 11.41 (3x $SiC(CH_3)_2$), 20.44 ($SiC(CH_3)_3$), 20.60 (CH_2CN), 24.47 (2x N(iPr)), 25.77 ($COCH_3$), 26.16 ($SiC(CH_3)_3$), 43.64 (2x $NC(CH_3)_2$), 55.44 (2x OCH_3), 58.54 ($CH_2C(5)$), 59.38 (C(5')), 72.86 (C(3')), 76.17 C(2')), 83.68 (C(4')), 87.30 (tert. C(DMT)), 91.15 (C(1')), 96.36 (C(8)), 101.01 (C(5)), 102.23 (C(7)), 113.75 (C(ar)), 118.78 (CN), 127.53 (C(ar)), 128.57 (C(ar)), 129.57 (C(ar)), 130.53 (C(ar)), 130.62 (C(ar)), 136.09 (C(ar)), 136.31 (2x C(ar)-O), 145.67 (C(ar)), 148.68 (C(6)), 153.98 (C(2)), 159.29 (C(ar)), 160.63 (C(4)), 171.28 ($COCH_3$) ppm. ^{31}P-NMR (162 MHz, DMSO-d6) δ = 148.57, 148.77ppm.

Spectral characterization data for 5-EC (3). **(A)** ^{1}H (500 MHz, DMSO-d_6) and APT ^{13}C (126 MHz, DMSO-d_6) NMR spectra of 5-EC . **(B)** Positive mode high-resolution mass spectrum (HRMS) for 5-EC. The Na^+ ion is detected instead of the H^+ ion.

A

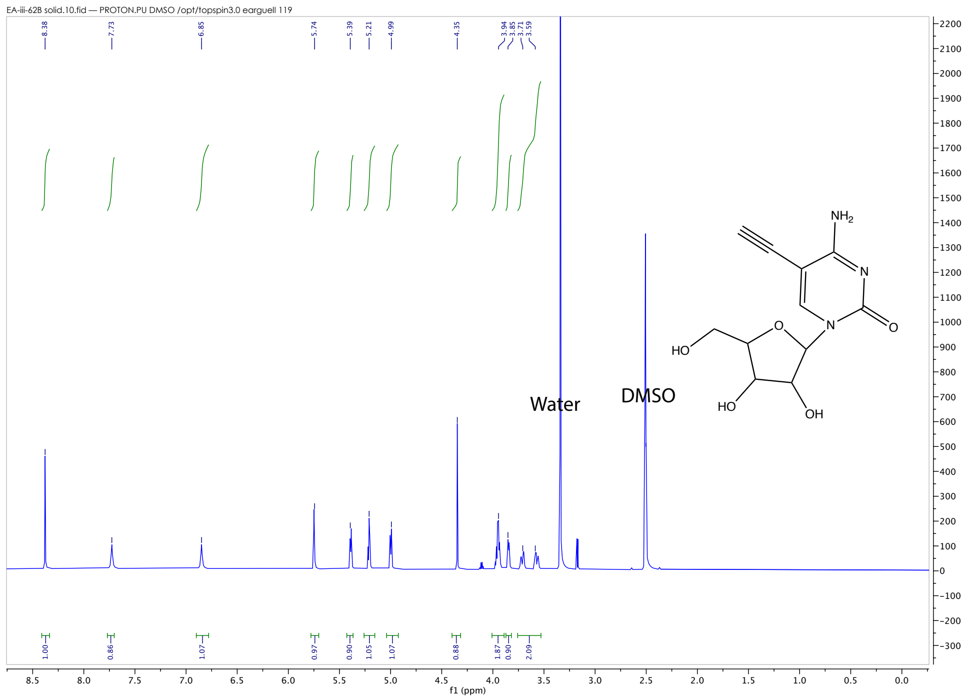

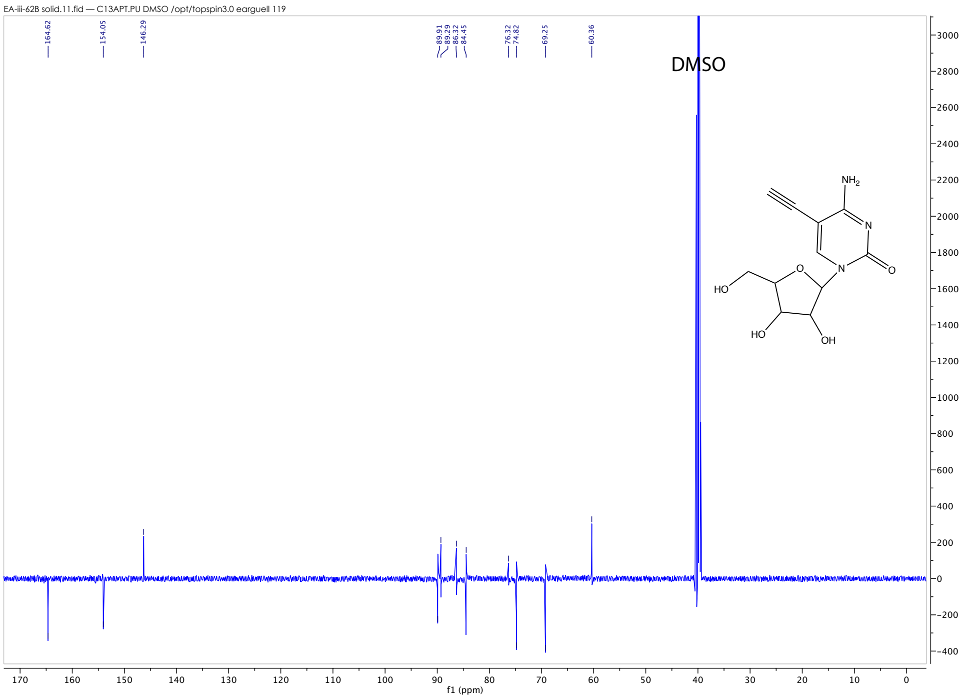

B

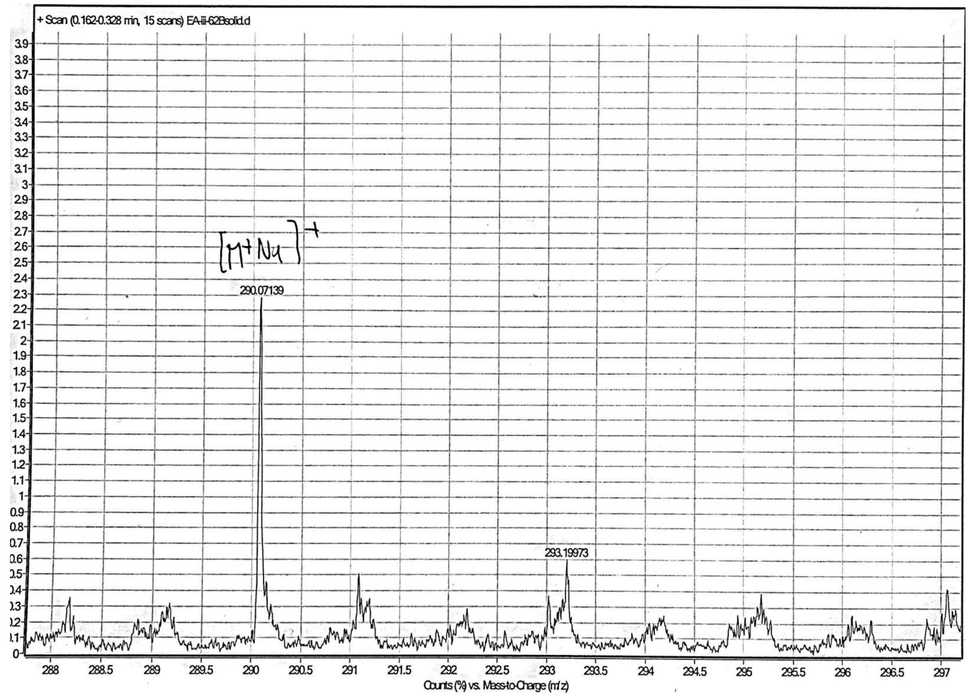
+ Scan (0.162-0.328 min, 15 scans) EA-iii-62Bsolid.d
[M+Na]+
290.07139
293.19973
Counts (%) vs. Mass-to-Charge (m/z)

Spectral characterization of 5. (A) ^{1}H (500 MHz, $CDCl_3$) NMR and **(B)** APT ^{13}C (126 MHz, $CDCl_3$)

A

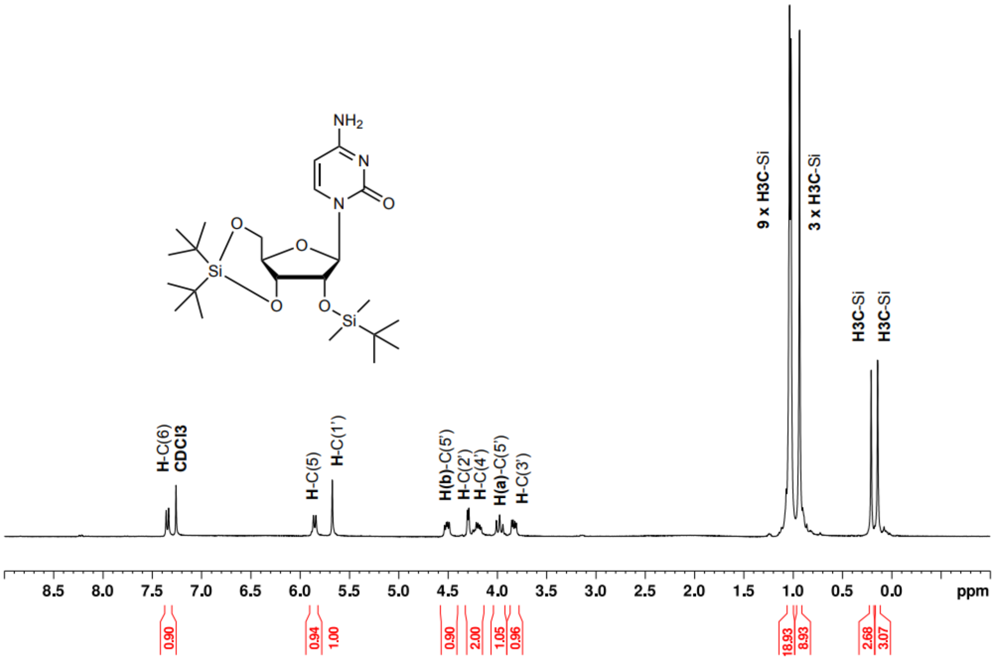

B

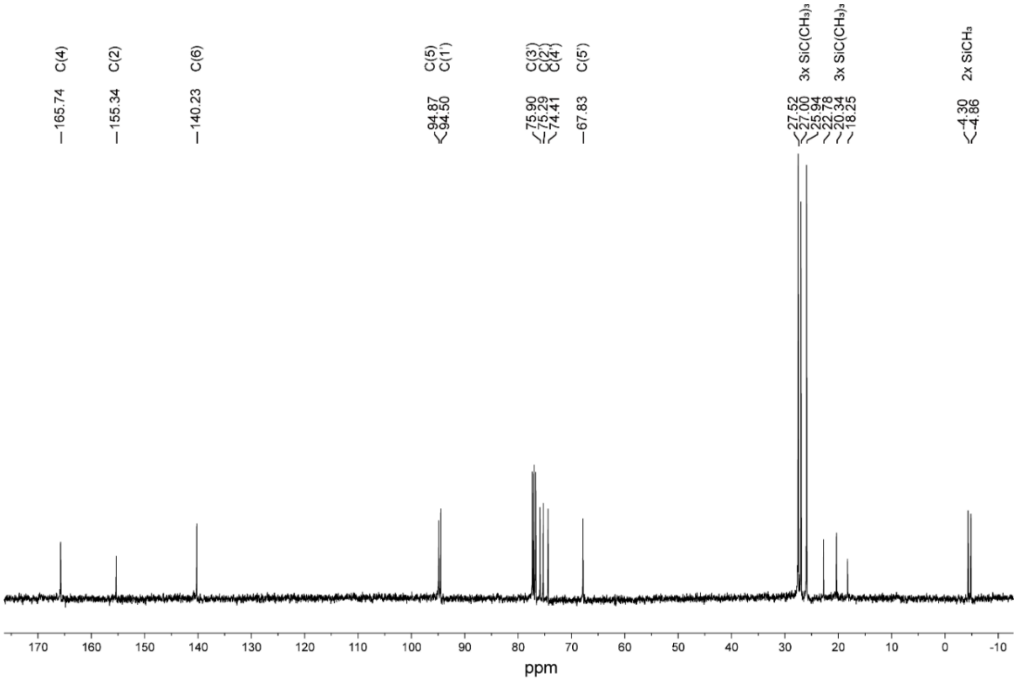

Spectral characterization of 6. (A) ^{1}H (500 MHz, $CDCl_3$) NMR and **(B)** APT ^{13}C (126 MHz, $CDCl_3$)

A

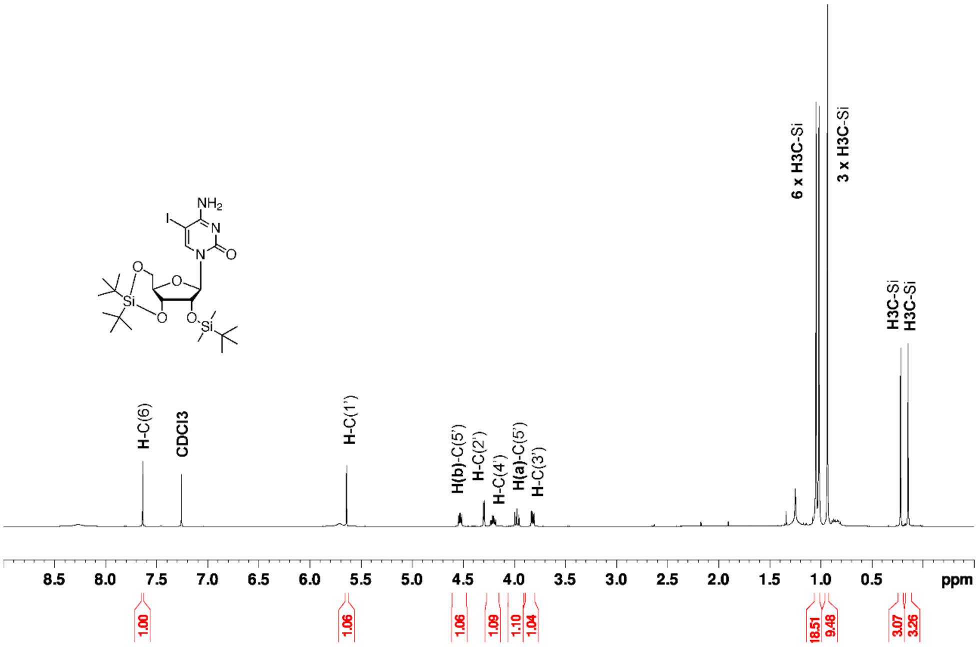

B

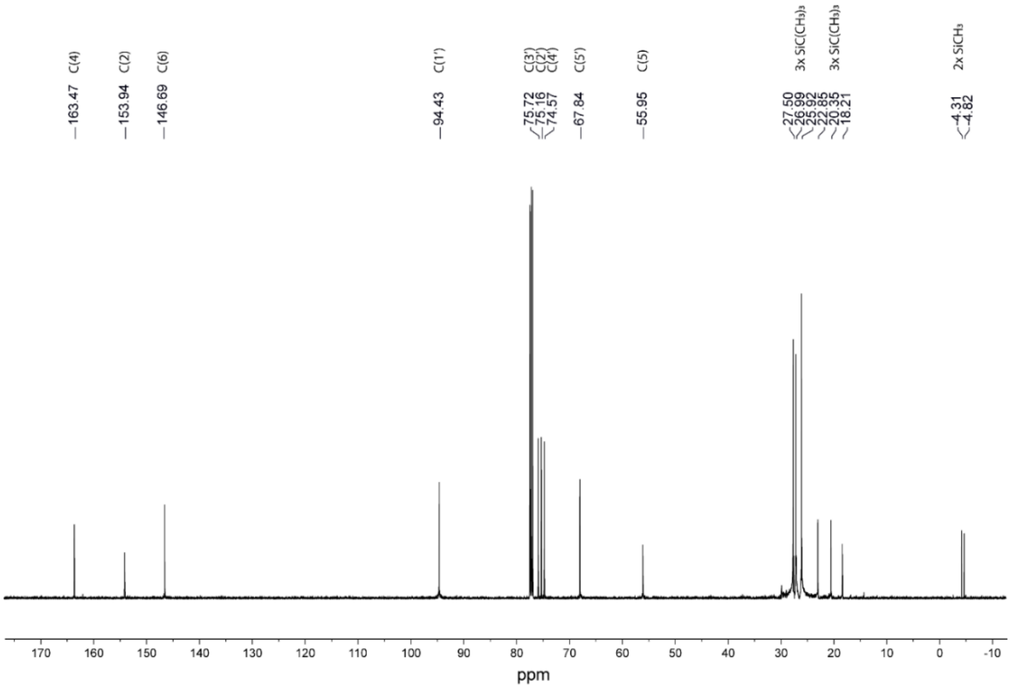

Spectral characterization of 7. (A) ^{1}H (500 MHz, $CDCl_3$) NMR and **(B)** APT ^{13}C (126 MHz, $CDCl_3$)

A

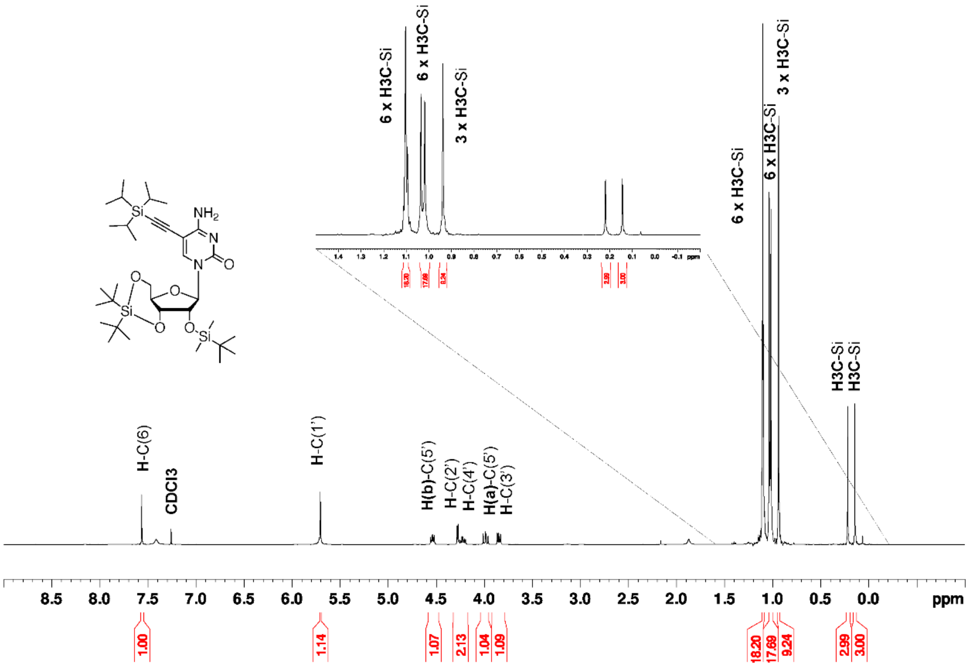

B

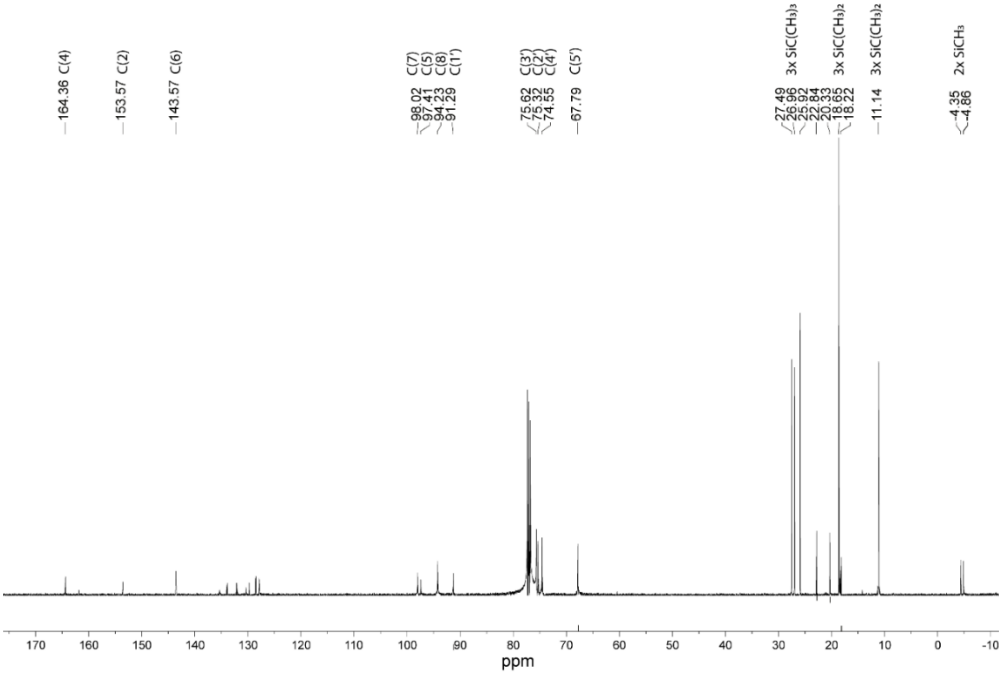

Spectral characterization of 8. (A) ^{1}H (500 MHz, $CDCl_3$) NMR and **(B)** APT ^{13}C (126 MHz, $CDCl_3$)

A

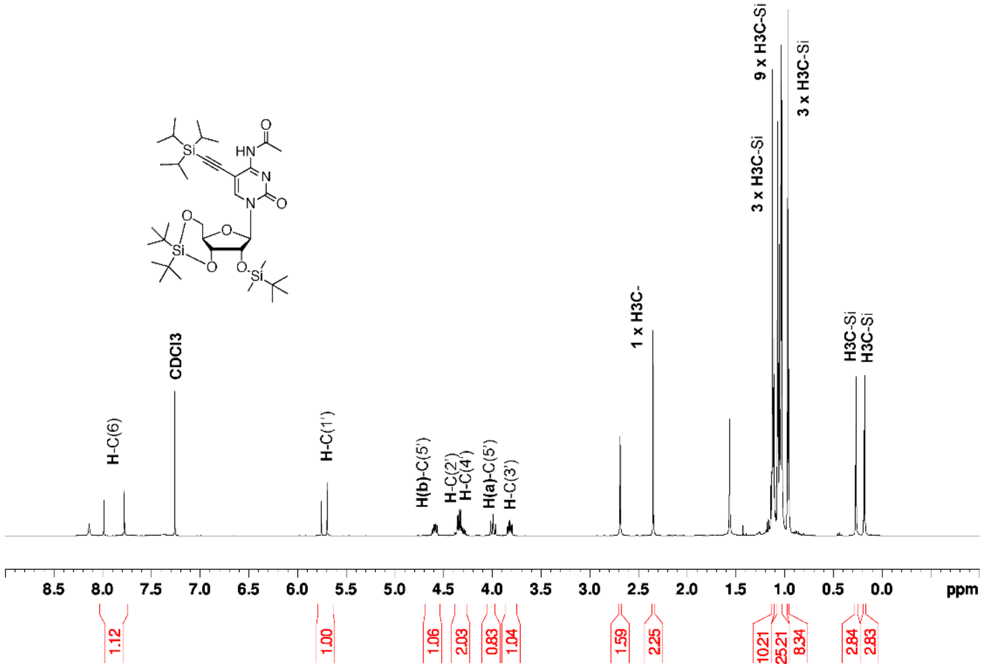

B

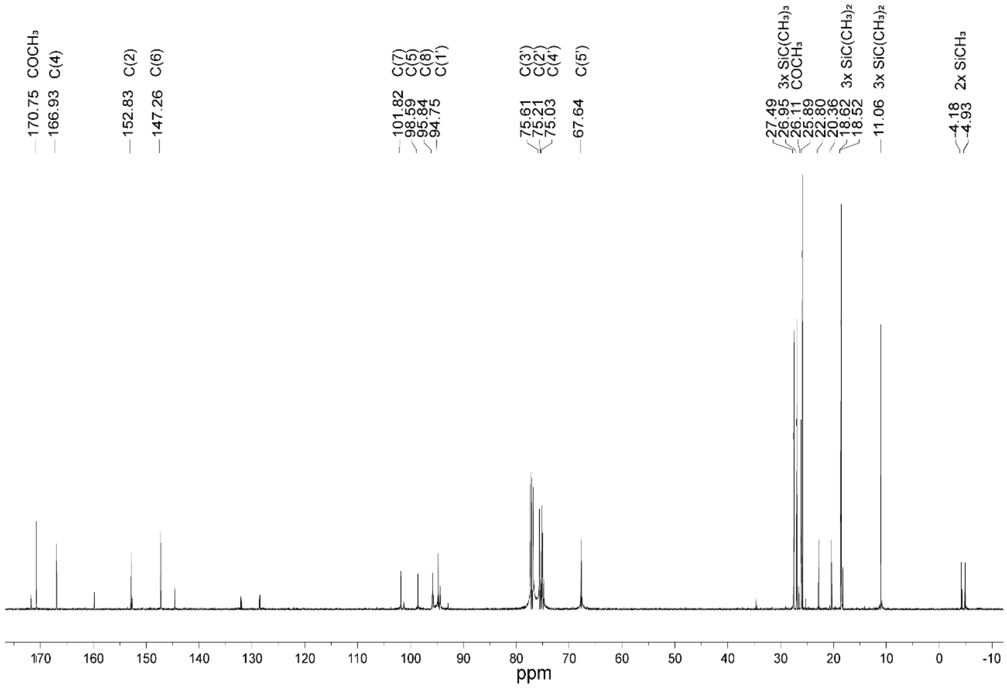

Spectral characterization of 9. (A) ^{1}H (500 MHz, $CDCl_3$) NMR and **(B)** APT ^{13}C (126 MHz, $CDCl_3$)

A

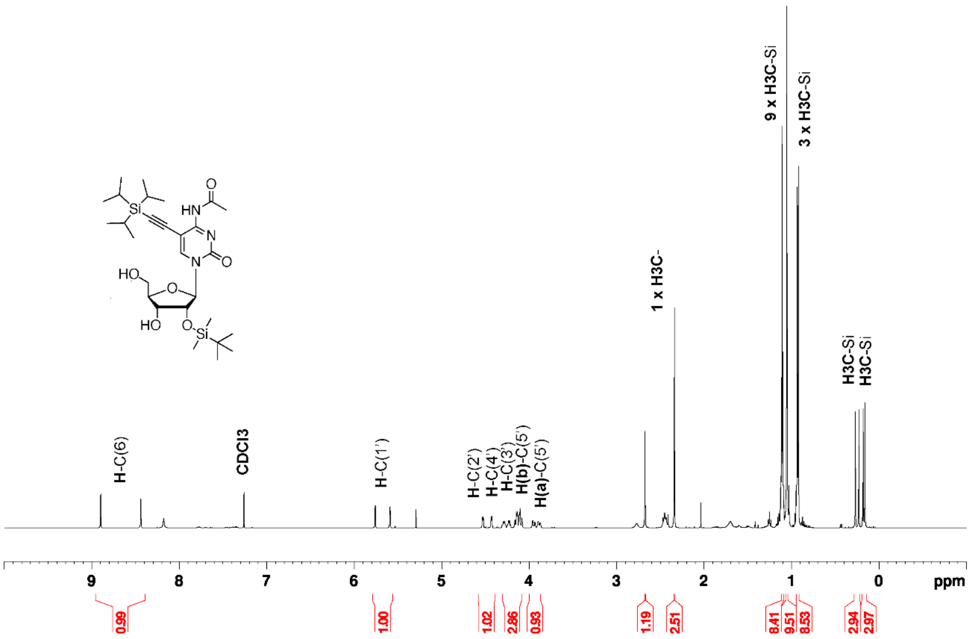

B

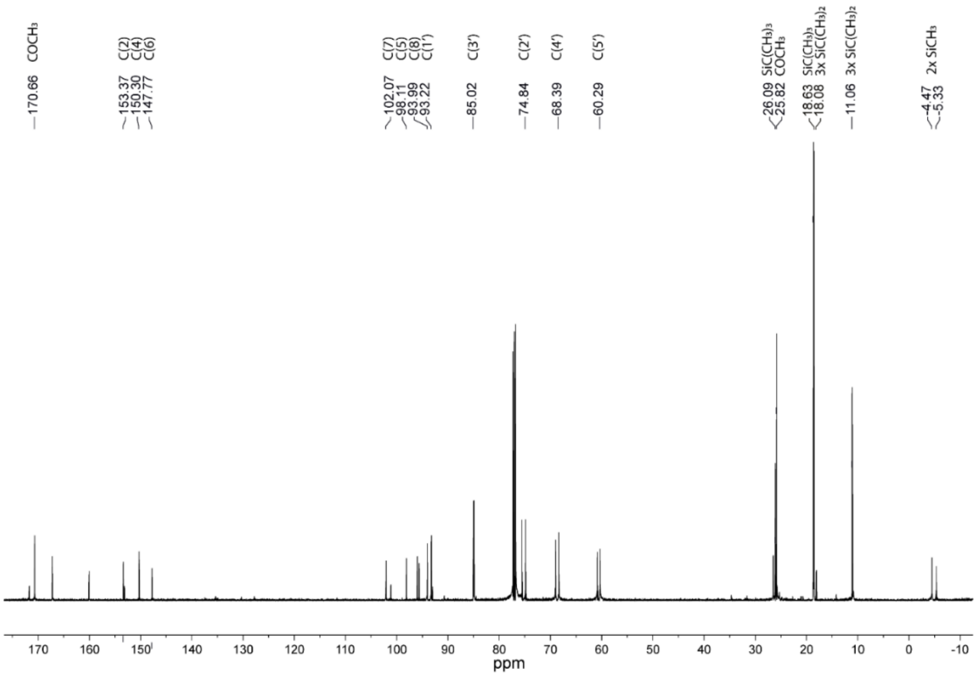

Spectral characterization of 10. **(A)** ^{1}H (500 MHz, DMSO-d_6) NMR and **(B)** APT ^{13}C (126 MHz, DMSO-d_6)

A

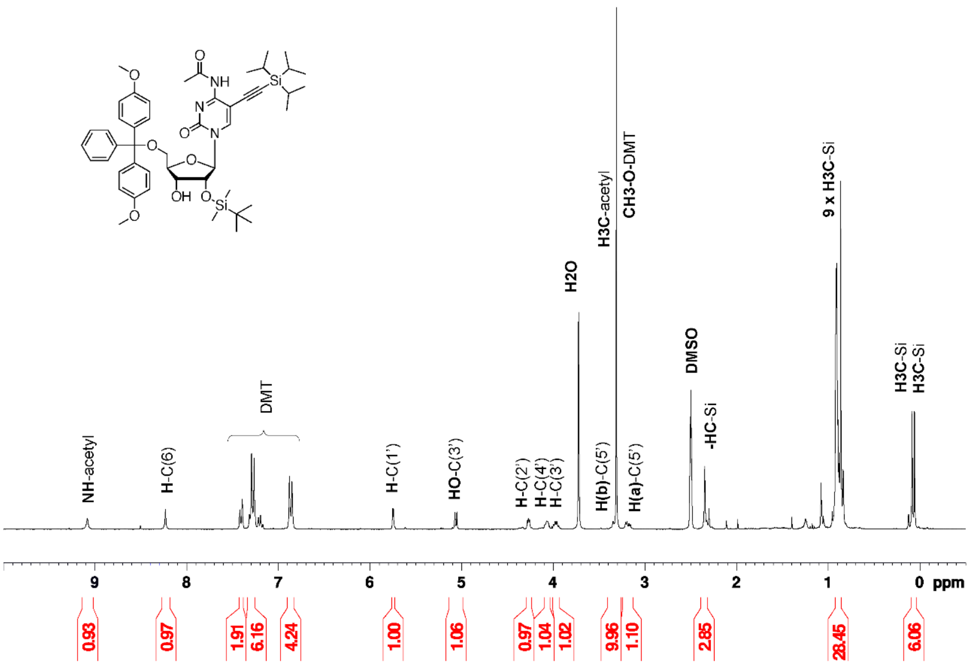

B

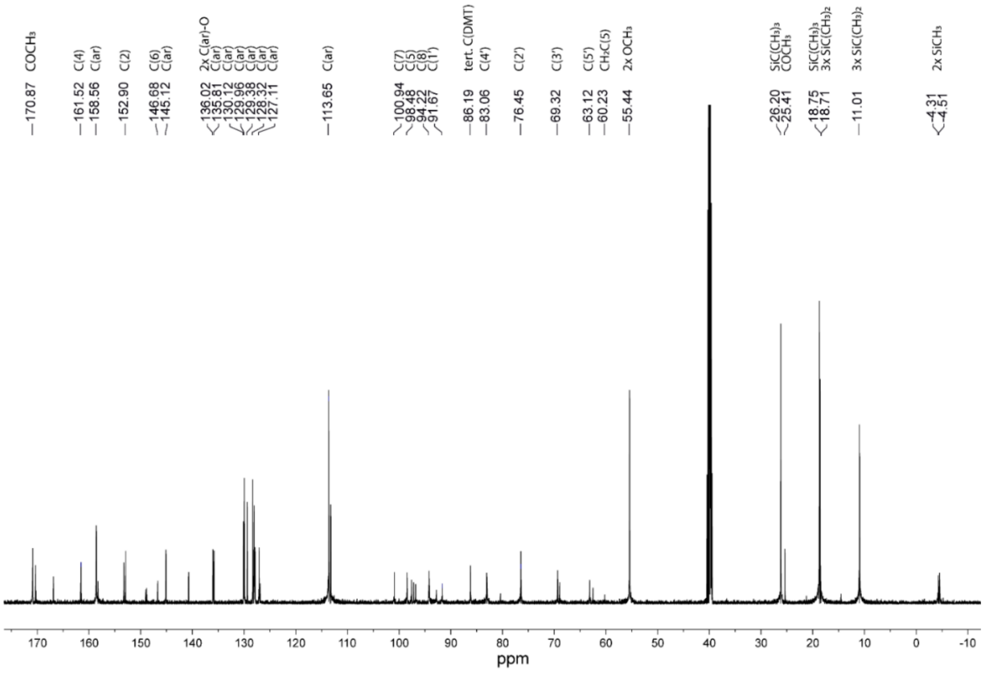

Spectral characterization of 11. **(A)** ^{1}H (500 MHz, DMSO-d_6) NMR, **(B)** APT ^{13}C NMR (126 MHz, CD_3CN) and **(C)** ^{31}P (162 MHz, DMSO-d_6) NMR spectra.

A

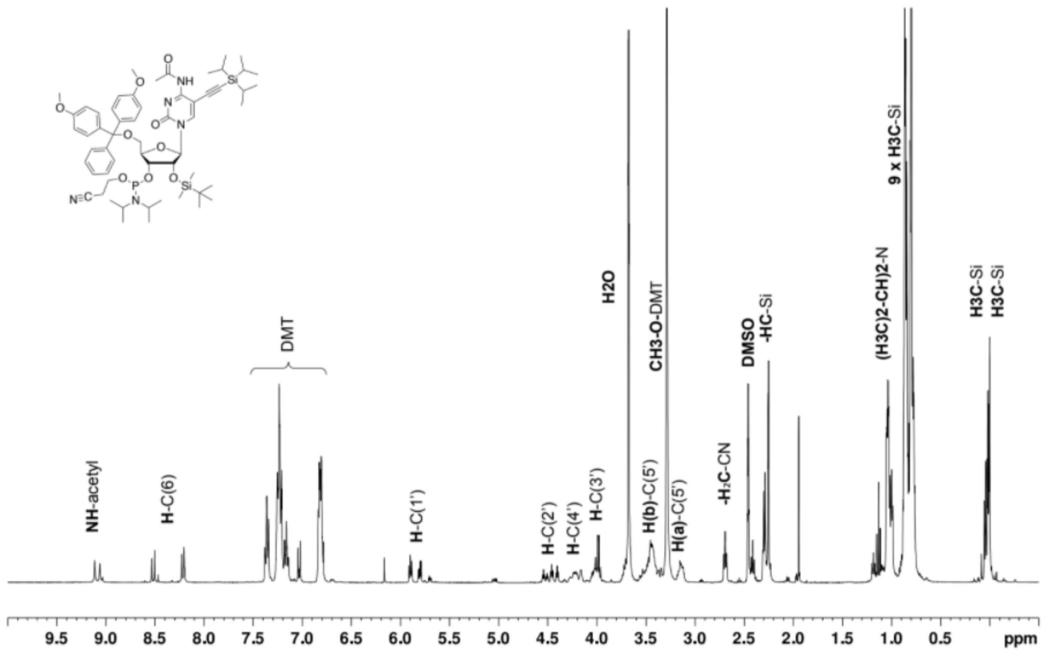

B

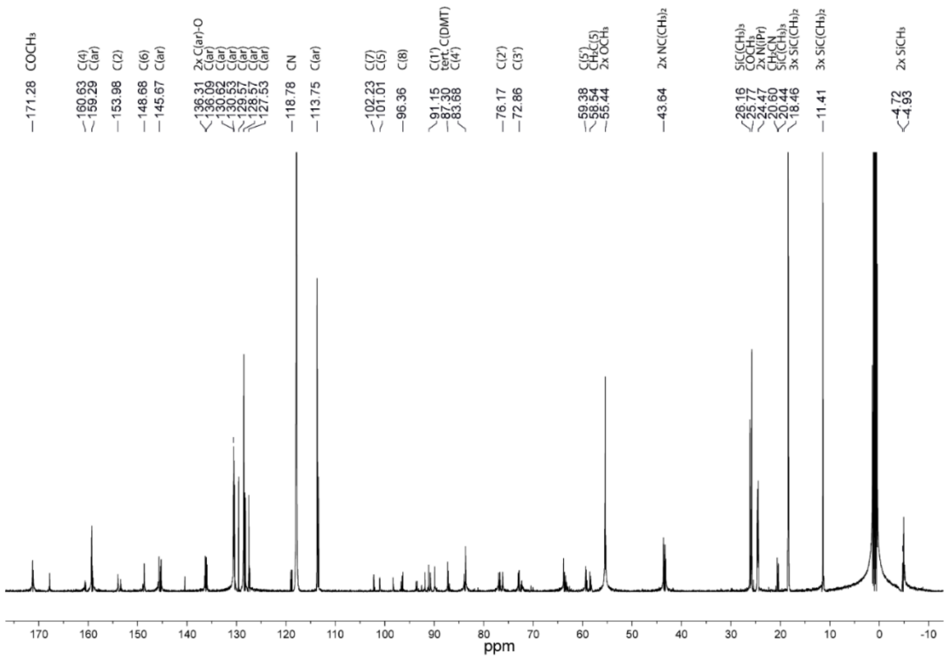

C

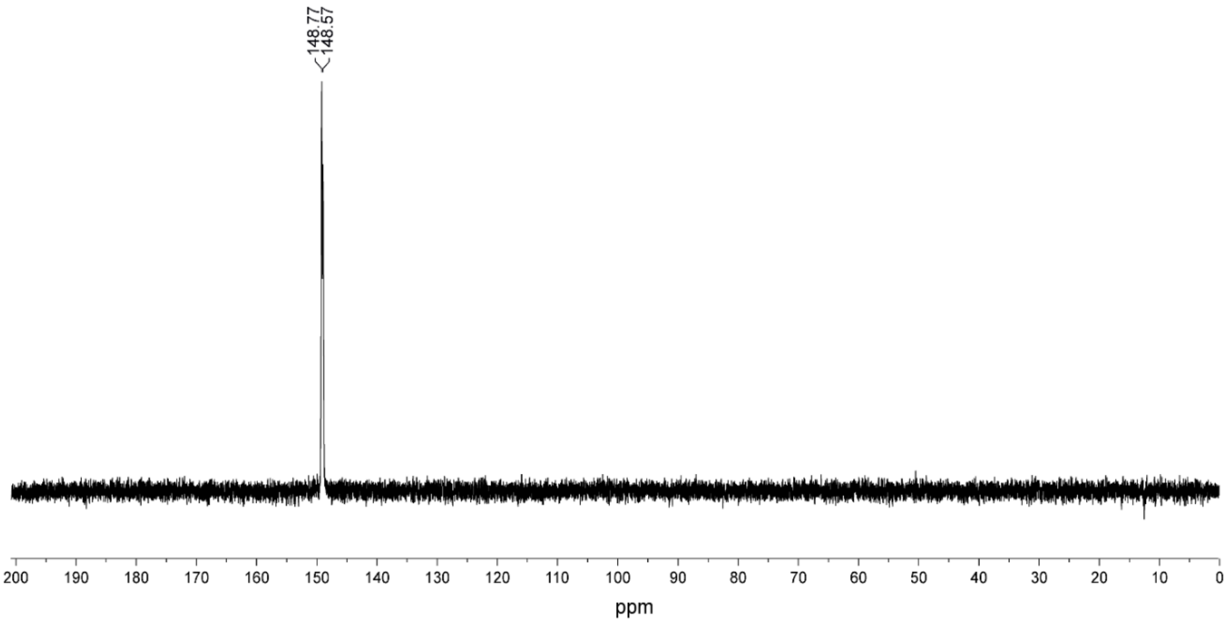

148.77
148.57
200
190
180
170
160
150
140
130
120
110
100
90
80
70
60
50
40
30
20
10
0
ppm

References:

1. Li, X.; Xiong, X.; Wang, K.; Wang, L.; Shu, X.; Ma, S.; Yi, C., Transcriptome-wide mapping reveals reversible and dynamic N(1)-methyladenosine methylome. Nat Chem Biol 2016, 12 (5), 311-6.
