# Supplementary material for: Reactivity-dependent profiling of RNA 5-methylcytidine dioxygenases
Source: Nat Commun. 2022 Jul 19;13:4176. doi: 10.1038/s41467-022-31876-2 (PMC9296451; doi:10.1038/s41467-022-31876-2)
Supplement: Supplementary file 2 — Description of Additional Supplementary Files [file 41467_2022_31876_MOESM2_ESM.pdf]

Description of Additional Supplementary Files

File Name: Supplementary Data 1
Description: 5-EC RNABPP proteomics data

File Name: Supplementary Data 2
Description: 5-EC iCLIP data

File Name: Supplementary Data 3
Description: ARP sequencing data

File Name: Supplementary Data 4
Description: Pyridine borane sequencing data.
