# Supplementary material for: Reactivity-dependent profiling of RNA 5-methylcytidine dioxygenases
Source: Nat Commun. 2022 Jul 19;13:4176. doi: 10.1038/s41467-022-31876-2 (PMC9296451; doi:10.1038/s41467-022-31876-2)
Supplement: Supplementary file 7 — Reporting Summary [file 41467_2022_31876_MOESM7_ESM.pdf]

nature portfolio

Corresponding author(s): Ralph E. Kleiner

Last updated by author(s): 2022/06/16

Reporting Summary

Nature Portfolio wishes to improve the reproducibility of the work that we publish. This form provides structure for consistency and transparency in reporting. For further information on Nature Portfolio policies, see our Editorial Policies and the Editorial Policy Checklist.

Statistics

For all statistical analyses, confirm that the following items are present in the figure legend, table legend, main text, or Methods section.

n/a	Confirmed	
☐	☒	The exact sample size (*n*) for each experimental group/condition, given as a discrete number and unit of measurement
☐	☒	A statement on whether measurements were taken from distinct samples or whether the same sample was measured repeatedly
☐	☒	The statistical test(s) used AND whether they are one- or two-sided<br>*Only common tests should be described solely by name; describe more complex techniques in the Methods section.*
☒	☐	A description of all covariates tested
☒	☐	A description of any assumptions or corrections, such as tests of normality and adjustment for multiple comparisons
☐	☒	A full description of the statistical parameters including central tendency (e.g. means) or other basic estimates (e.g. regression coefficient) AND variation (e.g. standard deviation) or associated estimates of uncertainty (e.g. confidence intervals)
☐	☒	For null hypothesis testing, the test statistic (e.g. *F*, *t*, *r*) with confidence intervals, effect sizes, degrees of freedom and *P* value noted<br>*Give P values as exact values whenever suitable.*
☒	☐	For Bayesian analysis, information on the choice of priors and Markov chain Monte Carlo settings
☒	☐	For hierarchical and complex designs, identification of the appropriate level for tests and full reporting of outcomes
☒	☐	Estimates of effect sizes (e.g. Cohen's *d*, Pearson's *r*), indicating how they were calculated

Our web collection on statistics for biologists contains articles on many of the points above.

Software and code

Policy information about availability of computer code

Data collection	Proteomics data was collected using Orbitrap Lumos Fusion version 3.3.2782.34 (ThermoFisher)<br>LC-MS/MS data was collected using Agilent MassHunter Workstation Data Acquisition version 10.0 (Agilent)<br>Fluoresence Microscopy data was collected using NIS Elements AR software version 8.0.2 (Nikon)<br>iCLIP and ARP-seq data were processed in the iCount primary analysis and Paraclu pipelines on the iMAPS web server (https://imaps.genialis.com/iclip).Reads were mapped to STAR(v.2.7.0f). Paraclu (2.0.3) were used for peak calling.<br>Codes and software for sequencing data collection was described in the methods section.
Data analysis	Proteomics analysis was performed using Scaffold4 version 4.9.0 (Proteome Software Inc.)<br>LC-MS/MS data was analuzed using Agilent MassHunter Qualitative Analysis version 10.0 and QQQ Quantitative Analysis version 10.0 (Agilent)<br>Multiple t-tests for proteomics data and unpaired t-tests for LC-MS/MS data were done in GraphPad Prism version 9.2.0. (GraphPad Software L.L.C.)<br>iCLIP and ARP-seq data were processed in the iCount primary analysis and Paraclu pipelines on the iMAPS web server (https://imaps.genialis.com/iclip).Reads were mapped to STAR(v.2.7.0f). Paraclu (2.0.3) were used for peak calling. Motifs were generated using MEME Suite version 5.3.3.<br>For Pyridine borane amplicon sequencing , Pileup files were generated using Samtools mpileup (Galaxy Version 2.1.4) with default parameters. The variants were called using VarScan mpileup (Galaxy Version 2.4.3.1).

For manuscripts utilizing custom algorithms or software that are central to the research but not yet described in published literature, software must be made available to editors and reviewers. We strongly encourage code deposition in a community repository (e.g. GitHub). See the Nature Portfolio guidelines for submitting code & software for further information.

Data

Policy information about availability of data

All manuscripts must include a data availability statement. This statement should provide the following information, where applicable:

- Accession codes, unique identifiers, or web links for publicly available datasets
- A description of any restrictions on data availability
- For clinical datasets or third party data, please ensure that the statement adheres to our policy

Mass spectrometry proteomics data is provided in a separate Excel file. Proteomics data has also been submitted to PRIDE (PXD029955).
For nucleoside LC-MS experiment, nucleoside concentrations and calculated ratios are provided in SI. Examples of chromatograms and calibration curves are also provided in SI.
iCLIP and ARP sequencing data reported in this paper have been deposited in the NCBI Gene expression omnibus (accession code: GSE202815).
Pyridine borane sequencing data is is provided as a supplementary file in an Excel sheet.

Field-specific reporting

Please select the one below that is the best fit for your research. If you are not sure, read the appropriate sections before making your selection.

[x] Life sciences [ ] Behavioural & social sciences [ ] Ecological, evolutionary & environmental sciences

For a reference copy of the document with all sections, see nature.com/documents/nr-reporting-summary-flat.pdf

Life sciences study design

All studies must disclose on these points even when the disclosure is negative.

Sample size	For LC-MS proteomics experiment, LC-QQQ-MS experiments, n = 3 was selected to offer sufficient statistical power. For IF and imaging experiments n = 2 was selected to guarantee the consistency of the images. For high-throughput sequencing experiment, n = 2 was selected to offer sufficient statistical power.Sample size are all indicated in the figure legends. The sample size was determined based on the previous studies in the same research fields.
Data exclusions	No data was excluded for experiments in this manuscript.
Replication	All experiments were independently replicated, with biological and/or technical replicates listed in the legends of the corresponding figures. All attempts at replication were successful.
Randomization	No randomization was performed because we assumed little selection bias in cell culture.
Blinding	No blinding was performed as there was no need to prevent the study participants (cell culture) from knowing the treatment conditions. Data collection was largely performed using automated analysis software.

Reporting for specific materials, systems and methods

We require information from authors about some types of materials, experimental systems and methods used in many studies. Here, indicate whether each material, system or method listed is relevant to your study. If you are not sure if a list item applies to your research, read the appropriate section before selecting a response.

Materials & experimental systems

n/a	Involved in the study	
[ ]	[x]	Antibodies
[ ]	[x]	Eukaryotic cell lines
[x]	[ ]	Palaeontology and archaeology
[x]	[ ]	Animals and other organisms
[x]	[ ]	Human research participants
[x]	[ ]	Clinical data
[x]	[ ]	Dual use research of concern

Methods

n/a	Involved in the study	
[x]	[ ]	ChIP-seq
[x]	[ ]	Flow cytometry
[x]	[ ]	MRI-based neuroimaging

Antibodies

Antibodies used	Monoclonal ANTI-FLAG® M2 antibody produced in mouse was purchased from Sigma (catalog #F1804,1:1000).<br>Anti-β-actin (mouse) antibody was purchased from Cell Signaling (catalog #3700,1:10000).

	rabbit anti-ALKBH1 was purchased from Abcam (Abcam #128895,1:2000).<br>Anti-TET2 were purchased form Novus(Novus #NBP2-32104,0.2ug/ml).<br>Anti-NSUN2 rabbit polyclonal antibody was purchased from proteintech (catalog #20854-1-AP, lot #00047872,1:2000).
Validation	Validation of anti-FLAG M2 antibody can be obtained from Sigma website: https://www.sigmaaldrich.com/catalog/product/sigma/f1804?lang=en&region=US<br>Validation of anti-β-actin antibody can be obtained from Cell Signaling website: https://www.cellsignal.com/products/primary-antibodies/b-actin-8h10d10-mouse-mab/3700<br>Validation of anti-NSUN2 antibody can be obtained from proteintech website: https://www.ptglab.com/products/NSUN2-Antibody-20854-1-AP.html<br>Validation of anti-ALKBH1 antibody can be obtained from https://www.abcam.com/alkbh1-antibody-epr61752-ab128895.html<br>Validation of anti-TET2 antibody can be obtained from https://www.novusbio.com/products/tet2-antibody_nbp2-32104

Eukaryotic cell lines

Policy information about cell lines

Cell line source(s)	HEK293T WT cells (ATCC #CRL-3216) were a gift from Tom Muir at Princeton University.<br>Flp-In T-Rex 293 cells (ThermoFisher cat# R78007) were a gift from John LaCava at The Rockefeller University.<br>Knockout cell lines and stable cell lines expressing 3x-FLAG tagged constructs were generated in our lab.
Authentication	Stable cell lines expressing 3x-FLAG tagged constructs were confirmed by western blot.<br>Knockout cell lines were confirmed by genomic PCR and western blot.<br>HEK293T WT and parent Flp-In cell lines were not authenticated.
Mycoplasma contamination	Parent cell lines were tested for mycoplasma contamination.
Commonly misidentified lines<br>(See ICLAC register)	No commonly misidentified cell lines were used.
